# LIPID DROPLET PROTEIN OF SEEDS is involved in the control of lipid droplet size in Arabidopsis seeds and seedlings

**DOI:** 10.1093/plcell/koaf121

**Published:** 2025-05-15

**Authors:** Nathan M Doner, Alyssa C Clews, Nicolas Esnay, Payton S Whitehead, You Wang, Trevor B Romsdahl, Damien Seay, Philipp W Niemeyer, Martin Bonin, Yang Xu, Oliver Valerius, Gerhard H Braus, Till Ischebeck, Kent D Chapman, John M Dyer, Robert T Mullen

**Affiliations:** Department of Molecular and Cellular Biology, University of Guelph, Guelph, ON, Canada N1G 2W1; Department of Molecular and Cellular Biology, University of Guelph, Guelph, ON, Canada N1G 2W1; BioDiscovery Institute and Department of Biological Sciences, University of North Texas, Denton, TX 76203, USA; BioDiscovery Institute and Department of Biological Sciences, University of North Texas, Denton, TX 76203, USA; Department of Molecular and Cellular Biology, University of Guelph, Guelph, ON, Canada N1G 2W1; BioDiscovery Institute and Department of Biological Sciences, University of North Texas, Denton, TX 76203, USA; United States Department of Agriculture, Agriculture Research Service, Arid-Land Agricultural Research Center, Maricopa, AZ 85138, USA; Department of Plant Biochemistry, Albrecht-von-Haller-Institute for Plant Sciences, University of Göttingen, 37077 Göttingen, Germany; Institute of Plant Biology and Biotechnology, Green Biotechnology, University of Münster, 48149 Münster, Germany; Department of Molecular and Cellular Biology, University of Guelph, Guelph, ON, Canada N1G 2W1; Institute for Microbiology and Genetics, Göttingen Center for Molecular Biosciences and Campus Institute for Molecular Microbiology and Genetics, University of Göttingen, 37077 Göttingen, Germany; Institute for Microbiology and Genetics, Göttingen Center for Molecular Biosciences and Campus Institute for Molecular Microbiology and Genetics, University of Göttingen, 37077 Göttingen, Germany; Institute of Plant Biology and Biotechnology, Green Biotechnology, University of Münster, 48149 Münster, Germany; BioDiscovery Institute and Department of Biological Sciences, University of North Texas, Denton, TX 76203, USA; United States Department of Agriculture, Agricultural Research Service, Southern Regional Research Center, New Orleans, LA 70124, USA; Department of Molecular and Cellular Biology, University of Guelph, Guelph, ON, Canada N1G 2W1

## Abstract

In oilseeds, energy-rich carbon is stored as triacylglycerols in organelles called lipid droplets (LDs). While several of the major biogenetic proteins involved in LD formation have been identified, the full repertoire of LD proteins and their functional roles remains incomplete. Here, we show that the low-abundance, seed-specific LD protein LIPID DROPLET PROTEIN OF SEEDS (LDPS) contains an amphipathic α-helix and proline hairpin motif that serves as an LD-targeting signal and a separate region that binds to the LD protein OLEOSIN 1 (OLEO1). Loss of LDPS function results in smaller LDs and less seed oil in comparison with wild type, while overexpression of *LDPS* results in an increase in LD size and seed oil content. Loss of LDPS function also results in an inability of LDs to undergo fusion during postgerminative seedling growth. Analysis of *oleo1* and *ldps* single- and double-mutant seeds and freeze–thaw treatment of seeds revealed that OLEO1 suppresses the ability of LDPS to promote larger LDs. Collectively, our results identify LDPS as an important player in LD biology that functions together with OLEO1 to determine LD size in Arabidopsis (*Arabidopsis thaliana*) seeds and seedlings through a process that involves LD–LD fusion.

## Teaser

LIPID DROPLET PROTEIN OF SEEDS cooperates with OLEOSIN1 to influence lipid droplet size in Arabidopsis seeds and seedlings.

## Introduction

Lipid droplets (LDs) are evolutionarily conserved organelles found in all kingdoms of life (reviewed in Chapman et al. 2012; Murphy 2012; Lundquist et al. 2020). They play a central role in lipid metabolism through the long- or short-term storage of neutral lipids, functioning as important carbon and energy reserves or as depots for fatty acids and lipids produced during membrane remodeling (Shao et al. 2019; Krawczyk et al. 2022a; Bouchnak et al. 2023). LDs have a unique organellar structure composed of a single phospholipid monolayer surrounding a core of neutral lipids, typically triacylglycerols (TAGs) and sterol esters. The surface of LDs also is coated with numerous proteins, and the composition of these protein constituents helps define the biological and physiological role(s) of LDs in various tissues and cell types.

In plants, LDs have been shown to be involved in numerous functions, including germination and postgerminative seedling growth, abiotic and biotic stress responses, stomatal opening/closing, and pollen tube growth (Huang 2018; Ischebeck et al. 2020; Bouchnak et al. 2023; Cai and Horn 2025). Several proteins involved in LD biogenesis in plant cells have been identified, including SEIPIN, which is an endoplasmic reticulum (ER)-localized membrane protein involved in production of nascent LDs at the ER surface (Cai et al. 2015), and the LD-ASSOCIATED PROTEINS (LDAPs) (Horn et al. 2013; Gidda et al. 2016; Kim et al. 2016), LDAP-INTERACTING PROTEIN (LDIP) (Brocard et al. 2017; Pyc et al. 2017), and OLEOSINS (OLEOs) (Qu et al. 1986), which are all important coat proteins required in different amounts and in different plant tissues for proper LD formation and maintenance (reviewed in Guzha et al. 2023).

The evolution of plant seeds included numerous innovations in cellular processes to meet the unique challenges and demands presented during this stage of plant life, including seed desiccation and dispersal (de Vries and Ischebeck 2020). Storage oil synthesis in plant seeds occurs over a relatively short developmental period (i.e. embryogenesis), requiring the synthesis and packaging of large amounts of TAG in the cytoplasm of cells. In oilseeds, such as in the model plant Arabidopsis (*Arabidopsis thaliana*), TAG biosynthetic processes are upregulated by seed-specific transcriptional factors, such as WRINKLED1 and LEAFY COTYLEDON2 (LEC2) (Kong et al. 2019; Liu et al. 2021). To help package the oil efficiently, plants, unlike most other eukaryotes, contain more than 1 isoform of the LD biogenetic protein SEIPIN, of which the SEIPIN1 isoform is predominantly expressed in developing seeds (Cai et al. 2015; Taurino et al. 2018). Oilseeds also produce high amounts of OLEOs, which serve as the predominant coat proteins on seed LDs and help protect the TAG core and maintain structural integrity of LDs (Huang 2018). OLEOs are encoded by a 17-member gene family in Arabidopsis (Kim et al. 2002), and several members are uniquely expressed in seeds or pollen grains (Kim et al. 2002), both of which undergo desiccation and quiescence. Notably, loss of *OLEO* results in larger LDs in seeds that are more susceptible to LD–LD fusion (Siloto et al. 2006; Shimada et al. 2008; Miquel et al. 2014), particularly during stresses, such as desiccation or freezing, which impair seed germination (Leprince et al. 1997; Shimada et al. 2008). These and other data suggest that OLEOs evolved, at least in part, to help stabilize and shield the LD surface and prevent membrane fusion, particularly in cells that are packed with LDs (Heneen et al. 2008; Schmidt and Herman 2008; and reviewed in Huang 2018).

At the cellular level, oilseeds are enriched in LDs, making it straightforward to isolate LDs and characterize their protein and lipid composition. Among the best-studied seed LD proteins are the caleosins (CLOs) and steroleosins, which are calcium-binding peroxygenases and hydroxysteroid dehydrogenases (HSDs), respectively, as well as the OLEOs, which are among the most abundant LD coat proteins in Arabidopsis and many other plant species (Huang 2018; Hanano et al. 2023). Unlike other LD coat proteins that target directly to the LD surface after their synthesis in the cytoplasm (Dhiman et al. 2020; Olarte et al. 2022), OLEOs are cotranslationally synthesized on the ER (Beaudoin and Napier 2002; Leznicki et al. 2022) and then target to a nascent LD by the partitioning of a hydrophobic stem-loop structure within the protein into the growing hydrophobic TAG core (Abell et al. 2002; 2004; Li et al. 2002; Huang and Huang 2017). The N- and C-terminal regions of OLEOs flanking the central stem-loop structure are more amphiphilic in nature and associate with the phospholipid monolayer of the LD and may interact with other LD proteins (Tzen et al. 1992; Abell et al. 1997, 2002). OLEO-coated LDs are thought to bud from the ER and accumulate in the cytoplasm, whereby the size of the nascent LD is determined, at least in part, by the amount of OLEO protein (Siloto et al. 2006). OLEOs serve also to protect the LD from TAG-degradative enzymes and stabilize LDs during seed desiccation. Upon germination, OLEOs are rapidly degraded by processes that require peroxisomal MYB30-INTERACTING E3 LIGASE 1, type-II metacaspase proteases, and LD-associated PLANT UBX DOMAIN-CONTAINING PROTEIN 10 (PUX10), which together promote the ubiquitin-mediated turnover of OLEOs and other LD coat proteins (Deruyffelaere et al. 2015 , 2018; Kretzschmar et al. 2018; Traver and Bartel 2023; Liu et al. 2024). Thereafter, the denuded LDs are more amendable to TAG breakdown (Zienkiewicz and Zienkiewicz 2020) and undergo LD–LD fusion, resulting in a transient increase in LD size during postgerminative seedling growth (Miquel et al. 2014).

How OLEOs regulate the size of LDs is not well understood. For many years, it was thought that OLEOs alone were the key determinant of LD size during LD biogenesis. However, other proteins, such as SEIPIN, VESICLE-ASSOCIATED MEMBRANE PROTEIN-ASSOCIATED PROTEIN 27-1 (VAP27-1), LDIP, LDAP, and OIL BODY ASSOCIATED PROTEIN 1, are now known to also influence LD size in seeds (López-Ribera et al. 2014 ; Gidda et al. 2016; Brocard et al. 2017; Pyc et al. 2017, 2021; Greer et al. 2020). The identification and characterization of these and other additional protein players have been enabled, in most part, through the development of more sensitive and comprehensive methods for LD protein identification. Indeed, a recent mass spectrometry (MS)-based proteomics analysis of LDs isolated from several stages of Arabidopsis seed development, germination, and seedling establishment, as well as in response to stress, has revealed numerous other potential LD proteins (Kretzschmar et al. 2020; Omata et al. 2024; Scholz et al. 2025). One of the LD proteins identified was SEED LIPID DROPLET PROTEIN 1, which facilitates the tethering of LDs to the plasma membrane in seedlings (Krawczyk et al. 2022b). Another was LIPID DROPLET PROTEIN OF SEEDS (LDPS), which was named as such because, based on the Arabidopsis eFP Browser at the Bio-Analytic Resource for Plant Biology (BAR; Winter et al. 2007 ), the gene is expressed in Arabidopsis exclusively in developing and mature seeds, and proteomics analysis revealed that it was a low-abundance LD protein with highest accumulation in imbibed seeds (Kretzschmar et al. 2020). LDPS is also annotated at The Arabidopsis Information Resource (TAIR) (Berardini et al. 2015) as a so-called Broad-complex, Tramtrack and Bric-à-brac/poxvirus and Zinc finger (BTB/POZ) domain protein, although LDPS does not contain a BTB/POZ domain, as assessed by the InterPro database (Paysan-Lafosse et al. 2023) and previous work (Gingerich et al. 2005). Hence, LDPS is only grouped with BTB/POZ domain-containing proteins due to overall protein sequence similarity. Further, a previous phylogenetic study revealed that LDPS is plant-specific and present in both seedless and seed-bearing plants (de Vries and Ischebeck 2020). This suggested that LDPS evolved before the development of seed-specific processes and, therefore, might be involved in a function(s) associated with desiccation tolerance. On the contrary, a homolog of LDPS, which is annotated (at TAIR) as 1,8-CINEOLE SYNTHASE (18CS) and similarly lacks a BTB/POZ domain, is present in seed-bearing plants, but not in seedless plants, suggesting that it evolved from LDPS after the divergence of seed and seedless plants (de Vries and Ischebeck 2020). The function of 18CS and its subcellular localization, however, have not been explored to date.

The purpose of this study was to characterize the LDPS family of proteins in plants in order to better understand their role(s) in LD biology. Our results show that LDPS and LDPS-like proteins found in seed and seedless plants target specifically to LDs, while 18CS proteins do not. We show also that Arabidopsis LDPS localizes to LDs, in part, via a conserved, predicted amphipathic α-helix and proline hairpin region, and also contains a separate region that binds to the N-terminal portion of OLEO1. Disruption of *LDPS* and constitutive overexpression of *LDPS* in transgenic Arabidopsis resulted in substantially smaller and larger LDs, respectively, in seeds, and a concomitant decrease or increase in seed oil content. Loss of *LDPS* also prevented the enlargement of LDs observed during postgerminative growth. Further, coexpression of *LDPS* and *OLEO1* in a leaf-based LD–LD fusion assay resulted in LD clustering, but no obvious LD–LD fusion, suggesting that LDPS-dependent changes in LD size are seed-specific or might require seed-specific cofactors. Consistent with a function in seeds, freezing treatments prior to stratification revealed that an increase in LD size in *oleo1* mutant seeds is dependent on LDPS and that both proteins work together to produce and protect LDs from freezing-induced LD–LD fusion in seeds and young seedlings. We discuss these and other findings that define LDPS as a key player in plant LD biology important for regulating LD size and oil content in seeds and also modulation of LD size during postgerminative seedling growth and oil breakdown.

## Results

### LDPS and LDPS-like proteins, but not 18CS homologs, localize specifically to LDs

**Figure 1.**
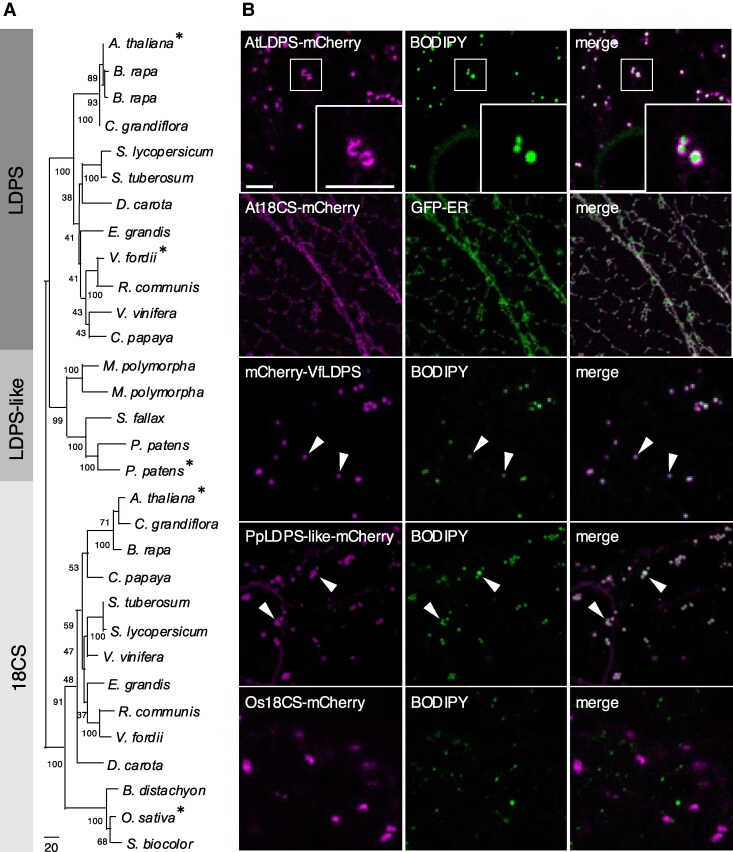
Phylogenetic analysis and intracellular localization of LDPS homologs. **A)** Phylogenetic tree depicting the relationship of selected LDPS, LDPS-like, and 18CS homologs from various plant species, including eudicots, monocots, and seedless plants; refer to Supplementary Fig. S1 for the full phylogenetic tree based on all the LDPS protein homologs currently available at the Phytozome database (Goodstein et al. 2012). Included also in the tree is the LDPS homolog from tung tree (*V. fordii*), based on annotations of the tung tree transcriptome (Cui et al. 2018). Bootstrap values are indicated beside each branch point, and the scale bar represents the number of amino acid substitutions per site. Each protein is labeled with the respective genus and species. Phytozome transcript identifier numbers and sequences for all LDPS protein homologs analyzed in this tree are listed in Supplementary Data Sets 6 and 7. Proteins examined for their intracellular localization in **(B)** are indicated with asterisks. The 3 major clades of proteins were labeled LDPS, LDPS-like, or 18CS. **B)** Representative CLSM images of *N. benthamiana* leaf epidermal cells transiently transformed (as indicated with labels) with mCherry-tagged *Arabidopsis* LDPS (AtLDPS-mCherry), *Arabidopsis* 18CS (At18CS-mCherry), GFP-ER (serving as an ER marker protein), *V. fordii* LDPS (mCherry-VfLDPS), *P. patens* LDPS-like (PpLDPS-like-mCherry), or *O. sativa* 18CS (*Os*18CS-mCherry). LDs were stained with BODIPY. Shown also are the corresponding merged images. The boxes in the images in the top row represent the portion of the cell shown at higher magnification in the insets. Arrowheads indicate examples of mCherry-VfLDPS and PpLDPS-like LDPS localized to BODIPY-stained LDs. Bars = 5 *μ*m and applies to all images and insets in the panel.

To gain insight to the properties of LDPS-type proteins in plants, we first generated a phylogenetic tree of LDPS homologs from the various land plant genomes available at the Phytozome database (Goodstein et al. 2012). As shown in Fig. 1A, which includes selected plant species from the full phylogenetic tree presented in Supplementary Fig. S1, Arabidopsis LDPS and 18CS, along with their homologs from other eudicots and monocots, separated into distinct clades, with a third clade representing homologs from seedless plants (e.g. lycophytes, bryophytes, ferns), similar to the results reported previously (de Vries and Ischebeck 2020). This third clade from seedless plants was more similar to the LDPS group of proteins than to 18CS (Fig. 1A and Supplementary Fig. S1), suggesting that 18CS evolved from LDPS after the origin of angiosperms. Given this closer relationship, the group of proteins from seedless plants was designated as “LDPS-like.”

We next assessed the intracellular localization of several LDPS, LDPS-like, and 18CS proteins. Confocal laser scanning microscopy (CLSM) of *Agrobacterium*-infiltrated *Nicotiana benthamiana* leaf cells revealed that Arabidopsis LDPS fused at its C terminus to the monomeric red fluorescent protein Cherry (AtLDPS-mCherry) localized specifically to LDs stained with the neutral lipid-specific dye boron dipyrromethene (BODIPY) 493/503 (Listenberger et al. 2007) (Fig. 1B). By contrast, mCherry-tagged Arabidopsis 18CS (At18CS-mCherry) did not localize to LDs, but instead targeted to the ER, as evidenced by colocalization with a coexpressed GFP-tagged ER marker protein (GFP-ER; Fig. 1B) (Nelson et al. 2007). The LDPS homolog from the eudicot *Vernicia fordii* (mCherry-VfLDPS), as well as the LDPS-like homolog from the moss *Physcomitrium patens* (PpLDPS-like-mCherry), also localized to LDs (Fig. 1B), whereas the 18CS homolog from the monocot *Oryza sativa* (Os18CS-mCherry) did not localize to LDs, but instead accumulated at distinct puncta, the identity of which was not determined (Fig. 1B). Additional localization experiments revealed that Arabidopsis LDPS with an N-terminal-appended mCherry (mCherry-AtLDPS) also localized specifically to LDs (Supplementary Fig. S2A), indicating that the position of the appended fluorescent protein tag on LDPS did not affect its targeting to LDs in plant cells. We also showed that the BTB/POZ domain-containing protein AT3G50780, which, as mentioned in the Introduction, is one of several BTB/POZ domain-containing proteins in Arabidopsis distantly related to LDPS; refer also to Supplementary Fig. S2B, localized to the cytoplasm and not LDs (Supplementary Fig. S2C). Collectively, these localization studies indicate that targeting to LDs is a unique feature of LDPS and LDPS-like proteins that is not shared by 18CS and other more distantly related homologs in plants.

According to TAIR, the *LDPS* gene is present as a single copy in Arabidopsis. However, there are 2 differentially spliced transcripts: a shorter transcript (*AT3G19920.1*) that has all introns removed and encodes for the protein referred to here as LDPS and the primary focus of this study, and a longer transcript (*AT3G19920.2*), wherein the first intron is not spliced out, resulting in a longer protein almost identical to the shorter form, but with an additional 41-amino-acid-long sequence inserted near its N terminus (Supplementary Fig. S3A). Both transcripts were detected by reverse transcription-polymerase chain reaction (RT-PCR) analysis, consistent with microarray-based expression results (BAR; Winter et al. 2007), and like *OLEO 1 (OLEO1)*, both were restricted primarily to mature (dry) seeds, although some *AT3G19920.2* (and *OLEO1*) transcripts were also detected in imbibed seeds (Supplementary Fig. S3, B and C). Further, despite the differences in primary amino acid sequence near their N termini (Supplementary Fig. S3A), AT3G19920.2-mCherry, like AtLDPS-mCherry (AT3G19920.1), localized specifically to LDs when expressed in plant cells (Supplementary Fig. S3D).

### An internal region of LDPS containing a predicted amphipathic α-helix and proline hairpin motif functions as an LD targeting signal

**Figure 2.**
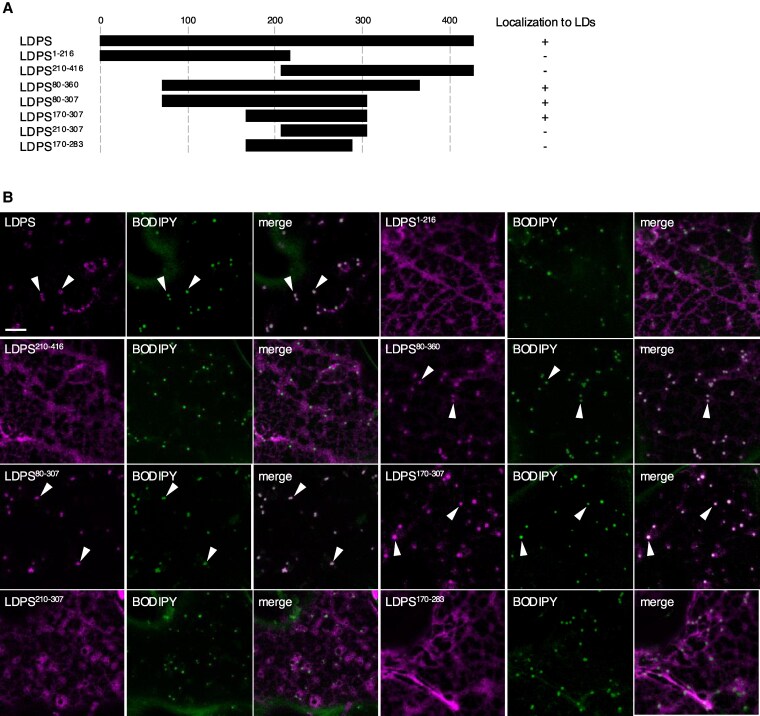
Intracellular localization of various Arabidopsis LDPS truncation mutants in *N. benthamiana* leaf cells. **A)** Schematic representation of full-length LDPS and various LDPS truncation mutants and their corresponding localization to LDs (+) or not (−) in *N. benthamiana* leaf epidermal cells. Refer to **(B)** for representative CLSM images of leaf cells transiently transformed with each construct shown in **(A)** and the corresponding BODIPY-stained LDs. Numbers above the illustration of full-length LDPS represent positions of specific amino acid residues, and the numbers next to the name of each construct denote the amino acids in LDPS that were fused to mCherry; note that the C-terminal-appended mCherry moiety is not depicted in the illustrations or construct names. **B)** Representative CLSM images of *N. benthamiana* leaf epidermal cells transiently transformed (as indicated with labels) with mCherry-tagged **(B)** full-length or truncated versions of Arabidopsis LDPS (refer to illustrations in **A)**. The numbers in the name of each construct denote the amino acids in LDPS that were fused to mCherry; note that the C-terminal-appended mCherry moiety is not included in the construct labels. LDs were stained with BODIPY, and shown also are the corresponding merged images. Arrowheads indicate examples of full-length LDPS and certain mutants (i.e. LDPS^80-360^, LDPS^80-307^, and LDPS^170-307^) that localized to BODIPY-stained LDs. Bars = 5 *μ*m and applies to all images in the panels.

**Figure 3.**
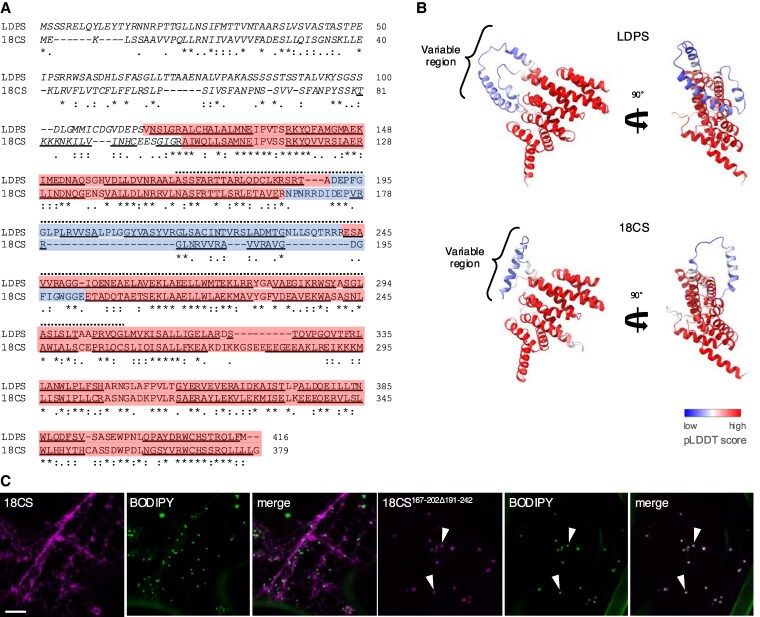
Identification of a discrete internal region in Arabidopsis LDPS that is required for LD targeting. **A)** Deduced polypeptide sequence alignment of Arabidopsis LDPS and 18CS. Identical and similar amino acid residues are indicated with asterisks and colons or periods, respectively. Numbers to the right of each row of sequences represent specific amino acids for each protein. The structures of each protein were predicted using AlphaFold (refer to **B)**. Sequences corresponding to internal “variable” regions of each protein (i.e. amino acids 191 to 242 in LDPS and 167 to 202 in 18CS), whose structures were predicted with lower confidence by AlphaFold, are highlighted in blue, while sequences corresponding to the high-confidence portion of the AlphaFold structure are shown in red; refer also to internal “variable” regions of other selected LDPS protein homologs in Supplementary Fig. S5. Sequences predicted by AlphaFold to form α-helices in each protein are underlined and the N-terminal sequences in both proteins (residues 1 to 114 in LDPS and 1 to 96 in 18CS) that are not shown in the AlphaFold-derived 3D models presented in **(B)** are italicized and not highlighted; the 3D structures of these N-terminal regions were not predicted with high confidence. Also, the sequence corresponding to amino acids 170 to 307 in LDPS, which is the minimally sufficient region for LD localization (refer to Fig. 2), and the corresponding sequence in 18CS (i.e. amino acids 150 to 258) are indicated with stippled lines above. **B)** 3D structures of a portion of Arabidopsis LDPS and 18CS, as predicted by AlphaFold. Amino acids in both proteins are colored based on their AlphaFold pLDDT score, with blue representing low model quality and red representing high model quality, as indicated in the key. The RMSD of differences in atomic positions between 219 pruned atom pairs of LDPS and 18CS proteins calculated by ChimeraX Matchmaker (Pettersen et al. 2021) is 0.885 Å. As indicated in **(A)**, the low-confidence-structure, N-terminal regions of LDPS and 18CS (residues 1 to 114 and 1 to 96, respectively) were removed for visualization. Note also that the LD targeting information of LDPS is located within a polypeptide sequence that includes the variable blue region. Refer to Supplementary Fig. S6 for additional examples of the AlphaFold-based structures of LDPS, LDPS-like, and 18CS proteins (and their corresponding RMSD values compared to Arabidopsis LDPS) from selected plant species. **C)** Representative CLSM images of *N. benthamiana* leaf epidermal cells transiently transformed with mCherry-tagged Arabidopsis (full-length) 18CS and the mutant 18CS^167-202Δ191-242^, consisting of the internal “variable” region in 18CS (amino acid residues 167 to 202) replaced with the internal “variable” region in Arabidopsis LDPS (amino acid residues 191 to 242). The C-terminal-appended mCherry moiety is not included in the construct labels. LDs were stained with BODIPY and shown also are the corresponding merged images. Arrowheads indicate examples of colocalization of 18CS^167-202Δ191-242^-mCherry and BODIPY-stained LDs. Bar = 5 *μ*m and applies to all images in the panel.

We next sought to identify the region(s) in LDPS responsible for its targeting to LDs in plant cells. Toward that end, we constructed several truncation mutants of Arabidopsis LDPS that were fused to mCherry and then assessed for their ability to localize to LDs in plant cells. As summarized in Fig. 2A (see also Fig. 2B for representative micrographs), neither the N- or C-terminal halves of LDPS (i.e. LDPS^1-216^-mCherry and LDPS^210-416^-mCherry) were sufficient for targeting to LDs. However, several mutant versions of LDPS consisting of partially overlapping, internal regions of the protein did localize to LDs. Among these, the region corresponding to amino acids 170 to 307 in LDPS (LDPS^170-307^-mCherry) was found to be minimally sufficient for LD localization (Fig. 2, A and B). By contrast, a similar internal region in Arabidopsis 18CS, i.e. amino acid residues 150 to 258 (refer to polypeptide sequence alignment in Fig. 3A, which is discussed below), was not sufficient for targeting to LDs (Supplementary Fig. S4), as expected, since the full-length 18CS protein was localized specifically to the ER (Fig. 1B).

To better define the LD targeting signal in Arabidopsis LDPS, we compared polypeptide sequence alignments between LDPS- and 18CS-type proteins. As shown in Fig. 3A, Arabidopsis LDPS and 18CS share moderate sequence identity/similarity overall, but there was significant variability in both amino acid content and length within the internal region containing the LD targeting signal of LDPS, i.e. amino acid residues 170 to 307. Similar trends were observed when comparing the polypeptide sequences of other phylogenetically diverse homologs of LDPS, LDPS-like and 18CS proteins (Supplementary Fig. S5). The conservation of polypeptide sequence characteristics among members of the same protein families suggested there might be structural differences between the protein families that contribute to differences in their functionality and/or intracellular localization. Indeed, analysis of protein family members using AlphaFold (Jumper et al. 2021 ; Varadi et al. 2022) indicated that Arabidopsis LDPS and 18CS, along with all other homologs from the other representative plant species examined, had similar overall predicted tertiary structures, which included a single globular domain of several conserved α-helices (highlighted red in Fig. 3, A and B and Supplementary Fig. S6) and a largely unstructured N-terminal region (italicized in Fig. 3A, but not included in Fig. 3B due to its poor prediction scores). Within the globular domain, a region of greater sequence and structural variability is found (colored blue in Fig. 3, A and B and Supplementary Fig. S6). This sequence, representing amino acids 191 to 242 of LDPS, is located within the shortest region of LDPS shown to be sufficient for localization to LDs (amino acids 170 to 307; Fig. 2). To test whether this internal “variable” region served as an LD targeting signal in LDPS, we replaced the corresponding variable region of Arabidopsis 18CS (i.e. residues 167 to 202; Fig. 3, A and B) with that from Arabidopsis LDPS and observed that the resulting hybrid protein (i.e. 18CS^167-202D191-242^-mCherry), localized specifically to LDs (Fig. 3C).

**Figure 4.**
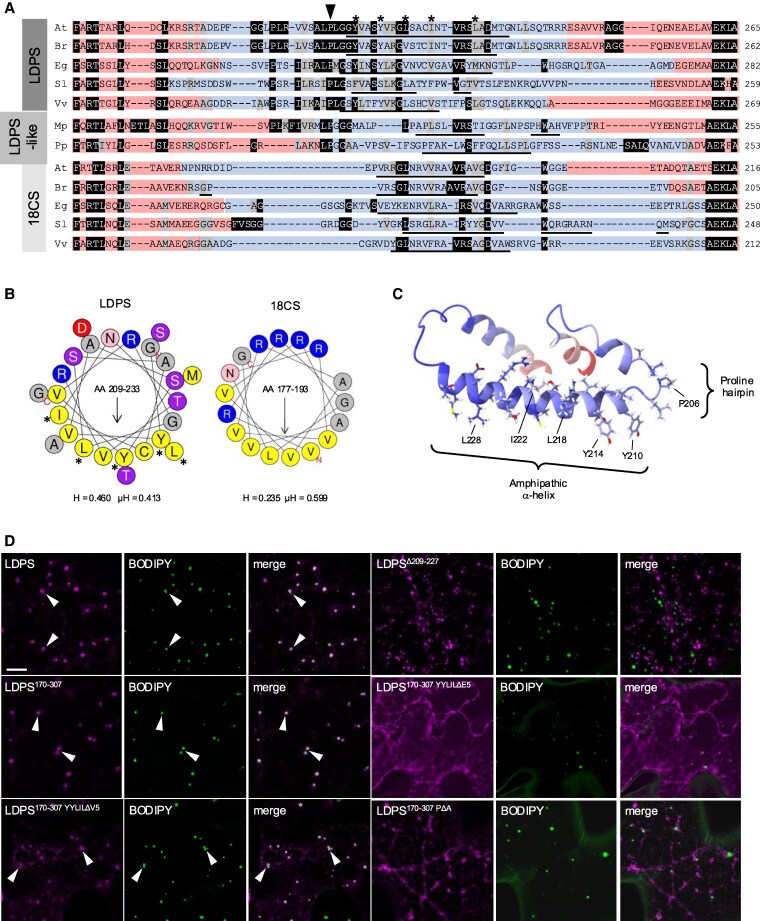
A predicted amphipathic α-helix and proline hairpin sequence in Arabidopsis LDPS function as an LD targeting signal. **A)** Alignment and Boxshade analysis of deduced polypeptide sequences of the internal “variable” regions in LDPS, LDPS-like, and 18CS proteins from selected plant species. Proteins are labeled with the abbreviation for their respective genus and species (e.g. At, *Arabidopsis thaliana*; Br, *Brassica rapa*, etc.), and correspond to those shown in the phylogenetic tree in Fig. 1A; refer also to the full-length polypeptide sequence alignment in Supplementary Fig. S5 and the AlphaFold protein structures presented in Supplementary Fig. S6. Amino acids that are identical or similar in 50% or more of the aligned sequences are indicated with black and gray shading, respectively. Numbers to the right of each row of sequences represent specific amino acids for each protein. The sequences corresponding to the internal “variable” regions of each protein (e.g. amino acids 191 to 242 in Arabidopsis LDPS) and those predicted with higher confidence by AlphaFold (refer to Fig. 3B and Supplementary Fig. S6), are highlighted in blue and red, respectively. The sequences in the variable region of each protein that are predicted by AlphaFold to form an amphipathic α-helix are underlined. The conserved proline at position 206 (P206) in Arabidopsis LDPS and the large hydrophobic residues in the predicted amphipathic α-helix in Arabidopsis LDPS (i.e. Y210, Y214, L218, I222, and L228) are indicated above the sequences with an arrowhead and asterisks, respectively; refer also to the helical wheel projection of the amphipathic α-helix in Arabidopsis LDPS in **(B)**, as well as the three-dimensional model of the internal variable region of Arabidopsis LDPS shown in **(C)**. **B)** Helical wheel projections of the predicted amphipathic α-helix in the “variable” region in Arabidopsis LDPS and 18CS. Shown are the α-helical wheel projections (based on HeliQuest) of the sequences in the “variable” regions of Arabidopsis LDPS (residues 209 to 233) and 18CS (residues 177 to 193). Hydrophobic amino acid residues are colored yellow, small-sized residues are gray, polar residues are pink or purple, and charged residues are red or blue. Asterisks denote the 5 conserved, large hydrophobic residues in the predicted α-helix in Arabidopsis LDPS (i.e. Y210, Y214, L218, I222, and L228) that were mutated to glutamic acids or valines; refer to the localization of the LDPS^170-307^-based mutant constructs shown in **(D)**, as well as **(A)** and **(C)**. Note the overall enrichment of large hydrophobic residues (i.e. I, L, Y, F, and M) on one side of the α-helical wheel for LDPS compared with 18CS, and the similar differences in the distribution of large hydrophobic residues in the α-helical wheel projections for selected LDPS and LDPS-like proteins compared with those for 18CS proteins in Supplementary Fig. S7. The arrow within each helical wheel projection corresponds to the direction of the hydrophobic moment, and the numbers shown represent the specific amino acids corresponding to the predicted α-helix in each protein, as depicted (underlined) also in **(A)**. Shown also for both helical wheels are the corresponding hydrophobicity (H) and hydrophobic moment (μH) scores, based on HeliQuest; note the relatively higher hydrophobicity score for LDPS compared with 18CS and, likewise, for selected LDPS and LDPS-like proteins compared with 18CS proteins in Supplementary Fig. S7. **C)** 3D protein structure of the internal “variable” region in Arabidopsis LDPS (residues 191 to 242), as predicted by AlphaFold. The conserved proline at position 206 (P206) in the predicted proline hairpin structure and the 5 large hydrophobic residues located on the same face of the predicted amphipathic α-helix (i.e. Y210, Y214, L218, I222, and L228) are indicated. Note that the proline residue in the predicted proline hairpin structure in Arabidopsis LDPS is conserved in other LDPS and LDPS-like proteins (see **[A]**). **D)** Representative CLSM images of *N. benthamiana* leaf epidermal cells transiently transformed with mCherry-tagged full-length or truncated/mutated versions of Arabidopsis LDPS, including LDPS^Δ209-227^, which lacks residues 209 to 227, LDPS^170-307^ (consisting of residues 170 to 307 in LDPS, which is minimally sufficient for targeting to LDs; see Fig. 2) or LDPS^170-307^ with the large hydrophobic residues in the predicted amphipathic sequence replaced with either valines (LDPS^170-307YYLILΔV5^) or glutamic acids (LDPS^170-307YYLILΔE5^), or the conserved proline (P206) replace with an alanine (LDPS^170-307PΔA^); refer also to **(A)** to **(C)**. Note that the C-terminal-appended mCherry moiety is not included in the construct labels. LDs were stained with BODIPY, and shown also are the corresponding merged images. Arrowheads denote examples of protein localization to BODIPY-stained LDs. Bar = 5 *μ*m and applies to all images in the panel.

A closer analysis of the characteristics within the variable region of LDPS, LDPS-like, and 18CS-type proteins (Fig. 4A and Supplementary Figs. S5 and S6) revealed that while all groups of proteins possess at least 1 predicted amphipathic α-helix in this region, the α-helices in the LDPS and LDPS-like family of proteins were more hydrophobic overall and had a hydrophobic face more enriched in large hydrophobic residues (i.e. W, F, Y, L, I, or M) (Fig. 4B and Supplementary Fig. S7), which are known to be important for association with LDs (Dhiman et al. 2020; Olarte et al. 2022). LDPS and LDPS-like proteins also contained a conserved proline that is not found in 18CS proteins (Fig. 4A) and is part of a -LPLG- predicted hairpin/turn-like structure immediately preceding the amphipathic α-helix (Fig. 4C and Supplementary Fig. S5). Given that amphipathic α-helices and proline hairpin motifs are both known to serve as LD targeting signals (Dhiman et al. 2020; Olarte et al. 2022), we tested whether these elements might be also important for targeting Arabidopsis LDPS to LDs. As shown in Fig. 4D, deletion of amino acids 209 to 227 from LDPS, which removes most of the predicted amphipathic α-helix sequence within the LDPS internal variable region (i.e. residues 209 to 233; Fig. 4A), resulted in a mutant protein, LDPS^Δ209-227^-mCherry, that, unlike full-length LDPS, did not localize to LDs, but instead localized to unknown puncta. Similarly, LDPS^170-307^, the minimally sufficient region that targeted to LDs (Figs. 2 and 4D), mislocalized to the cytoplasm when several of the large hydrophobic residues along the hydrophobic face of the amphipathic helix (Fig. 4, B and C) were replaced with hydrophilic glutamic acids (LDPS^170-307YYLILΔE5^-mCherry), which presumably disrupted the amphipathic and overall hydrophobic nature of the helix. However, when the same large hydrophobic residues in LDPS^170-307^ were replaced with smaller hydrophobic valine residues, the resulting mutant protein (i.e. LDPS^170-307YYLILDV5^-mCherry) still localized to LDs (Fig. 4D), although some mistargeting was also observed. Replacement of the conserved proline residue in LDPS^170-307^ with an alanine (i.e. LDPS^170-307PΔA^-mCherry) also disrupted targeting to LDs (Fig. 4D). These results indicate that the predicted amphipathic α-helix within the internal variable region of Arabidopsis LDPS is necessary for LD targeting and can accommodate smaller hydrophobic residues along the hydrophobic face. Further, the proline residue just upstream of the amphipathic α-helix is critically important for LD targeting, perhaps by presenting the amphipathic α-helix in the proper orientation to facilitate LD association and/or contributing directly to LD targeting by binding of the proline hairpin to LDs directly.

### Arabidopsis *ldps* mutants have smaller LDs in mature seeds and young seedlings and store less seed oil

Given that LDPS in Arabidopsis is expressed exclusively in seeds and seedlings and localizes specifically to LDs, we investigated whether disruption of *LDPS* gene expression affects LD biology during seed development, germination, and/or early seedling growth. Toward that end, 2 independent Arabidopsis *LDPS* mutant lines were examined: *ldps-1*, which contains a T-DNA insertion in the second exon of the *LDPS* gene in the Nossen-0 (Nos-0) background, and *ldps-2*, which was generated using Clustered Regularly Interspaced Short Palindromic Repeats (CRISPR)/CRISPR-associated protein 9 (Cas9)-based genome editing to remove an internal sequence encoding 135 (of 416) amino acids in the LDPS polypeptide in the Columbia-0 (Col-0) background (Supplementary Fig. S8, A and B). Both mutant lines were confirmed via progeny analysis and genotyping, as well as RT-PCR analysis, for homozygosity of the mutated *LDPS* gene and disruption in the expression of full-length *LDPS* transcripts, respectively (Supplementary Fig. S8C).

**Figure 5.**
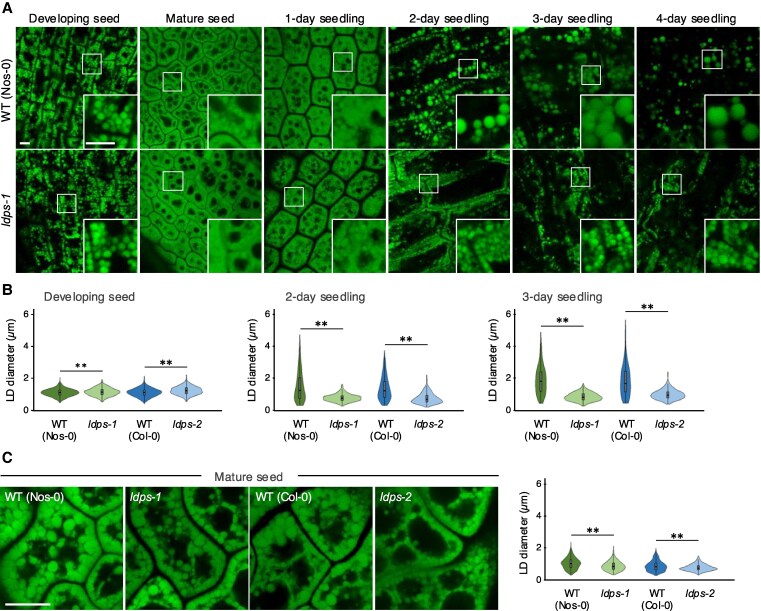
Disruption of LDPS expression influences LD size in Arabidopsis seeds and seedlings. **A)** Representative CLSM images of BODIPY-stained LDs in hypocotyl or cotyledon cells from WT (Nos-0) and *ldps-1* embryos at various developmental stages, including (as indicated with labels) developing seeds from siliques at 10 to 12 d after flowering, mature (dry) seeds, and seedlings 1, 2, 3, and 4 d after the initiation of germination. Boxes in images represent the portion of the cells shown at higher magnification in the insets. Refer to Supplementary Fig. S9A for the corresponding CLSM images of WT (Col-0) and *ldps-2* embryos at the same developmental stages. Bars = 5 *μ*m and applies to all images and insets in the panel. **B)** Quantification of LD sizes in WT and *ldps* embryos in developing seeds and seedlings 2 and 3 d after the initiation of germination. Diameters of BODIPY-stained LDs were measured using ImageJ (refer to “Materials and methods” for details) and values shown in violin plots represent those obtained from 3 biological replicates, with each replicate consisting of 6 to 8 seed or seedling samples per plant line and 2 micrographs per sample, including those shown in **(A)** for WT (Nos-0) and *ldps-1* and in Supplementary Fig. S9A for WT (Col-0) and *ldps-2*. Single and double asterisks represent statistically significant differences at *P* ≤ 0.05 and *P* ≤ 0.01 related to the corresponding WT and *ldps* plant lines, respectively, as determined by a two-tailed Student's *t* test. **C)** LD sizes in embryos of WT and *ldps* mature seeds based on imaging with an Airyscan CLSM. Shown on the left are representative images of BODIPY-stained LDs in cotyledon cells from (as indicated with labels) WT (Nos-0 and Col-0) and corresponding *ldps-1* and *ldps-2* embryos in mature seeds. Images shown were obtained using high-resolution Airyscan CLSM (rather than regular CLSM, as in **(A)** and for all other micrographs shown in this study) to better distinguish closely appressed, individual LDs in cells in mature seeds. Bar = 10 *μ*m and applies to all images in the panel. Quantifications of LD diameters are shown in the violin plots on the right. LD diameters were measured using ImageJ and values shown represent those obtained from a data set of 10 micrographs per plant line, including those shown in the panel. Statistically significant differences of at least *P* ≤ 0.05, as denoted by asterisks, were determined by a two-tailed Student's *t* test. A summary of the statistical analysis for **(B)** and **(C)** is given in Supplementary Table S4.

CLSM imaging of BODIPY-stained LDs was used to assess potential differences in LD morphology in wild-type (WT) and *ldps* mutant plants. As shown in Fig. 5, A and B and Supplementary Fig. S9A, in developing seed embryos from siliques at 10 to 12 d after flowering, which corresponds to the “bent” stage of seed development in Arabidopsis (Le et al. 2010), LDs were just slightly larger in size in the *ldps-1* and *ldps-2* mutant lines in comparison with their WT controls. By contrast, LDs in mature (dry) seeds were slightly smaller in both *ldps* mutants compared with WT (Fig. 5C), indicating that LDPS has a minor, but detectable role in influencing LD morphology during seed development and maturation. Notably, expression of *LDPS* is low during developmental stages and highest in mature seeds and at the onset of seed germination (Kretzschmar et al. 2020; Supplementary Fig. S3). During postgerminative seedling growth, LDs in WT enlarged considerably 2 d after the initiation of germination (Fig. 5, A and B and Supplementary Fig. S9A), as expected and presumably due to LD–LD fusion associated with OLEO protein breakdown and the increase in lipolysis (Miquel et al. 2014; Deruyffelaere et al. 2018; Kretzschmar et al. 2018). In comparison, LDs in seedlings from both *ldps* mutant lines were strikingly smaller at 2 d after germination than in WT controls and remained small throughout early postgerminative growth (Fig. 5, A and B and Supplementary Fig. S9A). By contrast, examination of LDs in tissues where LDPS is not expressed, such as 15-d-old leaf cells and pollen grains from l*dps-2* mutant plants, showed no obvious effects on LD morphologies in comparison with WT (Supplementary Fig. S9, B and C), although there was a slight, but statistically significant increase in LD size in *ldps-2* mutant leaves (Supplementary Fig. S9B). Collectively, these results indicate that LDPS plays a primary role in determining LD size in seeds and seedlings in Arabidopsis and perhaps does so by influencing the process of LD–LD fusion.

To confirm that loss of *LDPS* function was responsible for the smaller LD phenotype observed in the *ldps* mutant plants, we generated a plant line that constitutively overexpressed *LDPS* in the *ldps-2* background, as confirmed by RT-PCR analysis (Supplementary Fig. S10A). Overall, the LDs in seeds and young seedlings in this plant line (i.e. *LDPS OE ldps-2*) resembled more the typical distribution of LD sizes observed in WT and lacked the smaller LDs characteristic of the *ldps-2* mutant (Supplementary Fig. S10B). However, several prominent “supersized” LDs were also observed in *LDPS OE ldps-2* seeds and young seedlings (Supplementary Fig. S10B), which we interpret to result from overexpression of the *LDPS* coding sequence. We also showed that, unlike *ldps* mutants, *18CS*-disrupted plants (*18cs*) did not display any obvious LD phenotype in seedlings (Supplementary Fig. S11), consistent with 18CS not being an LD protein (Fig. 1B) nor having any known LD-related function(s).

**Figure 6.**
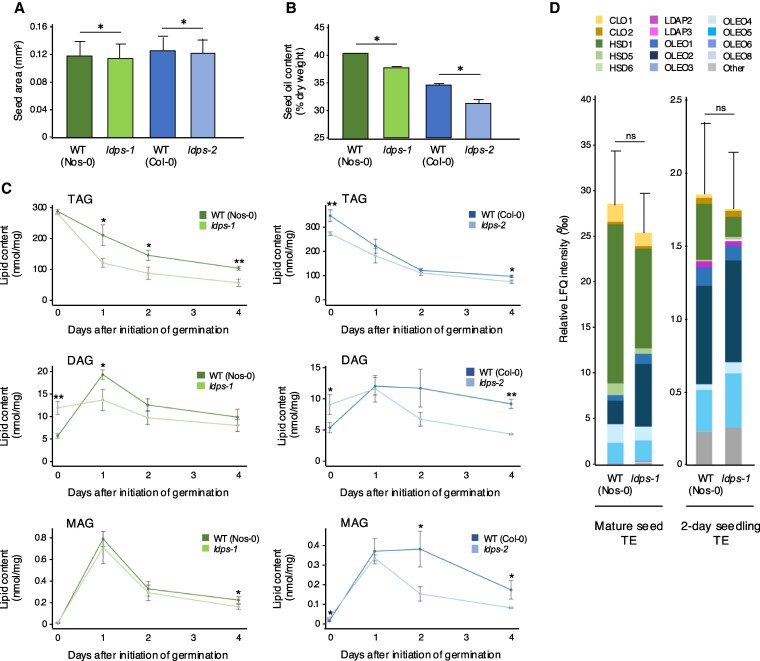
LDPS influences Arabidopsis seed size, seed oil content, and the proportion of DAG in seeds, but is not involved in bulk LD coat protein turnover during postgerminative growth. **A)** Comparison of WT and *ldps* seed size. Mature (dry) seeds of each plant line (as indicated) were imaged using a document scanner, and the area of individual seeds was measured using ImageJ. Values represent the mean ± SD from 400 to 600 seeds from 8 plants for each plant line, and asterisks represent statistically significant differences (*P* ≤ 0.01) between *ldps* mutant plant lines and their respective WT controls, as determined by a two-tailed Student's *t* test. **B)** Comparison of WT and *ldps* seed oil content. Oil content as a percentage of dry weight in mature seeds from each plant line (as indicated) was determined using NMR. Values shown represent the means ± SD of 3 replicates of 50 mg of seeds for each line. Asterisks indicate statistically significant differences (*P* ≤ 0.05) between *ldps* mutant plant lines and their respective WT controls, as determined by a two-tailed Student's *t* test. **C)** Content of TAG, DAG, and MAG in WT and corresponding *ldps* mutant seeds and seedlings. Total lipids were extracted from mature seeds (indicated as 0 d) and seedlings harvested at 1, 2, and 4 d after the initiation of germination and analyzed by LC-MS/MS; refer to “Materials and methods” for additional details. Values represent the mean ± SD of the sum of individual lipid molecular species (in nmol/mg dry weight) for, as indicated with labels, TAGs, DAGs, and MAGs, identified from analysis of 4 biological replicates. The molecular species of each lipid class (Mol percentage) are shown in Supplementary Figs. S12 to S14. Single and double asterisks represent statistically significant differences at *P* ≤ 0.05 and *P* ≤ 0.01 related to the corresponding WT and *ldps* plant lines, respectively, as determined by a two-tailed Student's *t* test. **D)** Total abundance of LD coat proteins in proteomes derived from total protein extracts (TE) of WT (Nos-0) and *ldps-1* seeds and seedlings. Proteins were isolated from total homogenates (and from corresponding LD-enriched fractions; see Supplementary Fig. S16A) of WT and *ldps-1* mature (rehydrated) seeds and seedlings at 2 d after the initiation of germination. Total label-free quantification (LFQ) intensities of all proteins were summed from the LC-MS/MS data; refer to “Materials and methods” for details and Supplementary Data Sets 1 to 5 for the values and enrichment ratios for all proteins identified in all samples. All proteomics data are available also through the ProteomeXchange Consortium via the PRIDE partner repository (accession no. PXD041506); refer to Supplementary Table S3. Values shown in bar graphs are the mean ± SD per mille of total LFQ intensities for known Arabidopsis LD coat proteins, based on Ischebeck et al. (2020), in the TE (and in corresponding LD-enriched factions; refer to Supplementary Fig. S16A) from 5 biological replicates (i.e. 5 separate total protein extractions and LD isolations per plant line); refer to the PCA plot of the TE and LD-enriched protein groups from the 5 replicates of each stage presented in Supplementary Fig. S16B. Each class of LD coat proteins, including CLOs, HSDs, LDAPs, and OLEOs, as well as other LD proteins that were plotted together (referred to as “Other”), is colored according to the key. No statistically significant differences were found in the total abundance of LD coat proteins in the TE of WT and *ldps-1* seeds and 2-day-old seedlings, based on two-tail Student's *t* test (i.e. *P ≥* 0.05); ns, not significant. CLO, caleosin; HSD, hydroxysteroid dehydrogenase (steroleosin); LDAP, LD-associated protein; OLEO, oleosin. Refer also to Supplementary Data Set 1 for additional results for individual protein abundance, including known LD coat proteins, summed from LC-MS/MS data. A summary of the statistical analysis for **(A)** to **(D)**, as well as the allied results presented Supplementary Fig. S16, is given in Supplementary Table S4.

Based on the observations that LDPS influences LD size in seeds and seedlings, we examined whether disruption of *LDPS* affected other seed-related traits, including seed size and seed oil content and composition. As shown in Fig. 6, A and B, mature seeds of both *ldps-1* and *ldps-2* mutants were significantly smaller in size and, based on nondestructive ^1^H-nuclear magnetic resonance (NMR) assays, contained lower oil content in comparison with their corresponding WT controls. Since the latter provide an overall estimate of seed oil content but also detect other lipophilic compounds in seeds besides TAGs, a more detailed lipidomics analysis of total lipid extracts from Arabidopsis WT and *ldps* mutant seeds and seedlings was conducted. As shown in Fig. 6C, liquid chromatography–tandem MS (LC-MS/MS) quantification of lipid classes (summed from individual molecular species; see Supplementary Figs. S12 to S14) confirmed that TAG content in both *ldps* mutants was generally lower in mature seeds and remained lower during postgerminative seedling growth in comparison with WT. However, the overall rates of TAG degradation in both *ldps* mutants and WT seedlings were generally similar (Fig. 6C). By contrast, diacylglycerol (DAG) content was significantly higher in mature seeds of both mutant lines in comparison with WT, but then decreased in comparison with WT during postgerminative seedling growth (Fig. 6C). Monoacylglycerol (MAG) content in mutant seedlings was also lower than the respective WT lines during postgerminative growth, but was more similar to WT in mature seeds (Fig. 6C). Examination of individual lipid molecular species within each lipid class, at each time point, showed no major differences in composition of TAG, DAG, or MAG in both *ldps* mutant lines and WT, although the DAG pool derived from mature seeds showed a slight enrichment in the relative proportions of 18:2 to 18:3 and 18:3 to 20:2 DAGs in both mutants (Supplementary Figs. S12 to S14). There were also no significant differences in content or composition of polar lipids (i.e. phospholipids and lysophospholipids) in mature or germinated seedlings (Supplementary Fig. S15). Collectively, these results suggest that loss of *LDPS* function primarily affects bulk lipid content rather than changing relative proportions of individual lipid molecular species, although DAG molecular species were notably altered in seeds and seedlings of *ldps* mutant plants.

The smaller LD phenotype in *ldps* mutants during postgerminative growth is reminiscent of the phenotype observed in Arabidopsis *pux10* mutants (Deruyffelaere et al. 2018; Kretzschmar et al. 2018; refer also to Supplementary Fig. S11C). PUX10 is an LD coat protein required for the degradation of other LD proteins, including OLEOs, during postgerminative seedling growth. Specifically, the loss of *PUX10* results in a near doubling of OLEO protein amounts in seedlings at 2 d after germination due to a decrease in the rates of protein turnover, which is thought to inhibit LD–LD fusion, resulting in the observed smaller LD phenotype (Deruyffelaere et al. 2018; Kretzschmar et al. 2018 ). To determine whether LDPS might also be involved in regulating turnover of LD proteins during postgerminative growth, we conducted a proteomics analysis of Arabidopsis WT and *ldps-1* mature seeds and 2-d-old seedlings. As shown in Fig. 6D, comparison of total amounts of known LD coat proteins was not significantly different in total protein extracts from seeds and 2-d-old seedlings of WT and *ldps-1* mutant plants. Similarly, the overall abundance of LD proteins was not significantly different in the corresponding LD-enriched fractions isolated from WT and *ldps-1* mature seeds and 2-d-old seedlings (Supplementary Fig. S16A; refer also to Supplementary Fig. S16B for the principal component analysis [PCA] plot of the total protein extracts and LD-enriched factions from the 5 replicates of each stage). These observations indicate that the underlying cause of the small LD phenotype in the *ldps* mutant seedlings is distinct from the one responsible for the small-sized LDs observed in Arabidopsis *pux10* mutants. On the contrary, there were significant differences in the abundance of at least a few of the LD coat proteins in total protein extracts or LD-enriched fractions from WT and *ldps-1* mature seeds, including a decrease in the steroleosins HSD1 and HSD5 and CLO2, as well as an increase in LDAP2 and some OLEOs, which might contribute to the smaller LD size in *ldps* mutant seeds (refer to Supplementary Data Set 1 for a summary of the amounts of individual LD coat proteins in WT and *ldps-1* seeds; refer also to Supplementary Data Sets 2 to 5 for additional lists of proteins detected by LC-MS/MS). However, these compositional differences did not persist in WT and *ldps-1* 2-d-old seedlings, in neither total extracts nor LD-enriched fractions (Supplementary Data Set 1), indicating that the small LD phenotype in *ldps* mutant seedlings was likely not due to the compositional differences observed in seeds. Whether there are other minor protein constituents that were different in *ldps* mutant plants in comparison with WT that contributed to the smaller LD phenotype in seeds and seedlings is an open question.

Other than the smaller seed size, less seed oil content, and smaller LD morphology, we did not observe any other obvious growth or developmental differences between WT and *ldps* mutant plants. For instance, the *ldps* mutant seeds germinated at similar rates (Supplementary Fig. S17A), displayed no differences in postgerminative growth rates based on either hypocotyl elongation assays with dark-grown seedlings or whole seedlings grown in the light (Supplementary Fig. S17, B and C), and plants grown in soil reached a similar height at maturity (Supplementary Fig. S17D). WT and *ldps* mutant plants also produced similar numbers of siliques, and their seed yield was not significantly different (Supplementary Fig. S17, E and F). This again is likely a reflection of low *LDPS* expression in nonseed tissues (Supplementary Fig. S3).

### LDPS interacts with OLEO in yeast and plant cells

**Figure 7.**
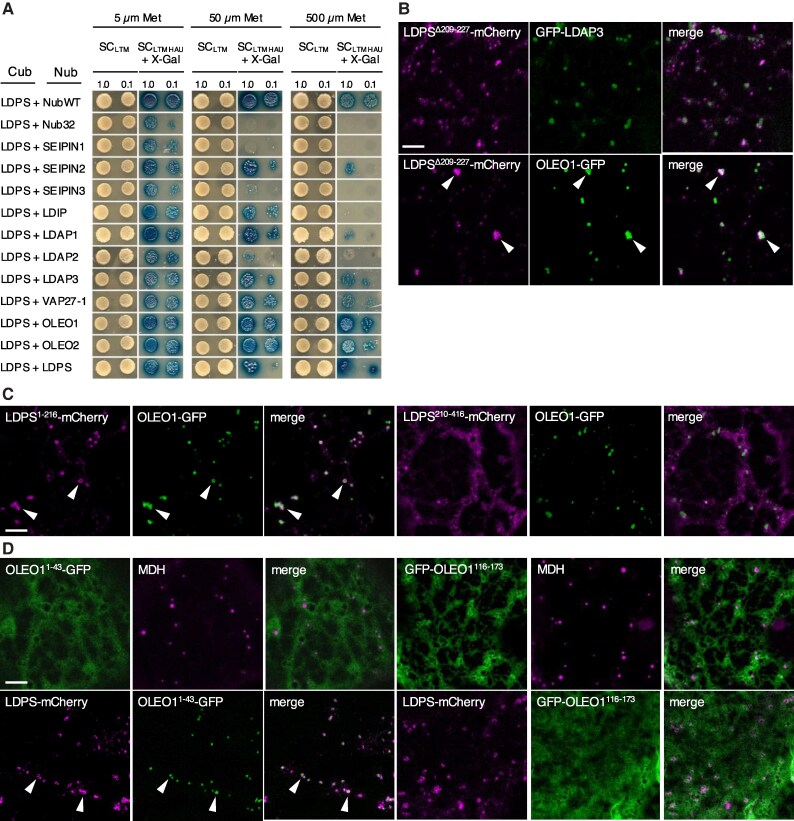
Characterization of LDPS protein–protein interactions and identification of regions within LDPS and OLEO1 required for their colocalization at LDs. **A)** mbSUS analysis of LDPS and various known Arabidopsis LD biogenetic proteins in yeast. LDPS-Cub, serving as “bait” and consisting of full-length Arabidopsis LDPS fused to the C-terminal half of ubiquitin (Cub), as well as the transcriptional reporter protein complex ProteinA-LexA-VP1, was cotransformed into yeast (*Saccharomyces cerevisiae*) cells along with various “prey” proteins, including: NubWT, consisting of the native sequence for the N-terminal half of ubiquitin (Nub), which has high affinity for Cub and serves as a positive control with the “bait” (i.e. LDPS-Cub); Nub32, which contains a point mutation in the Nub sequence that results in low affinity for Cub and serves as a negative control with LDPS-Cub; or Nub32 fused to various full-length Arabidopsis LD biogenetic proteins, as indicated. Refer to Supplementary Fig. S18 for western blot analysis of total proteins extracted from each yeast strain, confirming expression of each Nub and Cub construct. Yeast cells were plated as serial dilutions of 1.0 or 0.1 OD_600_ on both low- and high-stringency conditions (i.e. synthetic complete [SC]-_LWM_ and SC_-LWMHAU_), the latter of which requires protein–protein interactions for yeast growth. Both sets of plates also contained 5, 50, or 500 *μ*M methionine (Met), which allows for control of LDPS-Cub expression via its Met-repressible promoter. Selection plates also included X-Gal (SC_-LWMHAU_ + X-Gal), which provided an additional qualitative measure of protein–protein interaction; refer to Materials and methods for additional details on mbSUS assays. Plasmid combinations are shown to the left, and the images of the corresponding serial dilutions are shown on the right. Note the appearance of growth (and blue coloration) on higher selection media of yeast expressing LDPS-Cub and NubWT, but no growth when LDPS-Cub was coexpressed with Nub32, indicating the lack of autoactivation. The results are representative of at least 5 separate cotransformations of yeast with each plasmid combination. Representative CLSM images of *N. benthamiana* leaf epidermal cells transiently (co)transformed with (as indicated with labels) **B)** LDPS^Δ209-227^-mCherry and GFP-tagged Arabidopsis LDAP3 (GFP-LDAP3) or OLEO1 (OLEO1-GFP), **C)** LDPS^1-216^-mCherry or LDPS^210-416^-mCherry with OLEO1-GFP, or **D)** OLEO1^1-43^-GFP or GFP-OLEO1^116-173^ without or with LDPS-mCherry; refer to topology model of Arabidopsis OLEO1 in Supplementary Fig. S19C. Shown also are the corresponding merged images. In **(D)**, LDs were stained with MDH, and arrowheads in **(B)** to **(D)** indicate examples of protein colocalization at LDs. Note that OLEO1-GFP expressed on its own localizes to LDs, as expected (refer to Supplementary Fig. S19B), and that the localization of LDAP3 to LDs in *N. benthamiana* cells has been reported elsewhere (Pyc et al. 2021). Bars in **(B)** to **(D)** = 5 *μ*m and applies to all images in the panels.

The formation of LDs in plant cells is orchestrated by a suite of proteins, some of which are known to physically interact (reviewed in Guzha et al. 2023). Consequently, we investigated whether LDPS interacts with any known plant LD biogenetic proteins. As shown in Fig. 7A, no interaction was observed between LDPS and Arabidopsis SEIPIN1, SEIPIN3, nor LDAP2, compared with the negative control (i.e. LDPS and Nub32) when measured using a mating-based yeast split–ubiquitin system (mbSUS) assay (Grefen et al. 2009) (Fig. 7A and Supplementary Fig. S18). However, LDPS showed positive interactions with Arabidopsis SEIPIN2, LDIP, LDAP1, LDAP3, VAP27-1, OLEO1, and OLEO isoform 2 (OLEO2), as well as with itself (i.e. LDPS self-association), and while the strengths of these positive interactions varied, those between LDPS and OLEO1 or OLEO2 appeared to be the strongest (Fig. 7A).

To confirm and extend these findings, we next examined the potential interaction between LDPS and selected LD biogenetic proteins in plant cells. In doing so, we took advantage of previous experiments showing that ectopically expressed LDIP is localized primarily to LDs in *N. benthamiana* leaf cells, but when coexpressed with ER-localized SEIPIN, LDIP relocalized to the ER, which provided supportive evidence for the physical interaction between these 2 proteins during LD biogenesis (Pyc et al. 2021). However, when we used this protein relocalization strategy to test whether LD-localized LDPS might be relocalized to the ER when coexpressed with ER-localized SEIPIN2 or VAP27-1, the localization of LDPS was unaffected by coexpression with either protein, i.e. LDPS-mCherry localized to LDs and not to GFP-SEIPIN2- or VAP27-1-GFP-containing ER (Supplementary Fig. S19A). This suggests that, *in planta*, LDPS does not physically interact with SEIPIN2 or VAP27-1, or does so in a manner that is not strong enough to influence LDPS relocalization from LDs to the ER.

We then employed a different protein relocalization strategy to test for potential interactions of LDPS with other LD coat proteins by taking advantage of a mutant version of LDPS, i.e. LDPS^Δ209-227^, which, because of the disruption in the protein's LD targeting signal, does not localize to LDs (Fig. 4D). More specifically, we hypothesized that LDPS^Δ209-227^ might relocalize to LDs when coexpressed with an LD-localized protein-binding partner. As shown in Fig. 7B, LDPS^Δ209-227^-mCherry did not colocalize with coexpressed GFP-LDAP3 at LDs, but did when coexpressed with OLEO1-GFP, which localizes to LDs when expressed on its own (Supplementary Fig. S19B), as expected (Pyc et al. 2021). These data suggest that, *in planta*, LDPS interacts with OLEO1; however, akin to SEIPIN2 and VAP27-1, does not interact with LDAP3 or not strongly enough to allow for protein relocalization. It is also possible that the mutation to LDPS (i.e. LDPS^Δ209-227^) altered the protein structure in a way that reduced its ability to bind to LDAP3 (and SEIPIN2 or VAP27-1).

The interaction between LDPS and OLEO1 was further explored by assessing which region(s) within the 2 proteins are responsible for their association at LDs in plant cells. As shown in Fig. 7C, LDPS^1-216^-mCherry, but not LDPS^210-416^-mCherry, colocalized with coexpressed OLEO1-GFP at LDs, whereas neither of these 2 LDPS regions localized to LDs when expressed on their own (Fig. 2). These results indicate that the N-terminal half of LDPS interacts with OLEO1. Similarly, we tested whether the N- and C-terminal cytoplasmic-facing regions of OLEO1, i.e. OLEO1^1-43^ and OLEO1^116-173^, which flank the central membrane-embedded region of the protein (Tzen et al. 1992; Abell et al. 1997, 2002; refer to illustration in Supplementary Fig. S19C), would relocalize to LDs when coexpressed with LDPS. As shown in Fig. 7D, OLEO1^1-43^-GFP and GFP-OLEO1^116-173^ did not localize to LDs when expressed on their own, as expected (Abell et al. 2004). By contrast, OLEO1^1-43^-GFP, but not GFP-OLEO1^116-173^, colocalized with coexpressed LDPS-mCherry at LDs (Fig. 7D), indicating that the N-terminal region of OLEO1 interacts with LDPS. OLEO1^1-43^ was not relocalized to LDs, however, when coexpressed with either LDAP3 or LDPS^170-307^ (Supplementary Fig. S19D). Collectively, these results indicate that the interaction between the N terminus of OLEO1 with LDPS is specific and not shared with the other LD coat proteins tested here and that it also relies on the N-terminal region of LDPS (i.e. residues 1 to 216), which is distinct from the protein's minimally sufficient LD targeting signal within residues 170 to 307 (Fig. 2).

### Overexpression of *LDPS* in Arabidopsis results in the formation of larger LDs in seeds and young seedlings

Given that both LDPS and OLEO1 are exclusively expressed in seeds and young seedlings (Kretzschmar et al. 2020; Supplementary Fig. S3) and interact at the LD surface (Fig. 7), we explored the functional significance of this interaction by constitutively overexpressing *LDPS* and examining LDs in seeds and young seedlings, where OLEOs are present, or in leaves of older seedlings, where OLEOs are absent. Two independent, homozygous, and single-copy Arabidopsis lines constitutively overexpressing *LDPS* in the WT (Col-0) background were generated (*LDPS OE1* and *LDPS OE2*), and overexpression of *LDPS* was confirmed by RT-PCR (Supplementary Fig. S20A).

**Figure 8.**
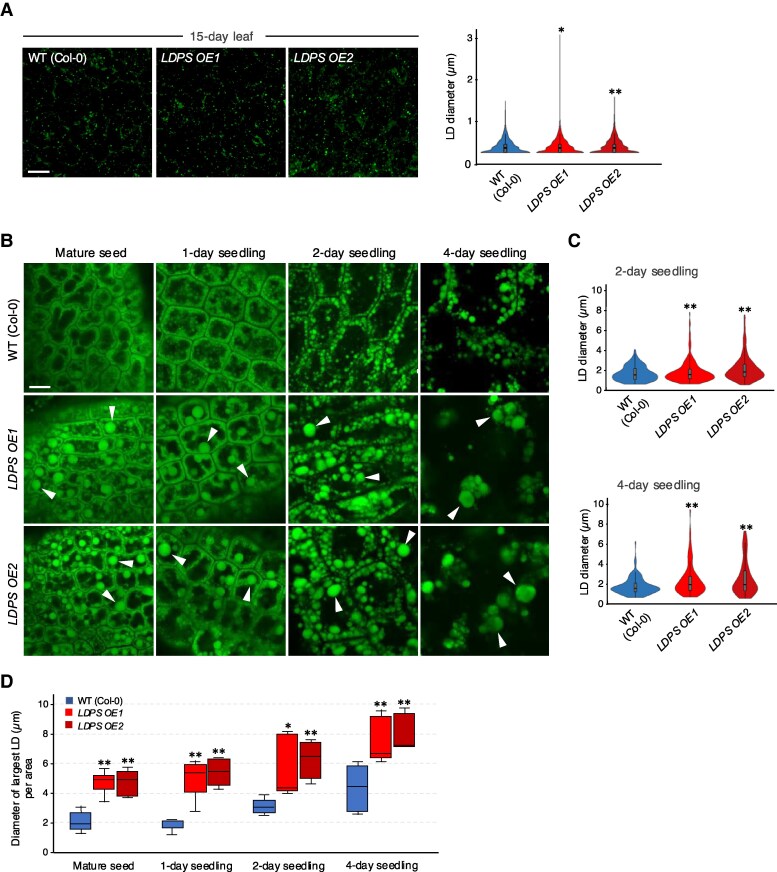
Constitutive overexpression of *LDPS* influences LD size in seeds and seedlings, in Arabidopsis. **A)** LD sizes in leaves of Arabidopsis WT and *LDPS OE* plant lines. Shown on the left are representative CLSM images of BODIPY-stained LDs in leaf epidermal cells of 15-day-old WT (Col-0) and *LDPS OE1* and *LDPS OE2* plants, as indicated with labels. Bar = 10 *µ*m and applies to all the images in the panel. Quantifications of LD diameters are shown in the violin plots on the right. LD diameters were measured using ImageJ, and values shown represent those obtained from 3 biological replicates, with each replicate consisting of 6 to 8 leaf samples per plant line and 2 micrographs per leaf sample, including those shown in the panel. Single and double asterisks represent statistically significant differences at *P* ≤ 0.05 and *P* ≤ 0.01 related to the corresponding WT and *LDPS OE* plant lines, respectively, as determined by a two-tailed Student's *t* test. **B)** Representative CLSM images of BODIPY-stained LDs in hypocotyl or cotyledon cells from Arabidopsis WT (Col-0) and *LDPS OE1* and *OE2* embryos at various developmental stages, including (as indicated with labels) mature seeds, and seedlings 1, 2, and 4 d after the initiation of germination. Arrowheads indicate examples of “supersized” LDs in *LDPS OE* seeds and seedlings that are absent in WT. Bar = 10 *μ*m and applies to all images in the panel. **C)** Quantification of LD sizes in WT and *LDPS OE* embryos in seedlings 2 and 4 d after the initiation of germination. LD diameters were measured using ImageJ, and values shown in violin plots represent those obtained from 3 biological replicates, with each replicate consisting of 6 to 8 leaf samples per plant line and 2 micrographs per leaf sample, including those shown in **(B)**. Double asterisks represent statistically significant differences at *P* ≤ 0.01 related to the corresponding WT and *LDPS OE* plant lines, respectively, as determined by a two-tailed Student's *t* test. **D)** Quantification of the largest LDs in cells of WT and *LDPS OE* seeds and seedlings. Values shown represent the diameters (in μm) of the largest LDs per area for each line, based on the same data set (i.e. micrographs) in **(B)** and **(C)**. Data are summarized in a boxplot with the following details: center line, median; box limits, upper and lower quartiles; whiskers, 1.5 × interquartile range. Single and double asterisks represent statistically significant differences at *P* ≤ 0.05 and *P* ≤ 0.01 related to the corresponding WT and *LDPS OE* plant lines, respectively, as determined by a two-tailed Student's *t* test. Refer to key for the corresponding color and plant line. A summary of the statistical analysis for **(A)**, **(C)**, and **(D)** is given in **Supplementary Table S4**.

As shown in Fig. 8A, the overall profile of LDs in WT and *LDPS OE* leaves looked similar; however, the average size of LDs in the *LDPS OE* lines was, statistically, just slightly larger than that of WT. This suggests that in leaves, which lack *OLEO* expression, the ectopic overexpression of *LDPS* does not have a major influence on LD size and/or abundance. In contrast, in mature seeds and young seedlings (i.e. 1, 2, and 4 d after the initiation of germination), overexpression of *LDPS* led to the appearance of numerous “supersized” LDs that were significantly larger than LDs in WT (Fig. 8, B and C) and similar to the larger-sized LDs observed in *LDPS OE ldps-2* seeds and seedlings (Supplementary Fig. S10). Despite the differences in size in WT and *LDPS* overexpressing mature seeds, LDs in both WT and *LDPS OE* lines continued to grow in size at 1, 2, and 4 d after germination (Fig. 8D), presumably due to LD–LD fusion during postgerminative growth (Miquel et al. 2014).

**Figure 9.**
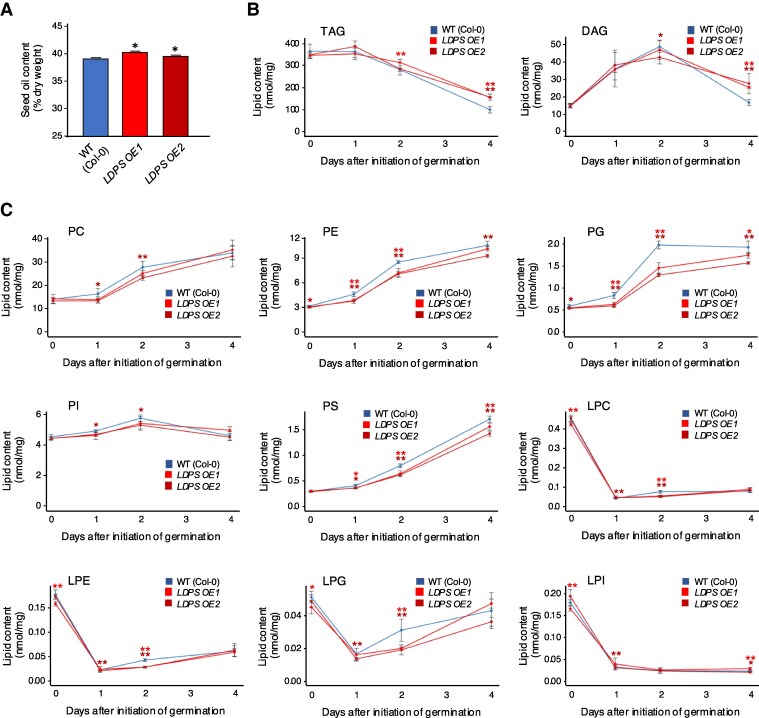
Overexpression of *LDPS* influences Arabidopsis seed oil content and the content and composition of TAG, DAG, and phospholipids during postgerminative growth. **A)** Comparison of Arabidopsis WT (Col-0) and *LDPS OE* seed oil content. Oil content as a percentage of dry weight in mature seeds from each plant line (as indicated with labels) was determined using NMR. Values represent the means ± SD of 3 replicates of 50 mg of seeds for each plant line. Asterisks indicate statistically significant differences (*P* ≤ 0.05) between *LDPS OE* plant lines and WT, as determined by a two-tailed Student's *t* test. **B)** and **C)** Content of TAG, DAG, and polar lipids in Arabidopsis WT (Col-0) and *LDPS OE* seeds and seedlings. Total lipids were extracted from, as indicated by the keys, WT and *LDPS OE1* and *OE2* mature seeds (indicated as 0 d) and seedlings harvested at 1, 2, and 4 d after the initiation of germination and analyzed by LC-MS/MS. Values represent the mean ± SD of the sum of individual lipid molecular species (in nmol/mg dry weight) in **B)** TAGs and DAGs and **C)** various phospholipids, identified from analysis of 5 biological replicates. The individual molecular species of TAG and DAG (Mol percentage) are shown in Supplementary Figs. S21 and S22. Single and double asterisks represent statistically significant differences at *P* ≤ 0.05 and *P* ≤ 0.01 related to WT and *LDPS OE* plant lines, as determined by a two-tailed Student's *t* test. A summary of the statistical analysis for **(A)** to **(C)** is given in Supplementary Table S4. LPC, lysophosphatidylcholine; LPE, lysophosphatidylethanolamine: LPG, lysophosphatidylglycerol; LPI, lysophosphatidylinositol; PC, phosphatidylcholine; PE, phosphatidylethanolamine PG, phosphatidylglycerol, PI, phosphatidylinositol; PS, phosphatidylserine.

In addition to having larger LDs, mature seeds of both *LDPS* overexpression lines contained a higher percentage of storage oil in comparison with WT (Fig. 9A). A more detailed lipidomics analysis further showed that total TAG and DAG content was higher in *LDPS OE* seedlings at 2 and 4 d after germination in comparison with WT (Fig. 9B). By contrast, the abundance of polar lipids in *LDPS OE* seeds and seedlings was generally lower relative to WT (Fig. 9C). These observations are consistent with the presence of the significantly larger LDs in *LDPS OE* seeds and seedlings (Fig. 8), since larger LDs have a lower surface-to-volume ratio and would result in expectedly higher amounts of neutral lipid and lower amounts of phospholipid relative to WT. Moreover, the increased TAG and DAG in *LDPS OE* seedlings (Fig. 9B) might result from decreased accessibility of the larger LDs to the TAG degradation machinery. There were also numerous differences in the composition of various lipid classes in TAG and DAG in the *LDPS OE* seeds and seedlings compared with WT, but their overall lipid profiles were generally similar (Supplementary Figs. S21 and S22). There were no obvious effects, however, of *LDPS* overexpression on postgerminative seedling growth rates based on hypocotyl elongation assays (Supplementary Fig. S20B). Overall, these results support the premise that LDPS is important in Arabidopsis for determining LD size, primarily in seeds and young seedlings, and that larger LDs might diminish the rates of oil breakdown.

### The ectopic coexpression of *LDPS* and *OLEO1* in leaves does not result in the formation of larger LDs

As mentioned, Miquel et al. (2014) previously reported that the transient enlargement of LDs during postgerminative seedling growth in Arabidopsis was due to LD–LD fusion. Given that *ldps* mutants have smaller LDs in mature seeds that do not increase in size during postgerminative growth (Fig. 5 and Supplementary Fig. S9), while *LDPS* overexpressing lines have larger LDs in seeds that continue to increase in size during postgerminative growth (Fig. 8), we asked whether LDPS activity might directly promote LD–LD fusion in plant cells. To test this, we employed a leaf-based LD fusion assay that previously was used to examine and confirm FAT-SPECIFIC PROTEIN OF 27 kDa (FSP27)-dependent LD–LD fusion in plant cells (Price et al. 2020). FSP27 is a mammalian-specific protein that serves as a key regulator of LD–LD fusion in fat-storing white adipocytes (reviewed in Li et al. 2024), and the protein retained this activity when heterologously expressed (either stably or transiently) in plant cells (Price et al. 2020).

**Figure 10.**
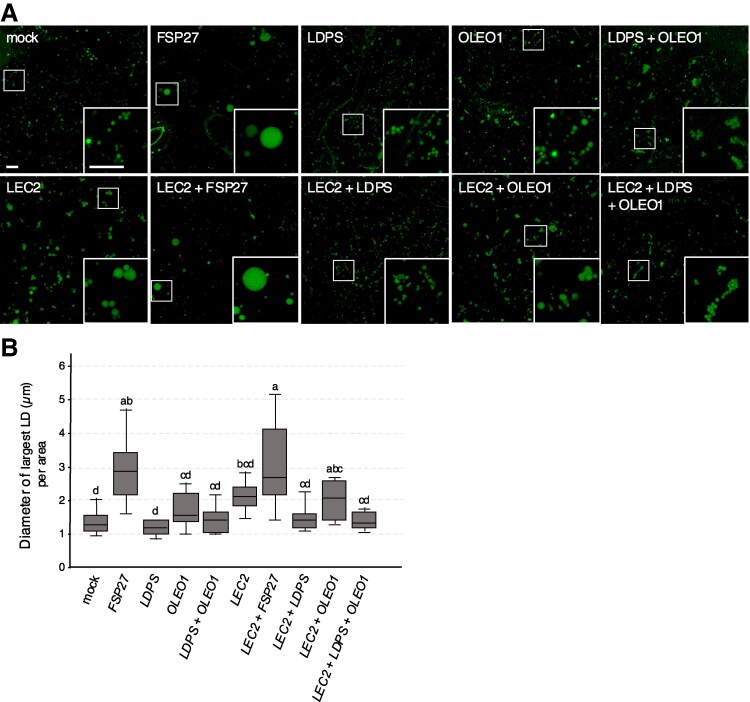
Influence of ectopic expression of *FSP27*, *LDPS*, and/or *OLEO1* in *N. benthamiana* leaves on LD size, with or without coexpressed *LEC2*. **A)** Representative CLSM images of *N. benthamiana* leaf epidermal cells transiently transformed (as indicated with labels) with P19 alone (i.e. “mock” transformation) and mCherry, serving as a cell transformation marker, and also nontagged FSP27, LDPS, and/or OLEO1, including in the absence of LEC2 (top row of images) or in the presence of LEC2 (bottom row of images). LDs were stained with BODIPY. The boxes represent the portion of the cells shown at higher magnification in the insets. Bar = 5 *μ*m and applies to all images and insets in the panel. **B)** Quantification of the largest LDs in *N. benthamiana* leaf cells transformed, as in **(A)**, with P19 and mCherry, nontagged FSP27, LDPS, and/or OLEO1, and with or without LEC2. Values shown represent the maximum diameters (in μm) of the largest LDs per area from 3 biological replicates (i.e. transformations) of 5 images per construct(s), based on measurements of the dataset, including those in **(A)**, using ImageJ. Data are summarized in a boxplot with the following details: center line, median; box limits, upper and lower quartiles; whiskers, 1.5 × interquartile range. Significant differences are indicated at least at *P* ≤ 0.05, as determined by a one-way ANOVA followed by Tukey's post hoc multiple comparison test, and letters above the bars indicate the results of those tests. A summary of the statistical analysis is given in Supplementary Table S4.

As shown in Fig. 10A (top row), expression of mouse (*Mus musculus*) *FSP27* in *N. benthamiana* leaf cells led to an overall increase in the proportion of larger-sized LDs, including several “supersized” LDs that had significantly larger diameters than the LDs in mock-infiltrated leaves (Fig. 10B), which, based on Price et al. (2020), were formed by FSP27-dependent LD–LD fusion. In contrast, the diameters of the largest LDs in cells remained the same when *LDPS* and *OLEO1* were expressed individually or in combination (Fig. 10, A and B and as confirmed by RT-PCR analysis; Supplementary Fig. S23). However, many of the LDs in *LDPS* and *OLEO1* coexpressing cells often were clustered together compared to those in cells expressing either protein alone or in mock-infiltrated leaves (Fig. 10A). Since LD clustering is considered a prerequisite for LD–LD fusion (Yang et al. 2012a; Gao et al. 2017), the data suggested that, in addition to LDPS and OLEO1, there are other seed-specific factor(s) that might be required to facilitate LD–LD fusion in plant cells.

To explore this possibility, we repeated these experiments in cells that also coexpressed Arabidopsis *LEC2*, which is a seed-specific transcription factor that induces multiple genes associated with seed oil accumulation, including those associated with storage lipid biosynthesis and LD packaging (reviewed in Liu et al. 2021). When ectopically expressed in plant leaves, LEC2 upregulates genes for oil production, but induction of *OLEO* genes is not as high as in developing seeds (Santos Mendoza et al. 2005; Baud et al. 2007; Feeney et al. 2013; Kim et al. 2013), resulting in the formation of aberrantly large LDs, presumably due to a lack of sufficient LD coat proteins (Gidda et al. 2016; Pyc et al. 2021). The large LD phenotype, however, can be suppressed by coexpression of additional LD coat proteins, such as LDAPs or OLEOs (Gidda et al. 2016; Pyc et al. 2021). As shown in Fig. 10A (bottom row), expression of *LEC2* in leaf cells resulted in appearance of several enlarged LDs, as expected. Coexpression of *LEC2* and *FSP27*, however, led to the formation of even larger LDs, presumably due to FSP27-mediated LD–LD fusion (Fig. 10, A and B). In contrast, coexpression of *LEC2* with *LDPS* did not result in significantly larger LDs compared to those observed in cells expressing *LEC2* alone (Fig. 10, A and B). Rather, LDs in *LEC2* and *LDPS* coexpressing cells were generally smaller, as were LDs in *LEC2* and *OLEO1* coexpressing cells (Fig. 10B), consistent with results from previous studies showing that expression of additional LD coat proteins can suppress the enlarged LD phenotype observed in cells expressing *LEC2* alone (Gidda et al. 2016; Pyc et al. 2021; Guzha et al. 2024). Coexpression of *LEC2*, *LDPS* and *OLEO1* together also did not yield any supersized LDs akin to those in FSP27-expressing cells (Fig. 10, A and B). However, similar to the results for when *LDPS* and *OLEO1* were coexpressed in the absence of LEC2, LDs often were more clustered in cells coexpressing *LEC2* with both proteins in comparison with the expression of *LEC2* with either protein alone (Fig. 10A). Together, these results indicate that coexpression of *LDPS* and *OLEO1* is sufficient for stimulating LD clustering in leaves, but LDPS either does not function together with OLEO1 to promote LD–LD fusion directly, or there are other seed-specific factors not induced (or not sufficiently induced) by LEC2 in leaves that are required for this activity.

### Freezing treatment prior to seed stratification reveals that the increase in LD size in Arabidopsis seeds and seedlings is an actively mediated process that relies on both LDPS and OLEO1

To elucidate the functional relationships of LDPS and OLEO1 in determining LD size in seeds and seedlings, we generated an Arabidopsis *oleo1 ldps-2* double-mutant line by crossing an *OLEO1* knock-out mutant (Shimada et al. 2008; Miquel et al. 2014) with the *ldps-2* mutant described above (Fig. 5 and Supplementary Fig. S9). We also generated 2 Arabidopsis lines constitutively overexpressing *LDPS* in the *oleo1* mutant background (*oleo1 LDPS OE1* and *oleo1 LDPS OE2*). All 3 plant lines were confirmed by genotyping and progeny analysis (Supplementary Fig. S24). These new lines, along with the corresponding single mutants, WT, and *LDPS OE1* line, provided a comprehensive set of plant lines that altered the ratios of LDPS and OLEO1 in seeds and seedlings and, as such, enabled a genetic analysis of their roles in modulating LD size. In addition, we took advantage of prior studies that showed that disruption of *OLEO1* in Arabidopsis not only increased LD size in seeds (Siloto et al. 2006; Shimada et al. 2008; Miquel et al. 2014), but also increased their susceptibility to freezing-induced LD–LD fusion (Shimada et al. 2008).

**Figure 11.**
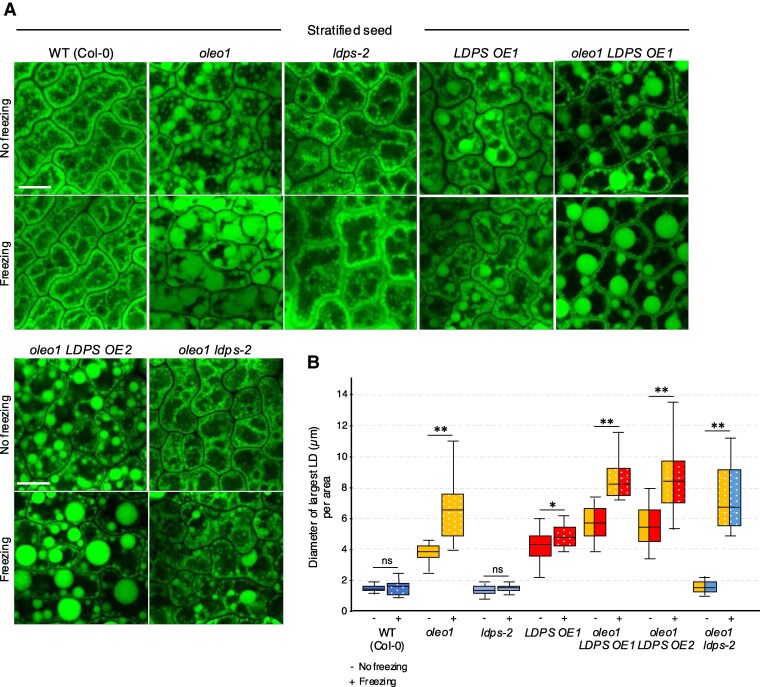
Freezing treatment prior to seed stratification reveals that LDPS and OLEO1 function together to influence LD size in Arabidopsis seeds. **A)** Representative CLSM images of BODIPY-stained LDs in hypocotyl cells in stratified seeds from (as indicated with labels) WT (Col-0), *oleo1*, *ldps-2*, *LDPS OE1*, *oleo1 LDPS OE1* and *OE2,* and *oleo1 ldps-2* plant lines that were either exposed or not exposed to a freezing treatment (i.e. −25 °C for 24 h) prior to seed stratification, according to Shimada et al. (2008). Refer also to Supplementary Fig. S25 for corresponding results for 3-day-old seedlings from the same plant lines exposed or not exposed to freezing treatment prior to seed stratification. Bars = 10 *μ*m and applies to all images in the panel. **B)** Quantification of the largest LDs in WT*, oleo1, ldps-2, LDPS OE1*, *oleo1 LDPS OE1* and *OE2,* and *oleo1 ldps-2-*stratified seeds with (+) and without (−) freezing treatment prior to seed stratification. Values shown represent the maximum diameters (in μm) of the largest LDs per area from 3 biological replicates (i.e. transformations) of 8 images per plant line, based on measurements of the dataset (i.e. micrographs), including those shown in **(A)**, using ImageJ. Data are summarized in a boxplot with the following details: center line, median; box limits, upper and lower quartiles; whiskers, 1.5× interquartile range. Single and double asterisks represent statistically significant differences at *P* ≤ 0.05 and *P* ≤ 0.01; ns, not significant (i.e. *P ≥* 0.05), corresponding to each plant line with or without freezing treatment, as determined by a two-tailed Student's *t* test. A summary of the statistical analysis is given in Supplementary Table S4.

As shown in Fig. 11A, there was a similar distribution of LDs in terms of their size and general morphology in WT seeds that were either exposed or not exposed to a freezing treatment (i.e. −25 °C for 24 h) prior to stratification. By contrast, there was a notable increase in LD size in *oleo1* seeds without freezing treatment, and LD size was significantly enhanced after freezing treatment (Fig. 11A; refer also to Fig. 11B for quantification of the largest LD diameters in seeds of the *oleo1* mutant and all the other plant lines examined). These observations were consistent with prior studies and in agreement with the premise that OLEOs protect LDs against biophysically induced (i.e. freeze–thaw) membrane fusion (Shimada et al. 2008). This premise was further supported by results with *ldps-2* mutant seeds, where the smaller-sized LDs in seeds were unaffected by the freeze–thaw treatment, presumably due to protection by the OLEO protein coat (Shimada et al. 2008). Overexpression of *LDPS* in the WT background (*LDPS OE1*) resulted in larger-sized LDs in seeds, and freezing treatment increased LD size by a small, but statistically significant amount, although well below the increase in size observed in *oleo1* mutant seeds (Fig. 11, A and B). Again, this smaller increase in LD size was likely due to the presence of protective OLEO proteins in the *LDPS OE1* seeds. In contrast, overexpression of *LDPS* in the *oleo1* mutant background (*oleo1 LDPS OE1* or *OE2*) resulted in an enlargement of LDs in seeds, but the increase in LD size after freeze–thaw treatment was substantially higher than when *LDPS* was overexpressed in the WT background (Fig. 11, A and B). Surprisingly, disruption of both *OLEO1* and *LDPS* (*oleo1 ldps-2*) resulted in seeds containing LDs that were more similar in size to WT, and not to the larger LDs observed in *oleo1* single-mutant seeds (Fig. 11, A and B), indicating that *LDPS* and *OLEO1* have an epistatic relationship where OLEO1 suppresses the ability of LDPS to increase LD size. Freeze–thaw treatment of *oleo1 ldps-2* double-mutant seeds resulted in enlarged LDs in seeds, some of which were similar in size to the enlarged LDs observed in the *oleo1* single-mutant freeze-thawed seeds. However, there was a more heterogeneous distribution of LD sizes in the *oleo1 ldps-2* double-mutant seeds (Fig. 11, A and B). Notably, the LD phenotypes in seeds for each plant line persisted during postgerminative seedling growth (i.e. 3 d after the initiation of germination) (Supplementary Fig. S25, A and B), with differences in the largest LDs in seedlings exposed or not exposed to the freezing treatment prior to seed stratification often being even more apparent than those in seeds. Moreover, violin plots of the diameters of all the LDs in WT, *oleo1*, *ldps-2*, and *oleo1 ldps-2* seedlings demonstrated that the vast majority of LDs in the *oleo1 ldps-2* double mutant were more similar in size to those in *ldps-2* and WT than *oleo1* in both freezing-treated or untreated conditions (Supplementary Fig. S25C), confirming a key role for LDPS in modulating LD size in the *oleo1* mutant background.

## Discussion

The storage of neutral lipids in the aqueous interior of the cell requires a wide array of proteins to mediate the highly orchestrated, stepwise packaging of lipids into nascent LDs at the ER. In seeds, the predominant LD coat proteins are OLEOs, which are cotranslationally synthesized on ER membranes and then partition into growing LDs to presumably help drive their formation from the ER surface (reviewed in Huang 2018). In nonseed organs, however, *OLEO* expression is reduced or absent, suggesting that other proteins are involved in LD biogenesis and function in these tissue/cell types. Indeed, in the past ∼10 yr, several other proteins, such as SEIPINs, LDAPs, LDIP, and VAP27-1, have been shown to play important roles in LD production in both seed and nonseed tissues (reviewed in Guzha et al. 2023). In some cases, such as the SEIPINs, these LD biogenetic proteins and their associated activities are well conserved across the eukaryotic lineages (Cai et al. 2015), while other proteins, such as LDAP and LDIP, do not share high sequence similarity to proteins outside of plants (Horn et al. 2013; Pyc et al. 2017). Here, we show that a recently identified LD protein called LDPS (Kretzschmar et al. 2020) plays a prominent role in promoting LD enlargement in seeds and young seedlings through a process that includes LD–LD fusion.

### LDPS is a plant- and seed-specific protein that has multiple types of LD localization signals

LDPS was originally identified in a proteomics analysis of Arabidopsis LDs isolated from seeds and seedlings (Kretzschmar et al. 2020). Microarray and RT-PCR analysis (Supplementary Fig. S3) showed that the gene is specifically expressed at the latter stages of seed development and early stages of postgerminative seedling growth, but not in other plant organs or developmental stages. Cell biology studies confirmed that Arabidopsis LDPS was localized to LDs (Kretzschmar et al. 2020), and phylogenetic analyses revealed it is a plant-specific protein with homologs present in both seed-bearing and seedless plants, but not in earlier plant-related species, such as algae (de Vries and Ischebeck 2020). Here, we extended these studies by showing that LDPS homologs from other seed-bearing and seedless plants also target specifically to LDs (Fig. 1), while 18CS proteins, which show sequence similarities with the LDPS-type proteins, do not (Fig. 1 and Supplementary Fig. S1). Thus, it appears that LDPS-type proteins developed the capacity to localize to LDs during the evolutionary transition of plants from aqueous to terrestrial environments and, in doing so, perhaps acquired a role(s) in LD-related processes associated with desiccation tolerance (de Vries and Ischebeck 2020; Bouchnak et al. 2023).

To better understand how LDPS localizes to LDs, we performed a series of cell biological experiments to define the protein's LD targeting information. A mutational analysis of Arabidopsis LDPS, including truncated or internal regions of LDPS fused to GFP, identified a region between amino acids 170 and 370 as being minimally sufficient for LD targeting (Fig. 2). Subsequent comparison of predicted 3D protein structures of Arabidopsis LDPS and 18CS revealed a polypeptide sequence (i.e. residues 191 to 242) within this sufficiency region that had distinct sequence and structural variability between the 2 proteins (Fig. 3). Swapping this “variable” region in LDPS into the corresponding region of 18CS conferred the ability of the resulting 18CS hybrid protein to target to LDs (Fig. 3), confirming that this sequence in LDPS contains LD targeting information. A closer inspection of this region in LDPS further revealed that it contains a predicted amphipathic α-helix that is more hydrophobic overall and enriched in large, hydrophobic amino acids than the corresponding region in 18CS, as well as a conserved proline residue that is absent in 18CS (Fig. 4). Amphipathic α-helices are known to be important for targeting of proteins from the cytoplasm to the surface of LDs, where protein association is mediated by large hydrophobic side chains that bind to “packing defects” in the LD monolayer surface that transiently expose the hydrophobic core of the LD lipid interior (reviewed in Dhiman et al. 2020; Olarte et al. 2022). Replacing the large hydrophobic residues with smaller hydrophobic residues has been shown to abolish the targeting of several proteins to LDs (Prévost et al. 2018; Olarte et al. 2020). In our studies, replacement of the large hydrophobic residues in LDPS with smaller hydrophobic valines did not abolish LD targeting altogether, but did reduce the fidelity of targeting (Fig. 4). However, we also showed that the conserved proline residue in LDPS, which is not part of the predicted amphipathic α-helix, but rather within a putative hairpin/turn region adjacent to the helix (Fig. 4), is critically important for LD association; replacing this proline with alanine, while the amphipathic α-helix sequence was fully intact, completely abolished LD targeting (Fig. 4). These data indicate that both the amphipathic α-helix and proline turn region in LDPS contribute to its LD association. Interestingly, several other proteins known to rely on proline hairpin motifs for their targeting to LDs do so by sorting via the ER (Dhiman et al. 2020; Olarte et al. 2022). Whether the amphipathic α-helix and proline turn region in LDPS both physically interact with the LD membrane surface and whether they mediate the targeting of LDPS to LDs directly from the cytoplasm and/or indirectly via the ER remain to be investigated.

In addition to the unique amphipathic α-helix and proline turn region, the localization of LDPS to LDs possibly includes an interaction with OLEO1 on the LD surface. Protein relocalization studies, for instance, revealed that the N-terminal half of LDPS interacts with the N-terminal region of OLEO1 (Fig. 7 and Supplementary Fig. S19), which was consistent with yeast mbSUS assays showing an interaction of full-length LDPS and OLEO1 (and OLEO2) (Fig. 7). Since OLEOs are synthesized on the ER and traffic to LDs during the early stages of LD biogenesis (Huang and Huang 2017), it is possible that they sterically inhibit the association of LDPS to LDs via protein “crowding effects,” which are known to play a major role in determining overall LD protein composition (Kory et al. 2016). Thus, the binding of LDPS directly to OLEOs would provide an alternative mechanism to ensure its localization with LDs in mature seeds. Thereafter, when OLEOs are degraded during postgerminative seedling growth (Deruyffelaere et al. 2015, 2018; Kretzschmar et al. 2018), LDPS might subsequently bind to LDs via its amphipathic α-helix/proline turn region. Whether there are functional differences in LDPS activity when associated with LDs via its interaction with OLEO1 or the amphipathic α-helix/proline turn region is presently unknown. Regardless, these findings provide a possible mechanism for understanding how LDPS activity might be modulated in seeds and young seedlings through differential types of interactions with LDs. Notably, several other LD proteins in eukaryotes, such as the perilipins (PLINs) and FSP27 in mammals, are also known to interact with LDs in multiple ways, including protein–protein- and protein–lipid-based mechanisms that modulate protein functionality on the LD surface (reviewed in Griseti et al. 2024).

### LDPS modulates LD size in seeds and seedlings in a process that involves OLEO1 and LD–LD fusion

The expression of *LDPS* during the latter stages of seed development and early stages of postgerminative growth in Arabidopsis suggests that it participates in a seed-specific process(es) related to storage oil synthesis, accumulation, and/or degradation. Indeed, disruption of *LDPS* in 2 different plant lines results in seeds that contain less oil in comparison with WT, as well as smaller LDs that do not increase in size during postgerminative seedling growth (Fig. 5 and Supplementary Fig. S9). This LD phenotype is similar to that seen in *pux10* mutant seeds, which lack the ability to properly degrade LD proteins, including OLEOs, during postgerminative seedling growth. As a result, OLEO protein abundance remains high, and LDs have reduced capacity to undergo fusion (Deruyffelaere et al. 2015, 2018; Kretzschmar et al. 2018). LDPS does not appear to function in the PUX10 pathway, however, since OLEO protein amount is generally similar between WT and *ldps* mutant seeds and seedlings (Fig. 6 and Supplementary Fig. S16). These observations led us to hypothesize that LDPS is instead involved, either directly or indirectly, in the process of LD–LD fusion. A loss of LDPS-promoting fusogenic activity would explain the smaller LD phenotype observed in *ldps* mutant seeds, as well as the inability of LDs to undergo fusion during postgerminative seedling growth (Fig. 5 and Supplementary Fig. S9). Conversely, an increase in LDPS-promoting fusogenic activity could account for the larger LDs observed in seeds and during postgerminative seedling growth in LDPS overexpressing plant lines (Fig. 8).

To test directly for LDPS fusogenic activity, we employed a leaf-based assay that we used previously to show that mammalian FSP27 mediates the formation of enlarged LDs in plant cells via LD–LD fusion (Price et al. 2020). FSP27 is a fat-specific protein in mammals that promotes LD–LD fusion and formation of the very large, unilocular LDs observed in cells of white adipose tissue. Here, we showed that heterologous expression of mouse *FSP27* in leaves results in formation of larger, supersized LDs, as expected (Price et al. 2020), but expression of *LDPS* did not (Fig. 10). Coexpression of *LDPS* with *OLEO1* and/or *LEC2* in leaves also showed no evidence of LD fusion, although LD clustering, which is known to be a prerequisite to LD–LD fusion in mammals (Jambunathan et al. 2011; Lohmann et al. 2013), was readily observed (Fig. 10). These observations suggest that there might be other seed-specific factors and/or physiological conditions that are not fully recapitulated in the leaf-based system to support LDPS-dependent LD–LD fusion. Alternatively, or in addition to, there may be factors specific to leaves that inhibit LDPS activity. The LD proteome is known to be quite different between seeds and leaves (Brocard et al. 2017; Kretzschmar et al. 2020; Omata et al. 2024; Scholz et al. 2025), and perhaps, these differences contribute to the stimulatory effects of LDPS in seeds and/or the inhibitory activities in leaves. These possibilities are supported by the observation that larger-sized LDs were observed in seeds and young seedlings in plant lines overexpressing *LDPS* (Fig. 8), while only subtle changes in LD size were observed in leaves (Fig. 8), despite confirmation of *LDPS* overexpression in leaf tissues (Supplementary Fig. S20).

**Figure 12.**
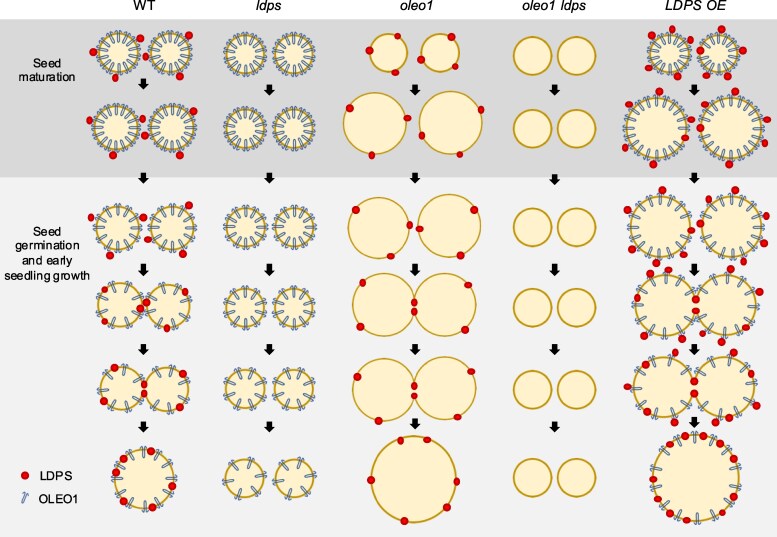
Model for the roles of LDPS and OLEO1 in modulating LD size in seeds and young seedlings in Arabidopsis. In WT seeds, LDs are coated primarily with OLEOs, including, as shown, OLEO1, as well as LDPS, which is a low-abundance protein that localizes to LDs using 2 potential mechanisms: an amphipathic α-helix and proline hairpin motif that binds directly to the LD surface or its N-terminal region that associates with LD-associated OLEO1. When OLEO1 concentration is high, LDPS associates with LDs primarily through interaction with OLEO1. When OLEO1 concentration is low, LDPS binds directly to the LD surface. In doing so, LDPS promotes the fusion of LDs in a process that likely requires another hitherto unknown seed-specific protein(s) or molecular component(s), since the expression of LDPS on its own in leaves is not sufficient for inducing robust LD–LD fusion. During seed maturation in WT (left side of top panel), OLEO1 concentration is high, and although LDPS accessibility to the LD surface is limited, it still contributes to a small amount of LD–LD fusion. This activity is supported by observations of LDs in *ldps*-maturing seeds, which lack LDPS resulting in slightly smaller, but statistically significant differences in comparison with WT. In *oleo1* maturing seeds, OLEO1 is absent and LDPS has increased access to the LD surface, resulting in enhanced LD–LD fusion and increased LD size relative to WT. By contrast, LDs do not increase in size in *oleo1 ldps* double-mutant maturing seeds, since LDPS (and OLEO1) is absent, and no LD–LD fusion takes place. In maturing seeds of *LDPS OE* lines, OLEO1 concentration is similar to WT, but the higher relative concentration of LDPS increases competitive binding for the LD surface, which stimulates LD–LD fusion and growth. During seed germination and early seedling growth (bottom panel of figure), OLEO1 concentration is reduced via the PUX10-mediated pathway, which increases the opportunity for LDPS to bind to the LD surface. This increased binding of LDPS promotes LD–LD fusion and the enlargement of LDs observed at 2 to 4 d postgermination. The relative concentration of LDPS is also likely enhanced by gene expression, which is known to be highest for LDPS at earliest stages of postgerminative seedling growth. In *ldps* mutant seedlings, LDs remain small during postgerminative growth, despite a reduction in OLEO1 concentration, because LDPS is absent and unable to promote LD–LD fusion. In *oleo1* mutant plants, on the contrary, the larger LDs observed in mature seeds continue to grow in size, because LDPS is present and promotes further LD–LD fusion. In *oleo1 ldps* double-mutant seedlings, LDs remain small, since LDPS is absent and unable to promote LD–LD fusion. Lastly, in the *LDPS OE* seedlings, the large LDs present in mature seeds continue to grow in size, since LDPS concentration is high, resulting in continued LD–LD fusion and growth. Additional support for an unknown, seed-specific factor/component(s) in LDPS-promoted LD–LD fusion is the lack of any obvious changes in LD size in *LDPS OE* leaves. See the main text for additional details on potential LDPS activity.

Since we were unable to utilize the leaf-based system to characterize functional relationships of OLEO1 and LDPS in modulating LD–LD fusion, we performed a comprehensive analysis of LD size in seeds and seedlings from several different plant lines with altered ratios of *OLEO1* and *LDPS* expression, including WT, *oleo1* and *ldps-2* single mutants, *LDPS* overexpressed in either the WT or *oleo1* background, and an *oleo1 ldps-2* double mutant (Fig. 11). Given that the primary readout for these experiments was changes in LD size, we considered the possibility that LD–LD fusion might be induced by at least 2 mechanisms, including an active, protein-mediated process and a more passive, biophysically induced process due to LD membrane instability. To help distinguish between these possibilities, we included a freeze–thaw treatment of seeds prior to visualization of LDs. Prior studies have shown that disruption of *OLEO* gene expression in Arabidopsis not only results in larger LDs in mature seeds (Siloto et al. 2006; Shimada et al. 2008; Miquel et al. 2014), but also increases the susceptibility of LDs to freezing-induced LD–LD fusion. These and other observations suggested that at least 1 role for OLEO proteins in LD biology was to stabilize the membrane surface and prevent LD–LD fusion, particularly during stresses such as seed desiccation or freezing and thawing (Shimada et al. 2008). Consistent with this, we found that LDs in *oleo1* seeds are larger than in WT, and freeze–thaw treatment results in a further, dramatic increase in LD size in *oleo1* seeds, but not in WT (Fig. 11; Shimada et al. 2008). Surprisingly, however, a comparison of LDs in seeds of *oleo1* and *oleo1 ldps-2* revealed that LDs in the double mutant are more similar in size to WT than *oleo1* (Fig. 11). These results and those based on a similar assessment of LDs sizes in germinated seedlings in the same plant lines (Supplementary Fig. S25) indicate that OLEO1 is epistatic to LDPS, masking the ability of LDPS to promote an increase in LD size. Also, somewhat surprisingly, freeze–thaw treatment of *oleo1 ldps-2* seeds results in appearance of much larger LDs, but the distribution of LD sizes in the double-mutant seedlings was again more similar to WT than *oleo1* (Supplementary Fig. S25), suggesting that LDPS also contributes to the enlargement of LD size after freeze–thaw treatment. Freeze–thaw treatment of all plant lines also consistently showed a positive association between the presence of OLEO1 and protection against LD–LD fusion. For instance, WT, *LDPS OE1*, and *ldps-2*, each of which contain presumably native levels of *OLEO1* expression, are all protected against LD–LD fusion after freeze–thaw treatment, despite having differences in LD sizes in nontreated seeds (Fig. 11). There was also a slight increase in LD size in seeds after freeze–thaw treatment of the *LDPS OE1* line, but it was not as large as the change observed when *LDPS* was overexpressed in the *oleo1* mutant background (Fig. 11). Taken together, these and the other results presented in this study support the working model shown in Fig. 12, whereby LDPS and OLEO1 function together to influence LD size in a manner that includes OLEO1-mediated suppression of LDPS activity followed by LDPS-dependent LD–LD fusion in both seeds and seedlings of Arabidopsis (see figure legend for details).

### Comparison of LDPS and FSP27 provides insights to the potential fusogenic activity of LDPS

Although expression of mammalian FSP27 induces a significant enlargement of LDs in plant leaves, while LDPS does not (Fig. 10), it is still worthwhile to consider FSP27 as a model for the potential LD fusogenic activity of LDPS. FSP27 is a member of the cell-death-inducing DNA fragmentation factor-α-like effector (CIDE) protein family, whose primary role is to bind to LDs and facilitate their fusion and growth in different tissues and cell types in mammals (reviewed in Xu et al. 2024). CIDE proteins interact to form dimers and oligomers that accumulate at the fusion plate between 2 LDs. There, the proteins help facilitate the transfer of neutral lipids from 1 LD to the other, which is driven by internal pressure differences between the LDs (Xu et al. 2024).

Despite a lack of primary sequence similarity between plant LDPS and mammalian FSP27 (Supplementary Fig. S26), the 2 proteins conspicuously share several structural and functional characteristics, including: a generally unstructured N-terminal region; an amphipathic α-helix and adjacent hydrophobic region involved in LD targeting/association; and the ability to self-associate and also bind to other proteins, including some that are colocated on the LD surface. CIDE proteins also have the capacity to bind to phosphatidic acid (PA), which is thought to be important for facilitating lipid transfer between LDs (Barneda et al. 2015). Although we did not measure PA content in WT, *ldps*, and *LDPS OE* seeds and seedlings (given the difficulty of PA detection/quantification by MS; Ogiso et al. 2008), our lipidomics analysis did reveal significant changes in DAG content and composition in *ldps* mutant seeds and seedlings (Fig. 6). DAG and PA are interconvertible by a kinase–phosphatase system (Testerink and Munnik 2005), and perhaps, LDPS binding of PA (or DAG) alters its accessibility to metabolically relevant enzymes, thereby altering the steady-state levels of DAG and PA in seeds and seedlings. Alternatively, or in addition to, the altered DAG content in *ldps* mutant seeds and seedlings (Fig. 6) could be due to increased TAG turnover (Eastmond 2006) and/or alterations in TAG remodeling (Parchuri et al. 2024). Overexpression of *LDPS* in seeds and seedlings also resulted in changes in DAG content and composition relative to WT (Fig. 9B and Supplementary Fig. S22). However, there were no clear trends in these data compared with those for the *ldps* mutants that would suggest a potential, underlying LDPS-based mechanism. Further investigation will be required to determine whether the functionality of LDPS involves the binding or modulation of specific lipid metabolites.

As mentioned, CIDE proteins and LDPS are also similar in that they can form homotypic and heterotypic protein associations. For LDPS, homotypic interactions were observed in the mbSUS assay (Fig. 7) and an AlphaFold simulation supports the potential of the protein to form homodimers (Supplementary Fig. S27; for information on the formation of CIDE/FSP27 homodimers, refer to Xu et al. 2024). Additionally, for CIDE proteins, heterotypic protein associations are known to stimulate or inhibit CIDE protein activity (Xu et al. 2024). One of the proteins that stimulate FSP27's fusogenic activity is the PLIN isoform 1 (PLIN1) (Grahn et al. 2013; Sun et al. 2013). This is particularly notable, since PLIN1 is the predominant LD coat protein in adipocytes (Servetnick et al. 1995). In a similar manner, LDPS interacts with OLEO1 and possibly other OLEOs (e.g. OLEO2) (Fig. 7), which are abundant LD coat proteins in Arabidopsis seeds (Kretzschmar et al. 2020). However, unlike the interaction of FSP27 and PLIN1, which stimulates FSP27 activity, the interaction between LDPS and OLEO1 appears to inhibit LDPS activity (Fig. 11). This suggests that there are other proteins in seeds that contribute to LDPS-dependent LD–LD fusion and/or that there are protein-binding partners expressed in leaves that inhibit LDPS activity; although since *LDPS* is not normally expressed in leaves (Kretzschmar et al. 2020; Supplementary Fig. S3), any suppression in those tissues is probably not as biologically relevant. Nonetheless, we attempted to identify other potential binding partners for LDPS by conducting a yeast two-hybrid (Y2H) screen using LDPS as bait and a cDNA prey expression library derived from a variety of Arabidopsis tissues. Overall, several interesting protein candidates were identified (Supplementary Table S1), including some that, like LDPS, are significantly enriched in LD fractions isolated from Arabidopsis seeds and seedlings (Kretzschmar et al. 2020). These candidate proteins will be useful in future experiments aimed at identifying proteins that stimulate or inhibit LDPS activity in seeds or leaves, respectively.

### Physiological significance of regulating LD size in plant cells

In recent years, numerous LD-related proteins have been identified in plants that, when their expression is altered, modulate LD size in seeds (Pyc et al. 2017; Chapman et al. 2019; Ischebeck et al. 2020; Guzha et al. 2023). These observations raise intriguing questions about why LD size is important and suggest that LD size has been fine-tuned throughout plant evolution to meet important physiological needs. LD size is known to influence access of the neutral lipid (i.e. TAG) core to lipases and other enzymes involved in storage lipid turnover, with smaller and larger LDs yielding relatively greater and reduced accessibility, respectively. Seed oil synthesis occurs during a relatively short developmental period of embryogenesis, and toward the end of oil accumulation there is a brief upregulation of the TAG degradation machinery that results in both TAG remodeling and an overall decrease in seed oil content (Eastmond 2006; Parchuri et al. 2024). Regulation of LD size could be 1 mechanism that balances accessibility of TAG to the degradation machinery during seed oil accumulation, and the need to rapidly breakdown TAG *en masse* during postgerminative seedling growth. Notably, disruption of *LDPS* expression results in slightly smaller LDs and lower seed oil content in mature seeds (Figs. 5 and 6), while overexpression of *LDPS* results in larger LDs and elevated seed oil content in mature seeds (Fig. 8). Modulation of other LD-related genes in plants also results in larger LDs in seeds and a commensurate increase in seed oil content (Guzha et al. 2023). These observations support the hypothesis that LD size contributes to steady-state seed oil accumulation by regulating accessibility of the TAG core to the lipid breakdown machinery. However, there are also examples where modulating LD proteins presence or absence results in an increase in LD size, but no apparent change in seed oil content, such as LDAP1 (Gidda et al. 2016), PUX10 (Kretzschmar et al. 2018), VAP27-1 (Greer et al. 2020), SEIPIN2/3 (Greer et al. 2020), or type-II metacaspase proteases (Liu et al. 2024). These observations indicate that further experiments are required to better understand the relationship and LD size and oil content in seeds under different cellular and physiological contexts.

In germinated seeds, TAG is broken down to support postgerminative seedling growth prior to photosynthetic establishment. Under this physiological need, it might be beneficial to have smaller LDs with greater access to TAG degradation machinery to facilitate more rapid TAG turnover, as observed in mammalian adipose tissue, where smaller LDs are produced from the large unilocular LD to help facilitate TAG breakdown (Grabner et al. 2021). In germinated Arabidopsis seedlings, however, LD size increases during the first few days of postgerminative seedling growth, prior to a downward adjustment in LD size in older seedlings and leaves (Miquel et al. 2014; Pyc et al. 2017; Krawczyk et al. 2022b). There are at least 2 possible explanations for this increase in LD size in young seedlings. First, TAG breakdown and the eventual conversion of acetyl CoA to sucrose in seedlings are initially facilitated by the β-oxidation of fatty acids within glyoxysomes, which are specialized peroxisomes that contain enzymes involved in the glyoxylate cycle that produces precursors for gluconeogenesis (Graham 2008). Perhaps, the β-oxidation machinery in germinated seedlings is rate limiting compared with TAG lipase activity, and there is a physiological benefit to slowing TAG breakdown in order to more efficiently couple fatty acid availability with β-oxidation rates; hence, the need to increase LD size to decrease availability to the TAG degradation machinery. A second possibility is that the turnover of OLEO proteins during postgerminative seedling growth (Deruyffelaere et al. 2015, 2018; Kretzschmar et al. 2018) results in a rapid decrease in availability of LD surface-binding proteins. This could lead to LDs being less stable in the aqueous environment of the cell due, in part, to increased exposure of the neutral lipid core through “packing defects” in the phospholipid monolayer. This would be thermodynamically and biologically unfavorable, and thus, there would be physiological benefit from the enlargement of LDs, via LD–LD fusion, to match the availability of the remaining LD surface-associated proteins. Exactly how LDPS functions at the molecular level to help facilitate LD enlargement during seed development and seed germination is fertile ground for future investigation.

## Materials and methods

### Plant material, growth conditions, and transformations

Experiments with Arabidopsis (*A. thaliana*) were performed using either the WT, Nos-0, or Col-0 ecotypes or derivatives thereof, including transfer (T)-DNA insertion mutant lines *ldps-1* (psh11205), obtained from RIKEN BRC Experimental Plant Division (https://epd.brc.riken.jp/en/arabidopsis), *18cs* (SALK_123069C), obtained from the Arabidopsis Biological Resource Centre (ABRC, https://abrc.osu.edu), and the previously described *oleo1* (SM_3_29875) and *pux10-2* (SALK_139056) mutants (Shimada et al. 2008; Miquel et al. 2014; Kretzschmar et al. 2018). Genotyping and progeny analysis were used to confirm T-DNA single-insertion homozygous plants, and RT-PCRs confirmed disruption in gene expression (see “PCR and RT-PCR” for additional details).

The Arabidopsis *ldps-2* mutant plant line was generated using CRISPR/Cas9-based genome editing, according to the method described by Wang et al. (2015). Briefly, WT Arabidopsis (Col-0) was transformed via the floral dip method (Clough and Bent 1998 ) with *Agrobacterium tumefaciens* strain GV3101 containing pBEE401E/LDPSsgRNA, a binary plasmid encoding *CAS9* and a pair of single-guide RNAs corresponding to sequences in the *LDPS* open reading frame (ORF); refer to “Plasmid construction” for additional information on the construction of pBEE401E/LDPSsgRNA and all other binary vectors used in this study. Progeny analysis, genotyping, and RT-PCR were used to select mutant plants that were homozygous for the edited *LDPS* gene, deficient of the *CAS9*-containing T-DNA, and disrupted in *LDPS* expression, respectively. The edited *LDPS* gene in the *ldps-2* mutant line was also confirmed by sequencing the PCR product obtained using genomic DNA (gDNA) (isolated from rosette leaves of 4-wk-old *ldps-2* plants) and *LDPS* gene-specific primers. Arabidopsis plant lines stably overexpressing *LDPS* either in the WT (Col-0) or in the *ldps-2* or *oleo1* mutant backgrounds, were generated using the floral dip method (Clough and Bent 1998) with *Agrobacterium* GV3101 containing the binary plasmid pMDC32-CVMV/LDPS (see “Plasmid construction” for details). Progeny analysis and PCR or RT-PCR were then used to select independent, single-copy, homozygous lines overexpressing *LDPS*. The *oleo1 ldps-2* double-mutant line was generated by crossing the *oleo1* and *ldps-2* mutants (see above), and F_1_ heterozygous progeny were advanced to homozygosity and confirmed by progeny analysis and genotyping.

Arabidopsis plants were grown in soil with a 16-h day/8-h night cycle at 22 °C and 50 μE m^−2^ s^−1^ light intensity. Arabidopsis plants were grown also on plates, whereby seeds were surface-sterilized with ethanol and sown on plates containing half-strength Murashige and Skoog (MS; Sigma-Aldrich) (Murashige and Skoog 1962) and then stratified for 3 d in the dark at 4 °C before transferring to a growth chamber for the initiation of germination, with similar growth conditions to those described above. Alternatively, for assessments of seed germination and seedling growth rates or hypocotyl elongation assays and lipidomics, stratified seeds were germinated in constant light or (after 6 h in the light to initiate germination) in the dark, respectively; refer to “Miscellaneous plant-based assays” for additional details, including growth conditions used for seed-freezing experiments.

*N. benthamiana* plants, serving as a model system for assessing intracellular localization of plant proteins (Sparkes et al. 2006), were grown in soil in a growth chamber with a 16-h day/8-h night cycle at 22 °C and 50μE m^−2^ s^−1^ light intensity. Leaves of ∼4-wk-old plants were infiltrated with *Agrobacterium* strain LBA4404 harboring specific expression vectors. All (co)infiltrations were performed also with pORE04/P19 containing the *tomato bushy stunt virus* (TBSV) gene RNA-silencing suppressor *P19* (Petrie et al. 2010). For *N. benthamiana* leaf-based LD fusion assays, all (co)infiltrations were also performed with mCherry (i.e. pMDC32/ChC; see below), serving as cell transformation marker protein. Refer to McCartney et al. (2005) and Cai et al. (2015) for additional details on *Agrobacterium* growth, transformation, and infiltration procedures.

### Plasmid construction

Molecular biology reagents were purchased from New England Biolabs, Thermo Fisher Scientific or Invitrogen, and custom oligonucleotides were synthesized by Sigma-Aldrich. Double-stranded synthetic DNA encoding larger portions of genes (or modified versions thereof) were purchased from Integrated DNA Technologies (IDT). Sequence information for all primers used to construct new plasmids, as described below, as well as primers used in gDNA PCRs and RT-PCRs, is listed in Supplementary Table S2. All plant expression plasmids used in this study were driven by the *35S* cauliflower mosaic virus (CaMV) promoter, with exception of pMDC32-CVMV/LDPS and pBEE401E/LDPSsgRNA (see below), which contain the cassava vein mosaic virus (CVMV) constitutive promoter (Verdaguer et al. 1996) and Arabidopsis *EGG CELL1* egg-cell-specific promoter (Wang et al. 2015), respectively. All newly constructed plasmids were verified using automated DNA sequencing performed at either the University of Guelph Genomics Facility, Retrogen Inc., or Plasmidsurus.

The construction of pMDC32/AtLDPS-mCherry, encoding the full-length ORF of Arabidopsis *LDPS* (AT3G19920.1), but without its stop codon, and a C-terminal-appended monomeric mCherry (mCherry) tag, has been described previously (Kretzschmar et al. 2020). pMDC32/mCherry-LDPS, encoding N-terminal mCherry-tagged Arabidopsis LDPS, was constructed by PCR-amplifying the full-length ORF of *LDPS*, including its stop codon, using Arabidopsis cDNA derived from mRNA isolated from mature seeds as template DNA and gene-specific primers that included flanking 5′ and 3′ attB sites (Supplementary Table S2). Resulting PCR products were subcloned using Gateway technology into the pDONR/Zeo cassette vector (Curtis and Grossniklaus 2003) yielding pDONR/LDPS and then pMDC32/ChN. The latter is a binary vector encoding mCherry and an adjacent 3′ in-frame cloning site, which enables expression of a fusion protein with N-terminal-appended mCherry (Doner et al. 2021). Similarly, pMDC32/LDPS, encoding nontagged Arabidopsis LDPS, was constructed by subcloning the LDPS ORF from pDONR/LDPS into pMDC32 using Gateway technology. pMDC32-CVMV/LDPS, encoding nontagged Arabidopsis LDPS driven by the *CVMV* promoter (i.e. *CVMV Pro::LDPS* transgene), was constructed by first PCR-amplifying the *CVMV* promoter sequence from the plant expression vector pB110 (Shockey et al. 2015) with primers that included flanking 5′ *Hind*III and 3′ *Kpn*I restriction sites (Supplementary Table S2). PCR products were then digested with *Hind*III and *Kpn*I and ligated into similarly digested pMDC32/ChC (Kretzschmar et al. 2020), which removed the *CaMV* promoter and replaced it with the *CVMV* promoter, yielding pMDC32-CVMV/ChC. Thereafter, the *LDPS* ORF, including its stop codon (ensuring the *LDPS* ORF was not translationally fused to the 5′ end of mCherry ORF), was subcloned using Gateway technology from pDONR/LDPS into pMDC32-CVMV/ChC, yielding pMDC32-CVMV/LDPS.

Construction of pMDC32/AT3G19920.2-mCherry, encoding the longer, alternatively spliced variant of Arabidopsis LDPS, i.e. AT3G19920.2 (refer to Supplementary Fig. S3 for an illustration depicting the AT3G19920.1, referred to as LDPS in this study, and *AT3G19920.2* transcripts derived from the Arabidopsis *LDPS* gene locus) with a C-terminal-appended mCherry-tag, was carried out as follows. Overlapping portions of the 5′ and 3′ halves of the AT3G19920.2 ORF were amplified using 2 pairs of gene-specific primers (Supplementary Table S2) and cDNA derived from mature (dry) seed mRNA serving as template. Products from the 2 PCRs were then mixed and used as template for a third PCR with primers corresponding to the 5′ and 3′ ends of the full-length AT3G19920.2 ORF along with flanking 5′ and 3′ attB sites. Resulting PCR products were subcloned into pDONR and then pMDC32/ChC using Gateway technology, yielding pMDC32/AT3G19920.2-mCherry. To construct pMDC32/mCherry-VfLDPS and pMDC32/PpLDPS-like-mCherry, encoding N-terminal mCherry-tagged *V. fordii* LDPS and C-terminal mCherry-tagged *P. patens* LDPS-like, respectively, both ORFs were custom-synthesized (IDT) and used as template for PCRs with gene-specific primers that included flanking 5′ and 3′ attB sites (Supplementary Table S2). Resulting PCR products were subcloned into pDONR and then pMDC32/ChN or pMDC32/ChC using Gateway technology, yielding pMDC32/mCherry-VfLDPS and pMDC32/PpLDPS-like-mCherry, respectively. Similarly, pMDC32/Os18CS-mCherry, encoding C-terminal mCherry-tagged *O. sativa* 18CS, was constructed by subcloning a custom-synthesized DNA fragment (IDT) corresponding to the *O. sativa* 18CS ORF, along with flanking 5′ and 3′ attB sites, directly into pMDC32/ChC using Gateway technology. pMDC32/At18CS-mCherry and pMDC32/AT3G50780-mCherry encoding C-terminal mCherry-tagged Arabidopsis 18CS and the Arabidopsis BTB/POZ domain-containing protein AT3G50780, respectively, were constructed by PCR-amplifying each ORF using Arabidopsis cDNA (derived from mRNA isolated from rosette leaves of 4-wk-old plants) as template DNA and gene-specific primers with flanking 5′ and 3′ attB sites (Supplementary Table S2). Resulting PCR products were subcloned using Gateway technology into pDONR, generating pDONR/*At*18CS and pDONR/AT3G50780, and then pMDC32/ChC, yielding pMDC32/*At*18CS-mCherry and pMDC32/AT3G50780-mCherry.

pBEE401E/LDPSsgRNA was constructed for CRISPR/Cas9-based genome editing of Arabidopsis *LDPS* using the methods described by Wang et al. (2015). Briefly, primers were synthesized that corresponded to sgRNAs that were designed using CRISPR-PLANT v2 (http://www.genome.arizona.edu/crispr2/) (Minkenberg et al. 2019) to specifically target regions in the *LDPS* ORF (Supplementary Table S2); refer also to Supplementary Fig. S8 for the relative positions of the sgRNA and the corresponding 405-nucleotide-long region in the *LDPS* gene that was removed by CRISPR/Cas9-based genome editing. Primers also contained sequences complementary to a region in the pCBC-DT_1_T_2_ template plasmid (Wang et al. 2015) containing the sgRNA scaffold, promoters, and terminators. Resulting PCR products were digested with *Bsa*I, gel-purified, and then ligated into similarly digested pBEE401E (Wang et al. 2015), yielding pBEE401E/LDPSsgRNA.

Plasmids encoding the various C-terminal mCherry-tagged truncation mutants of LDPS (see Fig. 2) were generated by PCR-amplifying selected portions of the *LDPS* ORF (without a stop codon) using pMDC32/LDPS (see above) as template DNA and gene-specific primers that also included flanking attB sites (Supplementary Table S2). All 5′ primers also introduced a translation initiation codon (i.e. ATG), except for the 5′ primer used to PCR-amplify the sequence corresponding to amino acid residues 1 to 216 in LDPS (i.e. LDPS^1-216^), which included the native initiation codon in the *LDPS* ORF. Resulting PCR products were subcloned using Gateway technology into pDONR and then pMDC32/ChC, which resulted in a C-terminal mCherry-tag appended to each LDPS truncation mutant (e.g. pMDC32/LDPS^1-216^-mCherry, pMDC32/LDPS^210-416^-mCherry, etc.). Similarly, pMDC32/18CS^150-258^-mCherry, encoding a translation initiation methionine (ATG), amino acid residues 150 to 258 in Arabidopsis 18CS, and a C-terminal-appended mCherry-tag, was constructed by PCR-amplifying the corresponding sequence in the *18CS* ORF using pMDC32/*At*18CS-mCherry (see above) as template DNA and gene-specific primers with flanking 5′ and 3′ attB sites (Supplementary Table S2). PCR products were subcloned into pDONR and then pMDC32/ChC using Gateway technology. pMDC32/LDPS^Δ209-227^-mCherry, which encodes a mutant version of Arabidopsis LDPS lacking residues 209 to 227, was constructed by PCR-amplifying the portions of the *LDPS* ORF upstream and downstream of the deleted sequence using 2 pairs of overlapping gene-specific primers (Supplementary Table S2) and pMDC32/ChC/LDPS as template DNA. Products from the 2 PCRs were then mixed and used as template for a third PCR with primers corresponding to the 5′ and 3′ ends of the *LDPS* ORF along with flanking attB sites (Supplementary Table S2). Resulting PCR products were subcloned into pDONR and then pMDC32/ChC using Gateway technology, yielding pMDC32/LDPS^Δ209-227^-mCherry. Multiple PCRs were also used to construct pMDC32/18CS^167-202D191-242^-mCherry, which encodes a modified version of C-terminal mCherry-tagged Arabidopsis 18CS, whereby amino acid residues 167 to 202 in Arabidopsis 18CS were replaced with the corresponding residues (i.e. 191 to 242) in Arabidopsis LDPS. Specifically, the portions of the *18CS* ORF upstream and downstream of the sequence encoding residues 167 to 202 and the corresponding sequence corresponding in LDPS (i.e. encoding residues 191 to 242) were amplified in 3 separate PCRs with gene-specific primers (Supplementary Table S2). Products from the 3 PCRs were then mixed and used as template for a fourth PCR with primers corresponding to the 5′ and 3′ ends of the *18CS* ORF along with attB sites (Supplementary Table S2). Resulting PCR products were subcloned into pDONR and then pMDC32/ChC using Gateway technology, yielding pMDC32/18CS^167-202D191-242^-mCherry. pMDC32/LDPS^170-307 YYLILDV5^-mCherry, pMDC32/LDPS^170-307YYLILΔE5^-mCherry, and pMDC32/LDPS^170-307PΔA^-mCherry, encoding amino acid residues 170 to 307 in Arabidopsis LDPS with selected mutations (as indicated by the construct name; refer to Fig. 4A for additional details on the specific amino acids modified in each construct), along with a translation initiation methionine (Met) and C-terminal mCherry-tag, were all constructed in a similar manner. Specifically, the corresponding DNA fragments for each mutant, along with flanking 5′ and 3′ attB sites, were custom-synthesized (IDT) (Supplementary Table S2) and subcloned directly into pMDC32/ChC using Gateway technology. For Y2H screening with LDPS, the Arabidopsis *LDPS* ORF was amplified by PCR using appropriate primers (Supplementary Table S2), and then, PCR products were digested with *Eco*RI and *Bam*HI and subcloned into similarly digested pGBKT7-DNA-BD (Clontech), yielding the “bait” vector pGBKT7/LDPS.

pMDC32/mGFP-LDAP3, encoding the monomeric GFP (mGFP) appended to the N terminus of Arabidopsis LDAP3, was constructed by PCR-amplifying the full-length ORF of the GFP-LDAP3 fusion protein, using pRTL2/mGFP-LDAP3 (Gidda et al. 2016) as template DNA and gene-specific primers with flanking attB sites (Supplementary Table S2). Resulting PCR products were subcloned into pDONR and then pMDC32 using Gateway technology. pMDC32/OLEO1, encoding nontagged Arabidopsis OLEO1, was constructed by PCR-amplifying the ORF of *OLEO1* using pMDC84/OLEO1-mGFP (Pyc et al. 2017) as template DNA and gene-specific primers with flanking attB sites and then subcloning into pDONR and pMDC32 using Gateway technology. pMDC32/OLEO1^1-43^-mGFP and pMDC32/mGFP-OLEO1^116-173^, encoding amino acid residues 1 to 43 and 116 to 173 of Arabidopsis OLEO1 and a C- or N-terminal-appended mGFP tag, respectively, were constructed by PCR-amplifying each region in the *OLEO1* ORF with the appropriate gene-specific primers and flanking attB sites (Supplementary Table S2) and pMDC84/OLEO1-mGFP (see above) as template DNA. PCR products were subcloned (via Gateway technology) into pDONR and then either pMDC32/NGFP (Krawczyk et al. 2022b) or pMDC32/CGFP, yielding pMDC32/mGFP-OLEO1^116-173^ and pMDC32/OLEO1^1-43^-mGFP, respectively. The pMDC32/CGFP binary vector contains a Gateway recombination site followed by the full-length ORF of *mGFP*, which provides for the expression of a fusion protein with a C-terminal-appended mGFP. To construct pMDC32/CGFP, the *mGFP* ORF was amplified from pRTL2/MCS-mGFP (Clark et al. 2009) using gene-specific primers that also included 5′ *Pac*I and 3′ *Sac*I restriction sites (Supplementary Table S2). The resulting PCR products were digested with *Pac*I and *Sac*I and ligated into similarly digested pMDC32/ChN (Doner et al. 2021), yielding pMDC32/CGFP.

The construction of other plant expression binary plasmids used in this study have been described elsewhere, including: pORE04/P19, encoding the TBSV RNA-silencing suppressor P19, and pORE04/LEC2, encoding the regulator of seed development in Arabidopsis LEC2 (Petrie et al. 2010); pBIN/ER-GK (GFP-ER), encoding ER lumen-localized GFP (obtained from the ABRC; Nelson et al. 2007); pMDC32/mCherry, encoding mCherry alone (Pyc et al. 2017); pMDC32/LDAP3-mCherry, encoding C-terminal mCherry-tagged Arabidopsis LDAP3 (Gidda et al. 2016); pMDC32/GFP-SEIPIN2, encoding N-terminal mGFP-tagged Arabidopsis SEIPIN2 (Cai et al. 2015); pMDC32/VAP27-1-GFP, encoding C-terminal mGFP-tagged Arabidopsis VAP27-1 (Greer et al. 2020); and pMDC32/FSP27, encoding mouse (*M. musculus*) FSP27 (Price et al. 2020).

Plasmids used for mbSUS assays were constructed based on 2 Gateway-compatible yeast expression vectors: (i) pMetYC-Dest, which contains the Met-repressible promoter and encodes the C-half of Ub (i.e. Cub) appended to the transcriptional reporter protein complex ProteinA-LexA-VP16, as well as an adjacent 5′ in-frame cloning site, which provides for the expression of a fusion protein with a C-terminal-appended Cub; and (ii) pNX32-Dest, which encodes the N-terminal half of Ub with a point mutation that results in its low affinity for Cub (i.e. Nub32), as well as a 3′ in-frame cloning site, which provides for the expression of a fusion protein with an N-terminal-appended Nub32 (Grefen et al. 2009). Specifically, the ORF of Arabidopsis *LDPS* (without its stop codon), which served as “bait” in mbSUS assays (see “mbSUS assays” for additional details), was subcloned using Gateway technology from pDONR/LDPSnostop (Kretzschmar et al. 2020) into pMetYC-Dest, yielding pMETYC-Dest/LDPS-Cub. Similarly, the ORFs of the various Arabidopsis “prey” proteins examined in this study (i.e. SEIPIN1-3, LDIP, LDAP1-3, VAP27-1, OLEO1 and 2, and LDPS) were PCR-amplified using gene-specific primers and flanking attB sites (Supplementary Table S2), along with the appropriate plasmids as templates. The resulting PCR products were subcloned using Gateway technology into pDONR and then pNX32-Dest. pNubWtXgate, encoding WT Nub, which is a high affinity for Cub (Grefen et al. 2009), and pNX32-Dest, encoding “empty” Nub32 (Grefen et al. 2009), were used as positive and negative controls, respectively.

### Microscopy

Arabidopsis seeds and seedlings and *N. benthamiana*-infiltrated leaves were processed for CLSM imaging, including staining LDs, either with the neutral lipid-specific fluorescent dye BODIPY 493/503 (Listenberger et al. 2007) or monodansylpentane (MDH) (Yang et al. 2012b), as previously described (Cai et al. 2015; Gidda et al. 2016); see also Greer et al. (2020) for additional information on processing Arabidopsis mature (dry) seeds for CLSM. For developing Arabidopsis seeds, seeds were collected from ∼15- to 20 mm-long green siliques at 10 to 12 d after flowering, when embryos are at the “bent” stage of development (Le et al. 2010). Seeds were removed from the silique with a scalpel, fixed for 20 min in 4% (v/v) formaldehyde in 50 mm piperazine-*N,N*′-bis(2-ethanesulfonic acid) (PIPES) buffer (pH 7.0), washed 3 times with PIPES buffer, subsequently stained with 1 *μ*g/mL BODIPY (in PIPES buffer), mounted on a glass slide, and imaged using CLSM. For Arabidopsis pollen grains, pollen was collected from ∼10 flowers (∼3 h after the beginning of the light cycle) of 5-to-6-wk-old plants in an Eppendorf tube with 1 mL of PIPES buffer (see above). Pollen was then vortexed for 30 s 2 to 3 times and centrifuged at room temperature for 7 min at 3,200 × *g*; supernatant was removed and subsequently resuspended in 50 *μ*L PIPES buffer, 18% (w/v) sucrose, and 2 *μ*g/mL BODIPY, mounted on a glass slide, and imaged using CLSM.

CLSM imaging of plant cells was carried out with a Leica SP5 CLSM equipped with a 63× glycerol immersion objective (NA = 1.3) and 5 laser systems, including an argon (Ar)-ion laser, green, orange, and red helium–neon (HeNe) lasers, and a Radius 405-nm laser (Leica Microsystems). Alternatively, for quantifications of LD sizes (diameters) in mature (dry) Arabidopsis seeds, imaging was performed using a Zeiss LSM710 equipped with a 63× water immersion lens (NA = 1.15), an Ar-ion laser, and an Airyscan 2 area detector (Carl Zeiss Inc). BODIPY and GFP were excited with an Ar-ion laser at 488 nm and detected at a bandwidth of 500 to 540 nm, MDH was excited with the Radius 405-nm laser and detected at a bandwidth of 420 to 480 nm, and mCherry was excited with an HeNe laser at 543 nm and detected at a bandwidth of 590 to 640 nm, and gain and offset settings varied depending on the sample. All images of plant cells were acquired as single optical sections (i.e. z-sections) or as z-stacks (consisting of 0.4-μm z-sections, 10 *μ*m in total) and, depending on the CLSM system, saved as either 512 × 512- or 1,024 × 1,024-pixel digital micrographs. Excitation and emission signals for fluorescent proteins and neutral lipid-specific dyes were collected sequentially in double- or triple-labelling experiments and are the same as those described previously (Cai et al. 2015; Gidda et al. 2016); single-labeling experiments showed no detectable crosstalk between channels at the settings used for data collection. All images of plant cells shown in individual figures are representative of at least 3 separate experiments, including at least 8 separate Arabidopsis seeds, seedlings, or pollen grains, whereby at least 24 areas (i.e. micrographs) were analyzed, and at least 3 separate infiltrations of *N. benthamiana* leaves, whereby ≥24 transformed leaf areas were analyzed. The numbers and sizes (diameters) of LDs in images of Arabidopsis seeds and seedlings (with the exception of Arabidopsis 15-d-old seedlings, see below) and *N. benthamiana* leaves were determined using the “Measure” tool in ImageJ (v.1.53e) (https://imagej.nih.gov/ij) (Schneider et al. 2012), whereby the diameters of individual LDs were manually measured from one side along the major axis. Quantification of LD numbers and sizes in 15-d-old Arabidopsis seedlings was performed according to Cai et al. (2015) using the “Analyze Particles” function (using default settings, with the exception of a circularity value of 0.90 to 1.0) in the Fiji-plugin image processing package in ImageJ.

All figure compositions shown in the paper were generated and images therein processed for brightness and contrast using Microsoft PowerPoint (v.16.76.1). Illustrations of gene loci, topology of the OLEO1 protein, and the model for LDPS function were also generated using Microsoft PowerPoint.

### PCR and RT-PCR

Sequence information for all primers used in PCRs for plasmid construction or genotyping Arabidopsis mutant lines, or RT-PCRs to confirm endogenous gene expression in Arabidopsis plants, including disruption in gene expression or overexpression, is available in Supplementary Table S2. gDNA and total RNA were isolated from selected Arabidopsis tissues and organs as described elsewhere (Doner et al. 2021), except for seeds, whereby total RNA was isolated according to a protocol adapted from Meng and Feldman (2010). Specifically, mature (dry) seeds were ground (using a mortar and pestle) into a powder in liquid nitrogen and incubated with 1 mL extraction buffer (consisting of 100 mm Tris pH 9.5, 150 mm NaCl, 1% [w/v] sarkosyl, 0.5% [v/v] β-mercaptoethanol) for 5 min. The extract was clarified by centrifugation, and 0.8 mL supernatant was subsequently mixed with 0.4 mL 100% chloroform and then 0.4 mL acid phenol:chloroform (pH 4.5) (Thermo Fisher Scientific) and then centrifuged, and the resulting aqueous phase was combined with 0.06 mL 3 m sodium acetate (pH 5.2) and 0.6 mL 100% isopropanol. After a 10-min incubation, the sample was centrifuged and the resulting RNA pellet was washed with 70% (v/v) ethanol, dried, and then subjected to purification using TRIzol reagent, following the manufacturer's protocol (Invitrogen). Complementary DNA (cDNA) was synthesized using 750 *μ*g of total RNA and qScript cDNA SuperMix, according to the manufacturer's instructions (Quanta Biosciences). All RNAs (cDNAs), including for the reference gene Arabidopsis *β-TUBULIN isoform 4* (*TUB4*), were amplified by 30 cycles at 94 °C for 30 s, 55 °C for 45 s, and 72 °C for 90 s, using the appropriate gene-specific primers (Supplementary Table S2). PCR products (and RT-PCR products, see below) were separated in agarose gels and imaged using ethidium bromide staining and a gel documentation system.

RT-PCRs used to confirm the expression of transgenes encoding nontagged proteins in *Agrobacterium*-infiltrated N*. benthamiana* leaves were performed according to Cai et al. (2015) with some modifications. Briefly, ∼100 mg of infiltrated leaf tissue was flash frozen in liquid nitrogen in an Eppendorf tube and ground into a fine powder using a metal ball bearing and a Retsch MM301 mixer mill. Total RNA was then isolated using the RNeasy Plant Mini kit (Qiagen) and cDNA was synthesized from 1 *μ*g of RNA using a QuantiTect Reverse Transcription kit. *N. benthamiana ACTIN* served as a reference gene and cycling parameters for all RNAs were 35 cycles at 95 °C for 30 s, 52 °C for 30 s, and 72 °C for 60 s, using the appropriate gene-specific primers (Supplementary Table S2).

### Miscellaneous plant-based assays

Arabidopsis plant height, silique number, seed weight, and seed size were measured as follows. Four replicates of 8 to 9 plants for each line (i.e. WT [Nos-0 and Col-0], *ldps-1*, and *ldps-2*) were grown on soil, and the aboveground vertical height (i.e. distance from the apical meristem to soil surface) and total number of siliques of each plant at 40 and 47 d were determined. Plants were then dried, and all the seeds from each plant were harvested by hand and weighed. Seed size (i.e. area [*x–y*]) was measured according to Herridge et al. (2011), whereby ∼500 seeds from each plant were spread out on a document scanner (Hewlett-Packard) and imaged at high resolution while backlit. Individual seed sizes were quantified using the “Analyze Particles” function using default settings in ImageJ, with the exception of size and circularity values of 0.05 mm to infinity and 0.7 to 1.0, respectively.

To quantify Arabidopsis seed germination, 3 replicates of 40 to 50 seeds from 4 different plants per line (i.e. WT [Nos-0 and Col-0], *ldps-1* and *ldps-2*) were sown on ½ MS plates, stratified at 4 °C in the dark, and then grown at 22 °C in constant light and photographed every hour for 48 h using a Raspberry Pi NoIR (v.2.0) camera. Each image was manually scored for the number of seeds in which the radicle had emerged from the seed coat, serving as the indicator of germination (Bewley 1997), and then, the time point at which 50% of seeds had germinated (i.e. *T*_50_) was calculated. To assess seedling growth rates, 3 replicates of 8 seedlings per line were sown on ½ MS plates, stratified, and germinated as above (i.e. in constant light). Then, 2, 3, 4, 8, and 12 d after the initiation of germination, plates were photographed, and the length of each seedling (i.e. from root tip to the top of the cotyledon) was measured manually using ImageJ. To assess hypocotyl elongation in dark-grown seedlings, seeds were sown on ½ MS plates and wrapped in tinfoil to block light. Seeds were stratified, then placed vertically in a growth chamber at 22 °C for 2, 3, or 4 d, and photographed, and ImageJ was used to manually measure the length of each seedling hypocotyl.

Seed-freezing experiments were carried out according to the procedures described by Shimada et al. (2008). Briefly, 3 replicates of seeds per line were either incubated at −25 °C in the dark for 24 h in a compact upright freezer (Revco Industries) or, as a control, maintained at normal seed storage conditions (i.e. in the dark at room temperature), then both sets of seeds were sown on ½ MS plates with 1% (w/v) sucrose and stratified in the dark for 3 d at 4 °C, and then seeds were either stained with BODIPY and imaged using CLSM (see “Microscopy” for details) or incubated (germinated) at 22 °C in 16-h day/8-h night cycle for 3 d, and then, the seedlings were stained with BODIPY and imaged.

### Proteomics

WT (Nos-0) and *ldps-1* seedlings were grown on ½ MS plates for 2 d as described above (see “Plant material, growth conditions, and transformations”), and mature seeds were rehydrated in water for 30 min. LD-enriched fractions and total cellular fractions from 5 technical replicates were obtained, and then, proteins were isolated, their concentrations determined, and subsequently subjected to in-gel tryptic digestion, as described previously (Kretzschmar et al. 2020); see also Kretzschmar et al. (2020) for details on peptide purification and LC-MS/MS. MS and MS/MS data were processed for feature detection, peptide identification, and protein group assembly using default settings in MaxQuant (v.1.6.2.10) (www.maxquant.org, ABSciex) (Cox and Mann 2008; Cox et al. 2014). The TAIR10 protein database (v.10) (www.arabidopsis.org) (Berardini et al. 2015) was used as a reference. Only proteins identified by at least 2 peptides were considered. Lists of proteins detected by LC-MS/MS are shown in Supplementary Data Sets 1 to 5. Libraries, meta data, raw data files, MaxQuant search files, and ProteinGroup and Peptide search results created by MaxQuant are available also through the ProteomeXchange Consortium via the PRIDE partner repository (https://www.edi.ac.uk/pride/) (Perez-Riverol et al. 2019) under the project accession number identifier PXD041506; refer to metadata in Supplementary Table S3. Perseus (v.1.6.6.2) (Tyanova et al. 2016) was used for data analysis. PCA plots were generated from unfiltered raw values and volcano plots were generated using imputed values, as described previously (Krawczyk et al. 2022b). The imputed protein list is presented in Supplementary Data Set 4, and the significantly enriched proteins are listed in Supplementary Data Set 5.

### Measurement of Arabidopsis seed oil and lipidomics

Total oil content in mature, desiccated Arabidopsis seeds was measured using time-domain, pulsed-field ^1^H-NMR on a minispec mq20 TD-NMR (Bruker Optics). Three replicates of ∼50 mg of seeds per line were used for each measurement, and oil levels (represented as percentage of dry weight) were calculated as described previously (Chapman et al. 2008), but calibrated for Arabidopsis seed oil.

Lipidomics analysis of Arabidopsis mature seeds and 1-, 2-, and 4-d-old dark-grown seedlings was performed using LC-MS/MS. Lipids were extracted from 4 to 5 replicates of 10 mg of lyophilized tissue using isopropanol and chloroform, as described previously (Gidda et al. 2016). Ten microliters of UltimateSPLASH ONE (Avanti Polar Lipids) was added to the samples as an internal standard. LC-MS/MS was performed according to Romsdahl et al. (2022) using 10 *μ*L of lipid extract diluted in 490 *μ*L of acetonitrile/isopropanol/methanol/water (3:3:3:1, v/v/v/v) with 10 mm ammonium hydroxide and 0.1% (v/v) formic acid (for neutral lipid analysis) or 10 *μ*L of extract diluted in 490 *μ*L of 100% (v/v) ethanol with 2 mm ammonium acetate (for polar lipid analysis) and with a Agilent 1290 Infinity II UHPLC coupled to hybrid ABSciex QTRAP 6500^+^ ion trap/triple quadrupole MS. Ionized lipids were collected and analyzed using Analyst (v.1.7) and MultiQuant (v.3.0.3) software (ABSciex).

### mbSUS assays

mbSUS assays were performed using the yeast (*Saccharomyces cerevisiae)* strain THY.AP4 (obtained from the ABRC [CD3-808]) and according to the procedures described by Grefen et al. (2009). Briefly, yeast cells were transformed with selected “bait” and “prey” plasmid pairs using the Frozen-EZ Yeast Transformation II kit (Zymo Research) and then grown at 30 °C on low-selection synthetic complete (SC)_-LWM_ plates, which lacked Leu, Trp, and Met. High-selection media, which lacked Leu, Trp, Met, His, Ade, and Ura (i.e. SC_-LWMHAU_), was purchased from Sunrise Science Products and stock solutions of amino acids (i.e. L-Met and L-His, Sigma-Aldrich; Ura, Research Products International; and Ade, MP Biomedicals) were used to prepare different selection media required for assays, according to Grefen et al. (2009). Selected colonies for each cotransformation were inoculated into liquid SC_-LWM_ and grown overnight at 30 °C and 275 rpm, and then, aliquots of the cultures were pelleted by centrifugation. Thereafter, yeast pellets were either frozen and stored for subsequent western blot analysis (see below) or resuspended at an optical density (OD) of 1.0 or 0.1 OD and spotted onto both SC_-LWM_ and SC_-LWMHAU_ plates that also contained either 5, 50, or 500 *µ*M Met, which allowed for control of the expression of the “bait” (i.e. LDPS-Cub) via its Met-repressible promoter, thereby reducing potential autoactivation and detection of potentially weaker/unstable protein–protein interactions (Obrdlik et al. 2004). In addition, high-selection plates included 100 *μ*g/mL X-Gal (5-bromo-4-chloro-3-indolyl β-D-galactopyranoside, Gold Biotechnology), which provided an additional qualitative measure of protein–protein interaction, based on the blue coloration of yeast due to activation of the β-galactosidase reporter gene (Schneider et al. 1996). Yeast cells were cultivated at 30 °C for 2 to 5 d and then analyzed. The results of growth assays shown are representative of those obtained from analyzing 5 isolated yeast colonies from at least 3 independent cotransformations.

Expression of mbSUS fusion proteins was confirmed using western blotting (refer to Supplementary Fig. S18). Briefly, frozen cell pellets were reconstituted in “Lyse and Load” buffer (consisting of 50 mm Tris–HCl pH 6.8, 4% [w/v] SDS, 8 m urea, 30% [v/v] glycerol, 0.1 m dithiothreitol, and 0.005% [w/v] bromophenol blue) and then vortexed in the presence of acid-washed glass beads (0.4 to 0.6 *µ*m, Sigma-Aldrich) according to Grefen et al. (2009). Proteins were separated using 10% TGX Stain-Free FastCast polyacrylamide gel (Bio-Rad Laboratories) and electroblotted onto PVDF membrane (Bio-Rad Laboratories) using a Trans-blot Turbo System (Bio-Rad Laboratories). Membranes were incubated with either rabbit α-VP16-tag (Abcam, cat. no. ab4808) or mouse α-hemagglutinin (HA)-tag (Abcam, cat. no. ab1424) primary antibodies, to detect “bait” and “prey” proteins, respectively, and then incubated with the corresponding antirabbit (Fisher Scientific, no. G-21234) or antimouse (Abcam, cat. no. ab205719) secondary antibodies. Immunoreactive proteins were visualized using Clarity Western ECL substrate and a ChemiDoc Imaging System (Bio-Rad Laboratories).

### Y2H screening

Screening of a Y2H library, consisting of Arabidopsis cDNA from various plant tissues and cloned into the appropriate prey vector (Takara Bio Inc), using Arabidopsis LDPS (pGBKT7/LDPS; see “Plasmid Construction” for details) as “bait,” was carried out with the Matchmaker Gold Y2H System (Takara Bio Inc), as described by the manufacturer and as we have done so in previously published Y2H screens (Park et al. 2013; Pyc et al. 2017; Doner et al. 2021). All yeast strains that grew at 30 °C on selective synthetic dextrose plates, which lacked Trp and Leu, but contained X-Gal and aureobasidin A (Takara Bio USA Inc.), were designated as either “strong,” “moderate,” or “weak” interactors based on the relative growth and color of the colony, the latter of which corresponds to the activation of the *MEL1* reporter gene. Plasmids were extracted from yeast cells to determine the identity of encoded candidate LDPS-interacting (prey) proteins (listed in Supplementary Table S1) by automated DNA sequencing.

### Bioinformatics

Protein sequences of LDPS and its homologs were obtained from TAIR or the Phytozome (v.13) database (https://phytozome-next.jgi.doe.gov) (Goodstein et al. 2012). LDPS, 18CS, and BTB/POZ domain protein family homologs in Arabidopsis were determined using the PANTHER domain database (v.17.0) (www.pantherdb.org) (Thomas et al. 2022), and LDPS, LDPS-like, and 18CS homologs from other species were identified using the “Protein Homologs” tool at Phytozome, except for sequences of *V. fordii* (tung tree) LDPS and 18CS homologs, which were obtained from the *V. fordii* transcriptome (Cui et al. 2018). Redundant and shortened (i.e. truncated) sequences were removed, and remaining sequences were aligned using MUSCLE (Multiple Sequence Comparison by Log-Expectation) (https://www.ebi.ac.uk/Tools/msa/muscle/) (Edgar 2004) and, for Supplementary Fig. S5, visualized using pyBoxshade (https://github.com/mdbaron42/pyBoxshade). Phylogenetic trees were generated using the maximum likelihood method of MEGA X (v.11.0.9) (www.megasoftware.net) (Kumar et al. 2018); refer to Supplementary Data Sets 6 to 9 for alignments in FASTA format and Newick files corresponding to phylogenetic trees presented in Fig. 1B and Supplementary Fig. S1. Alternatively, for the LDPS homologs in Arabidopsis (see Supplementary Fig. S2B), as well as LDPS and FSP27, sequences were aligned using the Clustal Omega multiple sequence alignment tool at the European Molecular Biology Laboratory-European Bioinformatics Institute (https://www.ebi.ac.uk/jdispatcher/msa/clustalo) (Madeira et al. 2024); refer to Supplementary Data Sets 10 and 11 for corresponding alignments in FASTA format and Newick files. Root-mean-square deviation (RMSD) values were calculated by comparing each AlphaFold model to that of Arabidopsis LDPS using ChimeraX Matchmaker (v.1.2.5) (www.cgl.ucsf.edu/chimerax/) (Pettersen et al. 2021); in all cases, at least 204 amino acid pairs were used for the RMSD calculation (see Supplementary Table S4 for details). Putative BTB/POZ domains (InterPro Domain IPR000210) in proteins were assessed also based on the InterPro database (www.ebi.ac.uk/interpro/) (Paysan-Lafosse et al. 2023). Microarray-based data for Arabidopsis *LDPS, 18CS*, and *OLEO1* gene expression were obtained from the “Arabidopsis eFP Browser” tool (v.2.0) hosted at BAR (https://bar.utoronto.ca/efp/cgi-bin/efpWeb.cgi) (Winter et al. 2007), and a heat map was generated using Microsoft Excel (v.16.17).

Structural models of selected proteins, including Arabidopsis LDPS and 18CS, were downloaded (in December 2022 or, for the predicted LDPS dimer, in June 2024) from the AlphaFold Protein Structure Database (www.alphafold.ebi.ac.uk) (Jumper et al. 2021; Varadi et al. 2022), visualized in ChimeraX (v.1.2.5) (www.cgl.ucsf.edu/chimerax/) (Pettersen et al. 2021) (or for the predicted LDPS dimer using PyMOL, v.2.5.7, https://www.pymol.org), and aligned to Arabidopsis LDPS using the “Matchmaker” tool. Protein models shown in Figs. 3 and 4 and Supplementary Fig. S6 were colored according to their predicted local difference distant test (pLDDT) score, which is a confidence measure of model quality, with blue representing low model quality and red representing high model quality. The N-terminal regions of LDPS and 18CS (residues 1 to 114 and 1 to 96, respectively; refer to Fig. 3A and Supplementary Fig. S6) were not included in structural models, since the structures in these regions were predicted to be disordered, albeit with too low of confidence. Helical wheels used to visualize potential amphipathic α-helices and corresponding hydrophobicity and hydrophobic moment scores for each helix were generated using HeliQuest (v.2.0) (https://heliquest.ipmc.cnrs.fr/cgi-bin/ComputParams.py) (Gautier et al. 2008).

### Statistical analysis

Statistical analyses of Arabidopsis plant height, silique number, seed weight, size and oil content, LD number and size (with the exception of seed/seedling freezing experiments, see below), lipidomics data, and hypocotyl length were all performed using a two-tailed Student's *t* test with Microsoft Excel (v.16.17). For LD number and/or size quantification in *N. benthamiana* leaves and Arabidopsis seedlings in seed-freezing experiments, a one-way ANOVA test followed by Tukey's post hoc multiple comparison test was performed using either R (v.4.2.1) (www.r-project.org) or Prism (v.8) (GraphPad; www/graphpad.com). Statistical analyses of proteomics data were performed using Perseus (v.1.6.6.2) (https://maxquant.net/perseus/) (Tyanova et al. 2016). Statistical analyses used for phylogenetic tree constructions were performed using MEGA-X (v.11.0.9) (www.megasoftware.net) (Kumar et al. 2018). Summaries of all statistical analysis data are available in Supplementary Table S4.

### Accession numbers

Accession numbers, based on TAIR, NCBI (National Center for Biotechnology Information) (www.ncbi.nlm.nih.gov), and/or the AlphaFold Protein Structure Database, for the proteins examined in this study are as follows: Arabidopsis BTB/POZ domain protein (AT3G50780), 18CS (AT5G64230; F4KDK4), EF1α (AT2G39990), LDAP1 (AT1G67360), LDAP2 (AT2G47780), LDAP3 (AT3G05500), LDIP (AT5G16550), LDPS (AT3G19920; Q8GX27), LEC2 (AT1G28300), OLEO1 (AT4G25140), OLEO2 (AT5G40420), PUX10 (AT4G10790), SEIPIN1 (AT5G16460), SEIPIN2 (AT1G29760), SEIPIN3 (AT2G34380), and TUB4 (AT5G44340), VAP27-1 (AT3G60600); *Brachypodium distachyon* LDPS (I1IF23); *Brassica rapa* 18CS (A0A398AGM7) and LDPS (A0A397ZIN7); *Eucalyptus grandis* 18CS (A0A059AU93) and LDPS (A0A059AB42); *M. musculus* FSP27 (NP_848460.1); *Marchantia polymorpha* LDPS (A0A2R6WKF5); *N. benthamiana* ACTIN (AY179605.1); *O. sativa* 18CS (EEE57829.1) and LDPS (Q6Z696); *P. patens* LDPS-like (XP_024403883.1; A0A2K1L103); *Solanum lycopersicum* 18CS (A0A3Q7EQU7) and LDPS (A0A3Q7EVG0); TBSV P19 (CAC01278.1); and *Vitis vinifera* 18CS (F6HRD6) and LDPS (F6HIQ3). The accession numbers for tung tree (*V. fordii*) LDPS (tung.mrna.scaffold101.00003) and 18CS (tung.mrna.scaffold1156.00010.001) are based on the *V. fordii* transcriptome (Cui et al. 2018), available at the National Genomics Data Center, Chinese National Genomics Data Center for Bioinformation (https://ngdc.cncb.ac.cn). The accession numbers (and deduced amino acid sequences) for all the other LDPS, LDPS-like, and 18CS protein homologs shown in phylogenetic trees are listed in Supplementary Data Sets 6 to 11.

## Supplementary Material

koaf121_Supplementary_Data

## Data Availability

All the data acquired from this study are included in the main text and Supplementary information. Proteomics data has also been deposited at the ProteomeXChange Consortium via the PRIDE partner repository (Accession number identifier PXD041506).

## Supplementary data

The following materials are available in the online version of this article.

**Supplementary Figure S1.** Phylogenetic analysis of LDPS, LDPS-like and 18CS homologs in the predicted proteomes of plant species currently available at the Phytozome database.

**Supplementary Figure S2.** Phylogenetic analysis of the LDPS homologs in Arabidopsis and intracellular localization of Arabidopsis LDPS and its BTB/POZ domain-containing protein homolog AT3G50780 in *N. benthamiana* leaf cells.

**Supplementary Figure S3.** Expression of LDPS isoforms AT3G19920.1 and AT3G19920.2 and homologs in Arabidopsis and intracellular localization of AT3G19920.2 in *N. benthamiana* leaf cells.

**Supplementary Figure S4.** Intracellular localization of Arabidopsis 18CS^150-258^-mCherry in *N. benthamiana* leaf cells.

**Supplementary Figure S5.** Alignment and Boxshade analysis of deduced polypeptide sequences of LDPS, LDPS-like, and 18CS proteins from various plant species.

**Supplementary Figure S6.** 3D structures of LDPS, LDPS-like, and 18CS proteins from various plant species, as predicted by AlphaFold.

**Supplementary Figure S7.** Helical wheel projections of the AlphaFold-predicted α-helix in the “variable” region in LDPS, LDPS-like, and 18CS proteins from various plant species.

**Supplementary Figure S8.** Characterization of Arabidopsis *ldps-1* and *ldps-2* mutant plant lines.

**Supplementary Figure S9.** LD sizes in seeds, seedlings, leaves and pollen grains of Arabidopsis WT and *ldps-2* plant lines.

**Supplementary Figure S10.** Complementation of the small LD phenotype in the Arabidopsis *ldps-2* mutant line by constitutive overexpression of *LDPS*.

**Supplementary Figure S11.** Disruption of the *18CS* gene in Arabidopsis and comparison of the resulting LD phenotype with WT, *ldsp-2*, and *pux10* plants.

**Supplementary Figure S12.** Molecular species of TAG derived from Arabidopsis WT and corresponding *ldps* mutant plant lines.

**Supplementary Figure S13.** Molecular species of DAG derived from Arabidopsis WT and corresponding *ldps* mutant plant lines.

**Supplementary Figure S14.** Molecular species of MAG derived from Arabidopsis WT and corresponding *ldps* mutant plant lines.

**Supplementary Figure S15.** Lipidomics analysis of polar lipids in Arabidopsis WT and corresponding *ldps* mutant seeds and seedlings.

**Supplementary Figure S16.** Total abundance of LD coat proteins in LD-enriched proteomes and PCA of total protein groups isolated from total extracts and LD-enriched proteomes from Arabidopsis WT and *ldps-1* seeds and seedlings.

**Supplementary Figure S17.** Characterization of growth and development for Arabidopsis WT and corresponding *ldps* mutant plant lines.

**Supplementary Figure S18.** Western blot analysis of mbSUS fusion proteins expressed in yeast.

**Supplementary Figure S19.** Topology model of Arabidopsis OLEO1 at the LD surface and intracellular localization of OLEO1 truncation mutants in *N. benthamiana* leaf cells.

**Supplementary Figure S20.** Characterization of Arabidopsis plant lines constitutively overexpressing *LDPS*.

**Supplementary Figure S21.** Molecular species of TAG derived from Arabidopsis WT and *LDPS OE* plant lines.

**Supplementary Figure S22.** Molecular species of DAG derived from Arabidopsis WT and *LDPS OE* plant lines.

**Supplementary Figure S23.** RT-PCR analysis of transgene expression in transiently transformed *N. benthamiana* leaves.

**Supplementary Figure S24.** Characterization of Arabidopsis mutant plant lines constitutively overexpressing *LDPS* or disrupted in *LDPS* expression in the *oleo1* mutant background.

**Supplementary Figure S25.** Freezing treatment prior to seed stratification reveals that LDPS and OLEO1 function together to influence LD size in Arabidopsis seedlings.

**Supplementary Figure S26.** Alignment of deduced polypeptide sequences of Arabidopsis LDPS and mouse (*M. musculus*) FSP27.

**Supplementary Figure S27.** 3D protein structure of an Arabidopsis LDPS protein dimer, as predicted by AlphaFold.

**Supplementary Table S1.** List of candidate LDPS-interacting proteins identified by Y2H analysis.

**Supplementary Table S2.** Sequences of oligonucleotides used in plasmid construction, gDNA PCRs, and RT-PCRs.

**Supplementary Table S3.** Metadata file for LC-MS/MS proteomics analysis of Arabidopsis WT and *ldps-1* mutant seeds and seedlings using MaxQuant.

**Supplementary Table S4.** Reports from statistical tests performed in R or GraphPad Prism.

**Supplementary Data Set 1.** LC-MS/MS data of known Arabidopsis LD proteins in WT and *ldps-1* seeds and seedlings.

**Supplementary Data Set 2.** Raw quantitative LC-MS/MS proteomics data of Arabidopsis WT and *ldps-1* seeds and seedlings.

**Supplementary Data Set 3.** Normalized and filtered LFQ values and LD enrichment factors from LC-MS/MS proteomic data of Arabidopsis WT and *ldps-1* seeds and seedlings.

**Supplementary Data Set 4**. Imputed LFQ values from LC-MS/MS data of Arabidopsis WT and *ldps-1* seeds and seedlings.

**Supplementary Data Set 5.** Proteins differentially expressed in Arabidopsis WT and *ldps-1* seeds as measured by LC-MS/MS.

**Supplementary Data Set 6.** FASTA-based multiple sequence alignment of LDPS homologs presented in Fig. 1.

**Supplementary Data Set 7.** Newick file of phylogenetic tree presented in Fig. 1.

**Supplementary Data Set 8.** FASTA-based multiple sequence alignment of LDPS homologs presented in Supplementary Fig. S1.

**Supplementary Data Set 9.** Newick file of phylogenetic tree presented in Supplementary Fig. S1.

**Supplementary Data Set 10.** FASTA-based multiple sequence alignment of LDPS homologs presented in Supplementary Fig. S2B.

**Supplementary Data Set 11.** Newick file of phylogenetic tree presented in Supplementary Fig. S2B.

## Dive Curated Terms

The following phenotypic, genotypic, and functional terms are of significance to the work described in this paper:

OLEO1 Gramene: AT4G25140
OLEO1 Araport: AT4G25140

ORF Gramene: Open Reading Frame
ORF Araport: Open Reading Frame

DAG Gramene: Diacylglyercol
DAG Araport: Diacylglyercol

CIDE Gramene: Cell-Death-Inducing DNA Fragmentation Factor-Alpah-Like Effector
CIDE Araport: Cell-Death-Inducing DNA Fragmentation Factor-Alpah-Like Effector

CVMV Gramene: Cassava Vein Mosaic Virus
CVMV Araport: Cassava Vein Mosaic Virus

IDT Gramene: Integrated DNA Technologies
IDT Araport: Integrated DNA Technologies

SDS Gramene: Sodium Dodecyl Sulfate
SDS Araport: Sodium Dodecyl Sulfate

TAIR Gramene: https://www.arabidopsis.org/
TAIR Araport: https://www.arabidopsis.org/

CLO2 Gramene: AT5G55240
CLO2 Araport: AT5G55240

HSD1 Gramene: AT5G50600
HSD1 Araport: AT5G50600

HSD5 Gramene: AT4G10020
HSD5 Araport: AT4G10020

NCBI Gramene: https://www.ncbi.nlm.nih.gov
NCBI Araport: https://www.ncbi.nlm.nih.gov

LDPS Gramene: AT3G19920
LDPS Araport: AT3G19920

PUX10 Gramene: AT4G10790
PUX10 Araport: AT4G10790

pux10-2 Gramene: SALK_139056
pux10-2 Araport: SALK_139056

CLSM Gramene: Confocal Laser-Scanning Microscopy
CLSM Araport: Confocal Laser-Scanning Microscopy

PCR Gramene: Polymerase Chain Reaction
PCR Araport: Polymerase Chain Reaction
