# Supplementary material for: LIPID DROPLET PROTEIN OF SEEDS is involved in the control of lipid droplet size in Arabidopsis seeds and seedlings
Source: Plant Cell. 2025 May 15;37(5):koaf121. doi: 10.1093/plcell/koaf121 (PMC12123417; doi:10.1093/plcell/koaf121)
Supplement: koaf121_Supplementary_Data [file koaf121_supplementary_data.zip › Reference.docx]

**Reference**

Schmid, M., Davison, T. S., Henz, S. R., Pape, U. J., Demar, M., Vingron, M., Schölkopf, B., Weigel, D., and Lohmann, J. U. (2005). A gene expression map of *Arabidopsis thaliana* development. Nat Gen. 37(5), 501–506.
