# Supplementary material for: LIPID DROPLET PROTEIN OF SEEDS is involved in the control of lipid droplet size in Arabidopsis seeds and seedlings
Source: Plant Cell. 2025 May 15;37(5):koaf121. doi: 10.1093/plcell/koaf121 (PMC12123417; doi:10.1093/plcell/koaf121)
Supplement: koaf121_Supplementary_Data [file koaf121_supplementary_data.zip › Supplementary Figures S1-S27 REVISED 2.pdf]

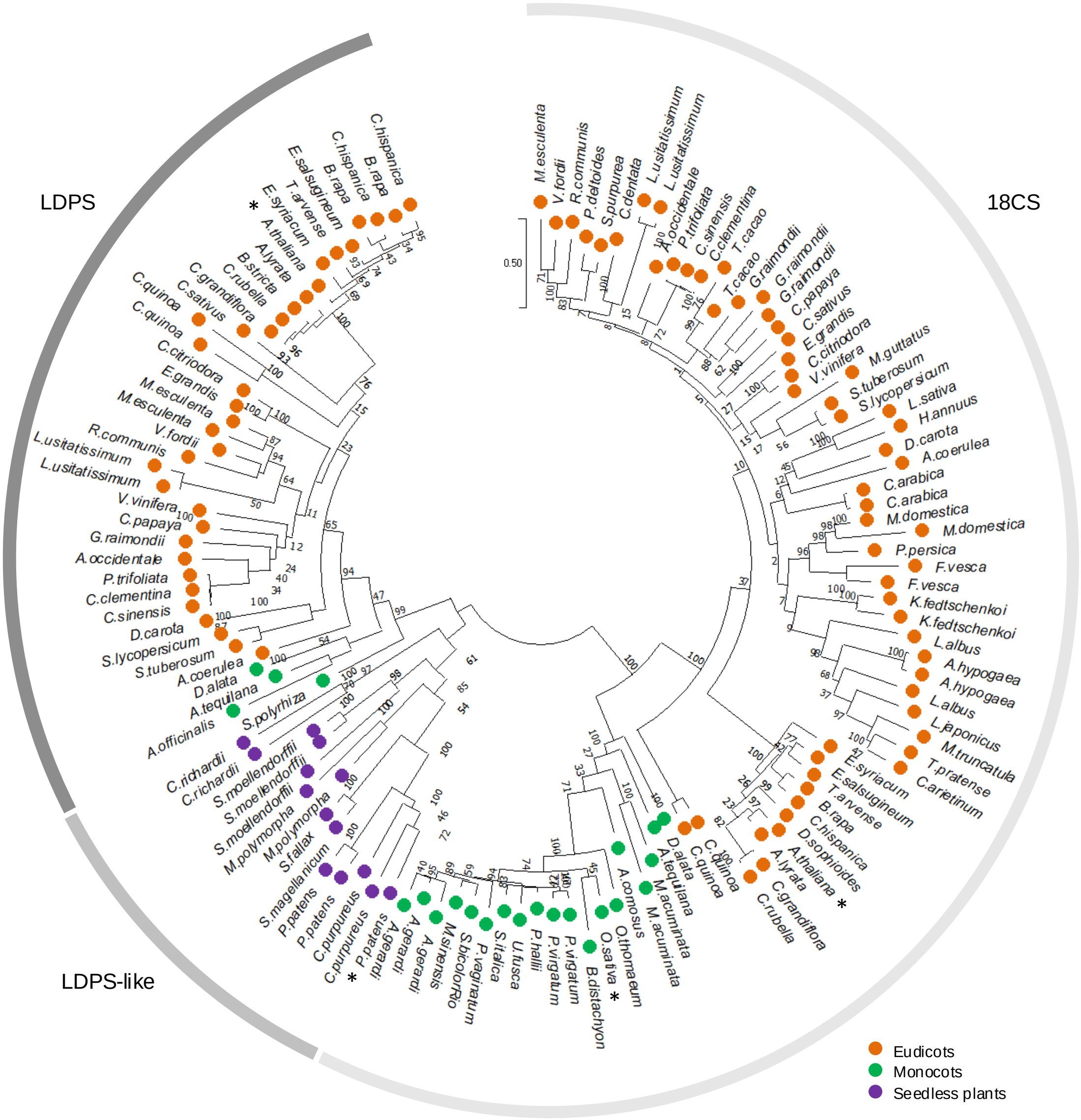


**Supplementary Figure S1**

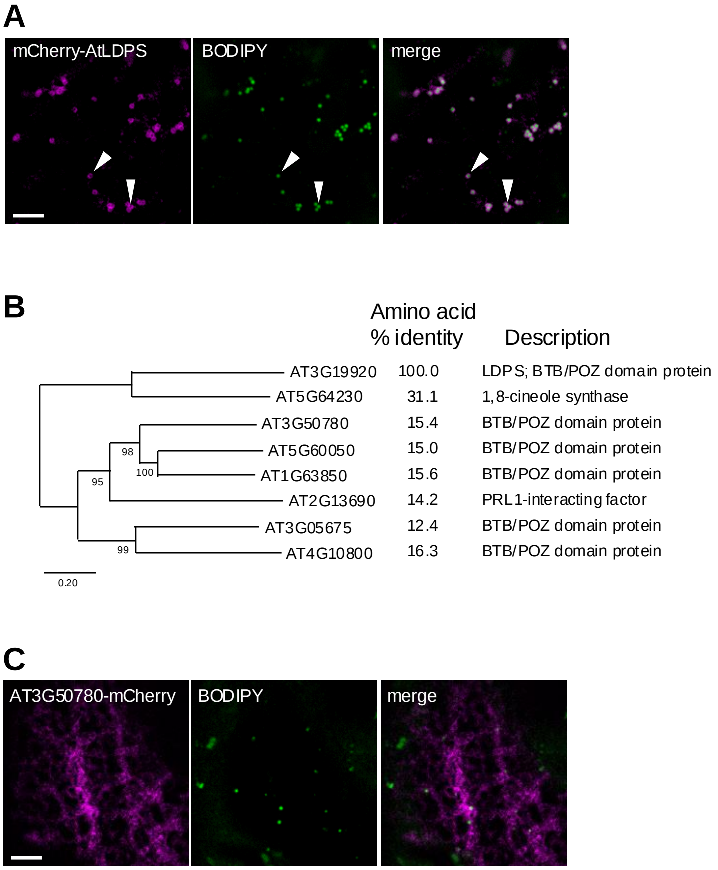


## Supplementary Figure S2

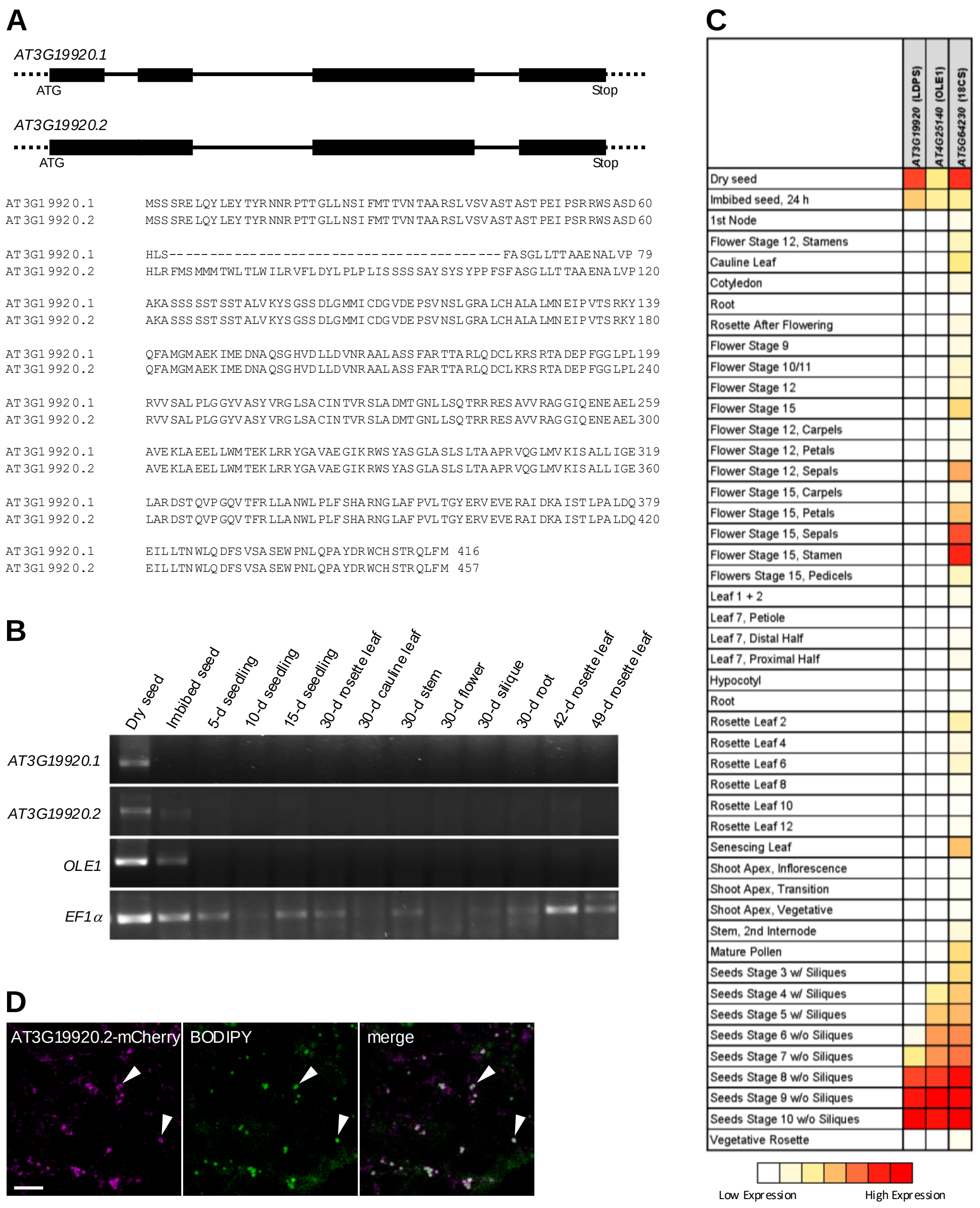


# Supplementary Figure S3

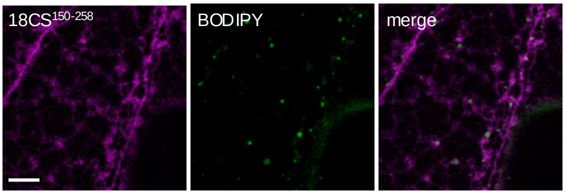


# Supplementary Figure S4

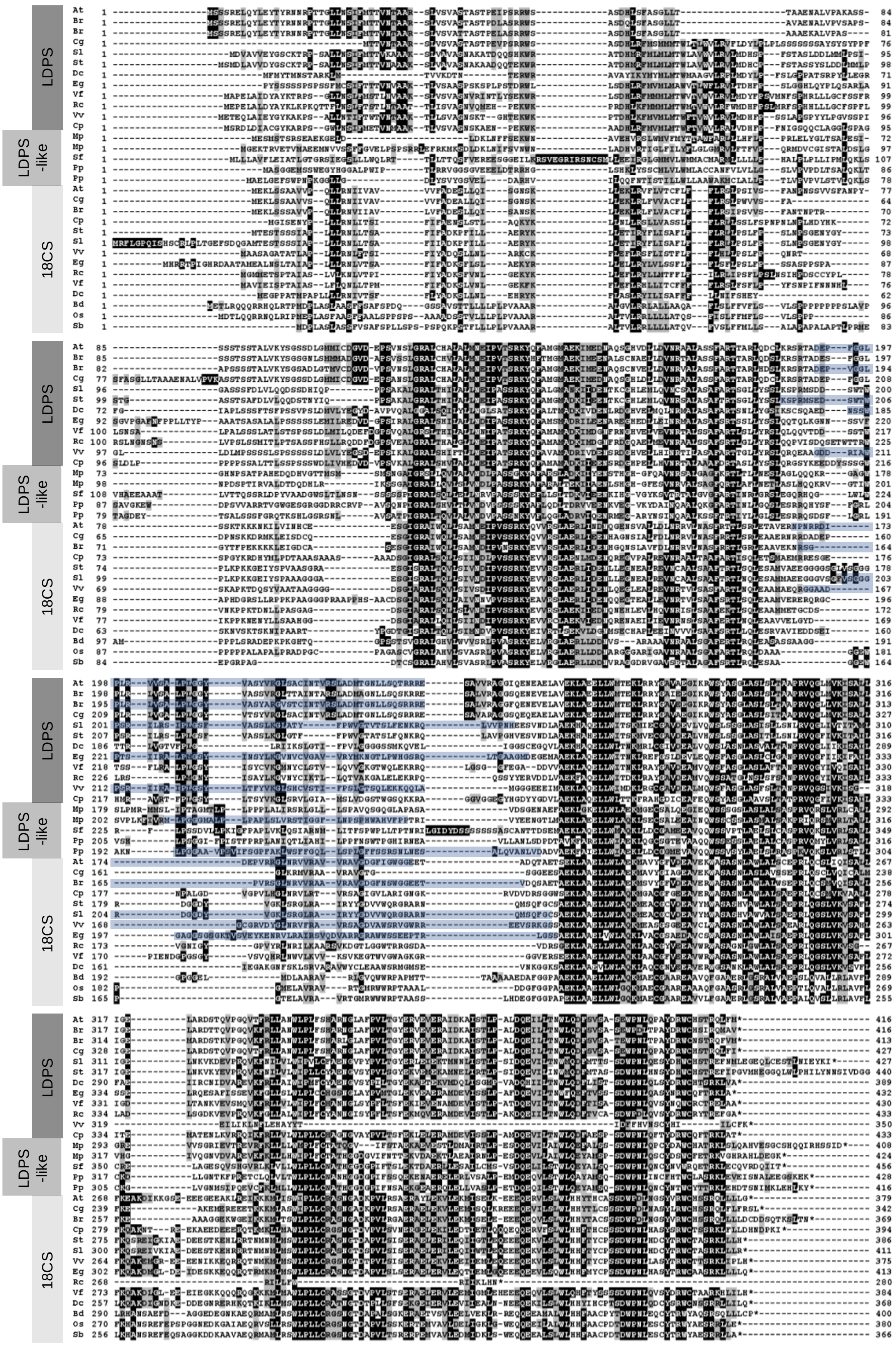


**Supplementary Figure S5**

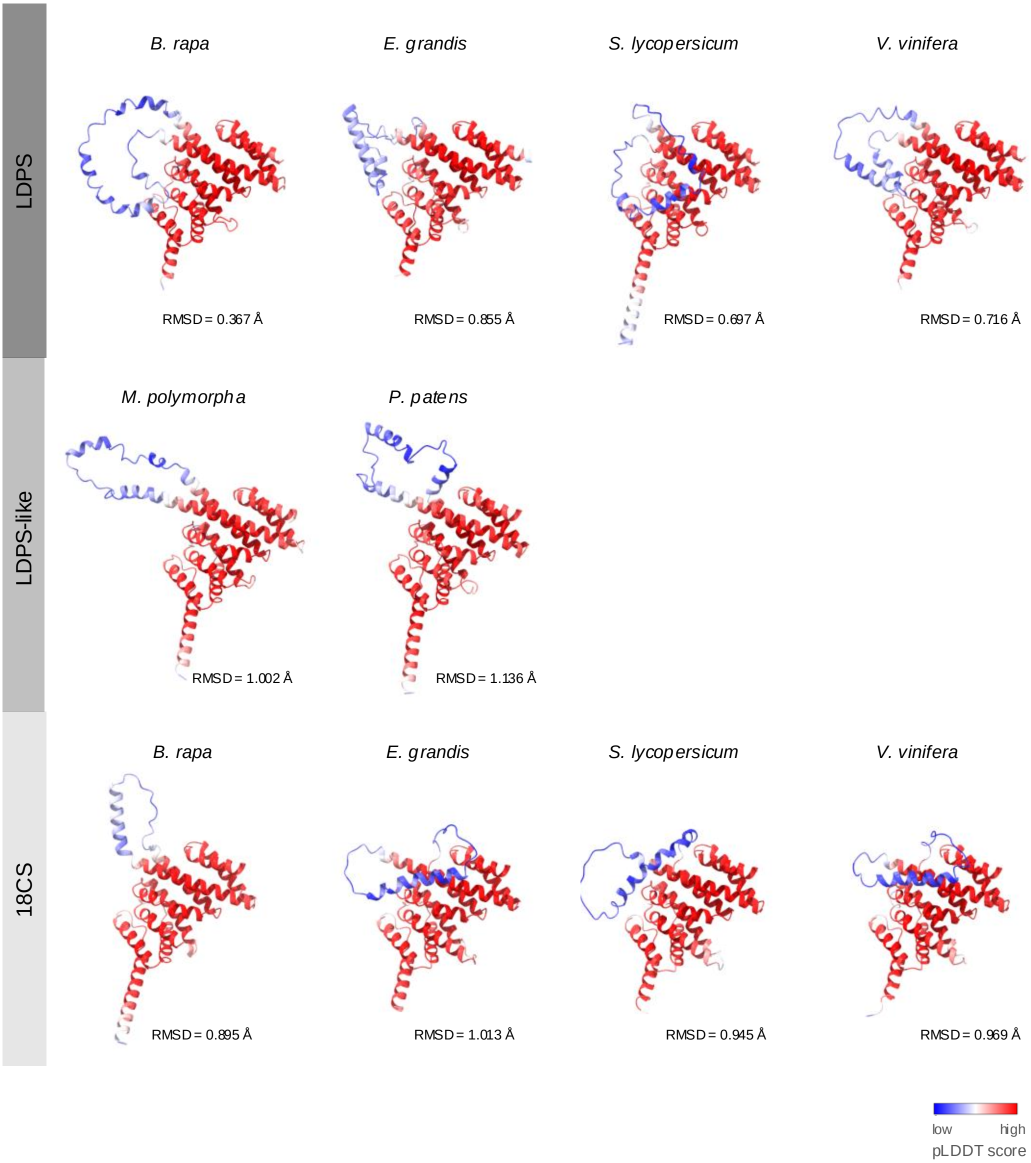


**Supplementary Figure S6**

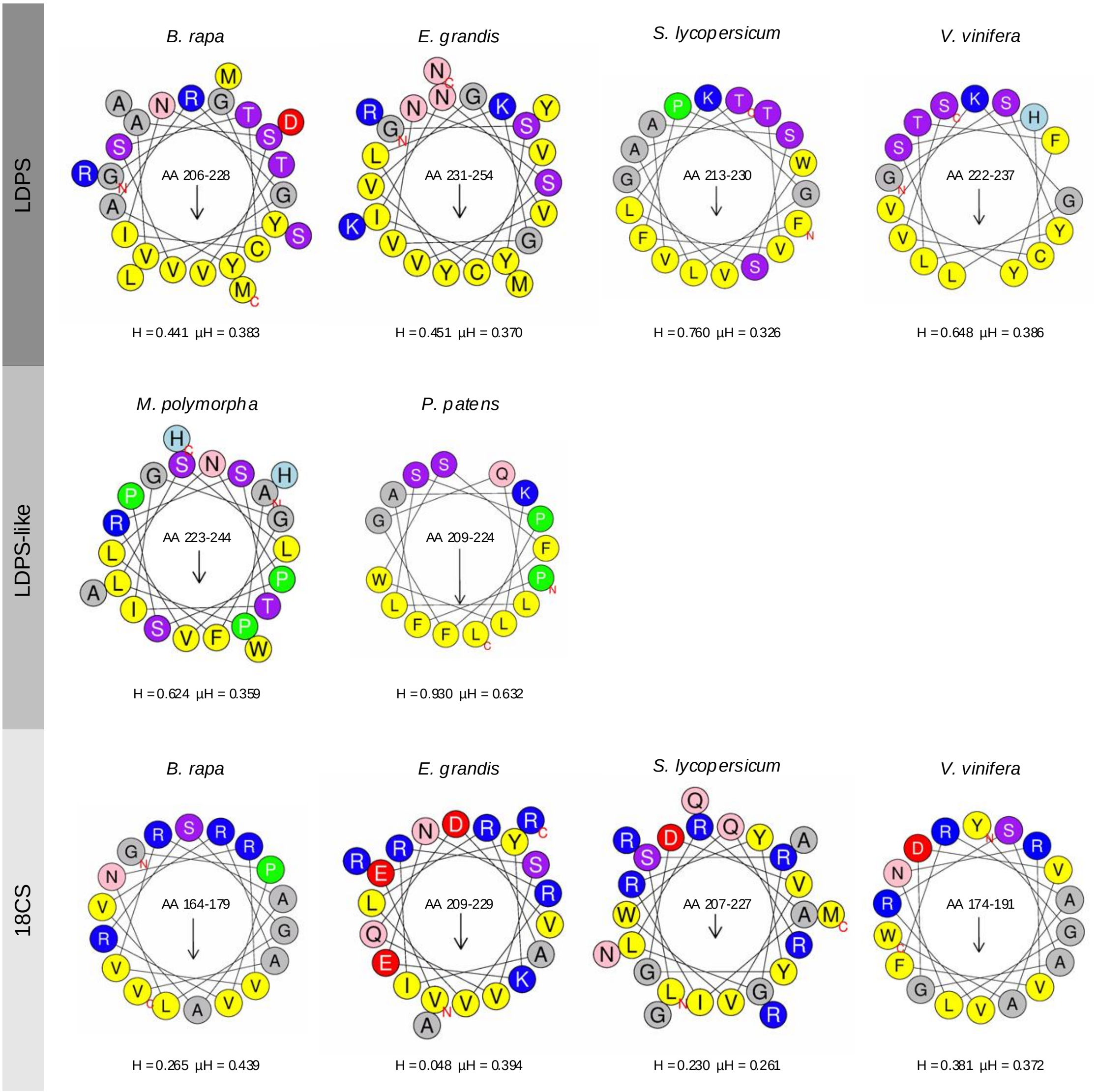


**Supplementary Figure S7**

**A**

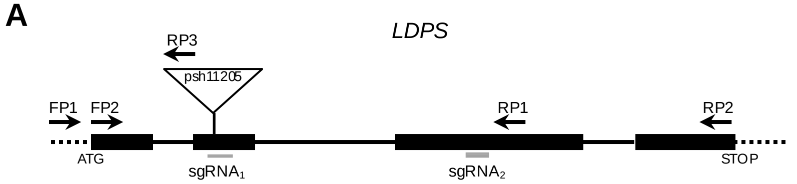


**B**

LDPS (WT) MSSSRELQYLEYTYRNNRPTTGLLNSIFMTTVNTAARSLVSVASTASTPEIPSRRWSASDHLSFASGLLTTAAENALVPAKASSSSSTSS 90
ldps-2 MSSSRELQYLEYTYRNNRPTTGLLNSIFMTTVNTAARSLVSVASTASTPEIPSRRWSASDHLSFASGLLTT------------------- 71

LDPS (WT) TALVKYSGSSDLGMMICDGVDEPSVNSLGRALCHALALMNEIPVTSRKYQFAMGMAEKIMEDNAQSGHVDLLDVNRAALASSFARTTARL 180
ldps-2 ------------------------------------------------------------------------------------------ 71

LDPS (WT) QDCLKRSRTA**DEPFGGLPLRVVSALPLGGYVASYVRGLSACINTVRSLADMTGNLLSQTRRR**ESAVVRAGGIQENEAELAVEKLAEELLW 270
ldps-2 --------------------------VGGYVASYVRGLSACINTVRSLADMTGNLLSQTRRRESAVVRAGGIQENEAELAVEKLAEELLW 135

LDPS (WT) MTEKLRRYGAVAEGIKRWSYASGLASLSLTAAPRVQGLMVKISALLIGELARDSTQVPGQVTFRLLANWLPLFSHARNGLAFPVLTGYER 360
ldps-2 MTEKLRRYGAVAEGIKRWSYASGLASLSLTAAPRVQGLMVKISALLIGELARDSTQVPGQVTFRLLANWLPLFSHARNGLAFPVLTGYER 225

LDPS (WT) VEVERAIDKAISTLPALDQEILLTNWLQDFSVSASEWPNLQPAYDRWCHSTRQLFM 416
ldps-2 VEVERAIDKAISTLPALDQEILLTNWLQDFSVSASEWPNLQPAYDRWCHSTRQLFM 281

**C**

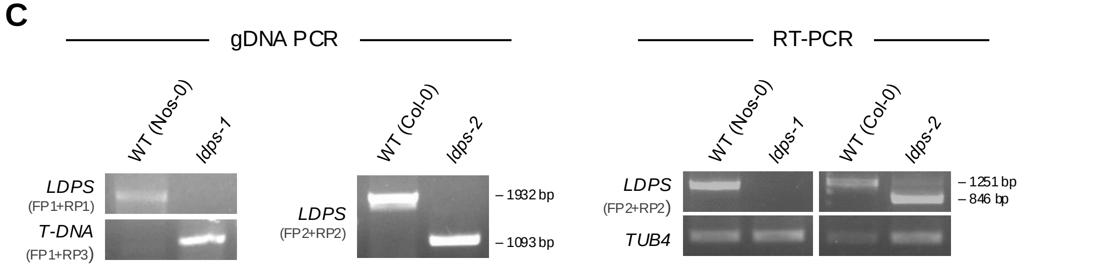


## Supplementary Figure S8

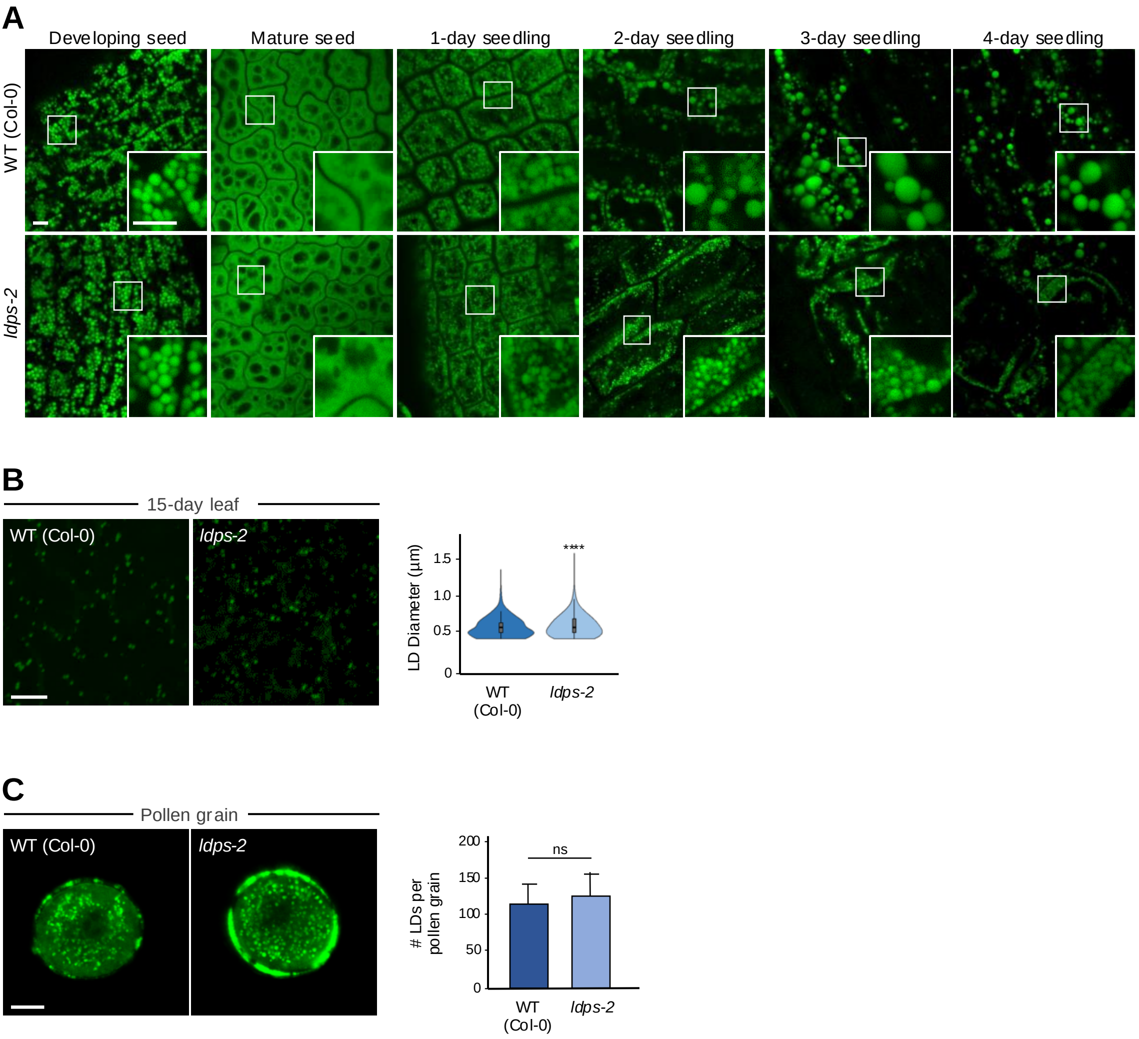


# Supplementary Figure S9

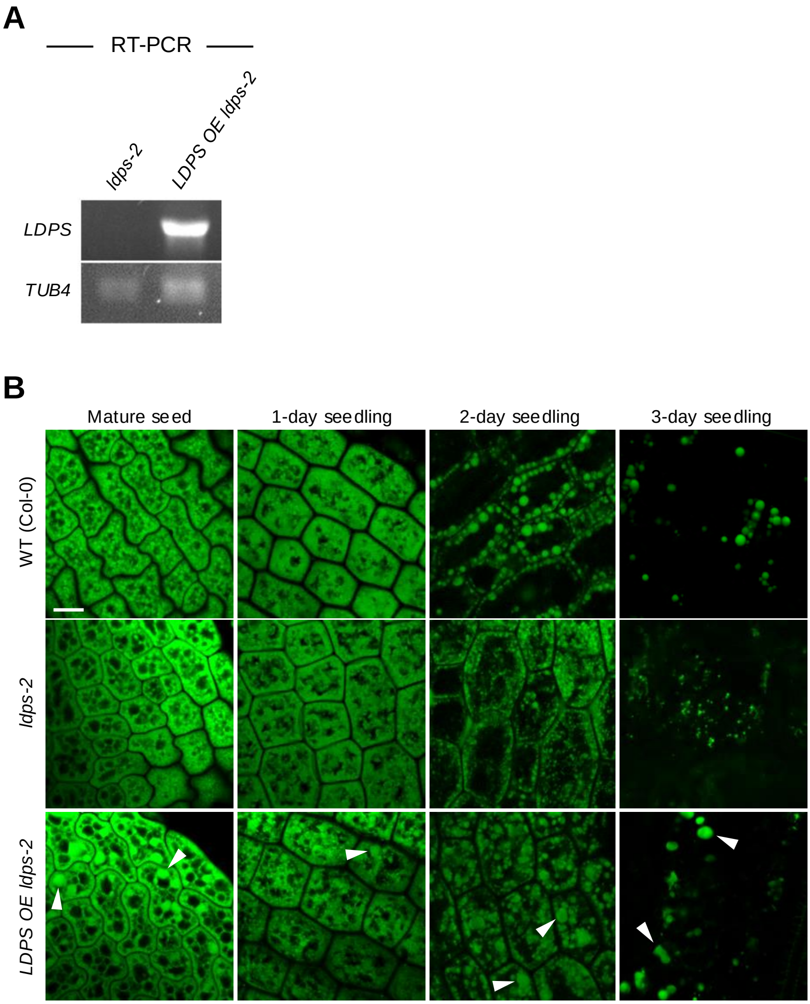


**Supplementary Figure S10**

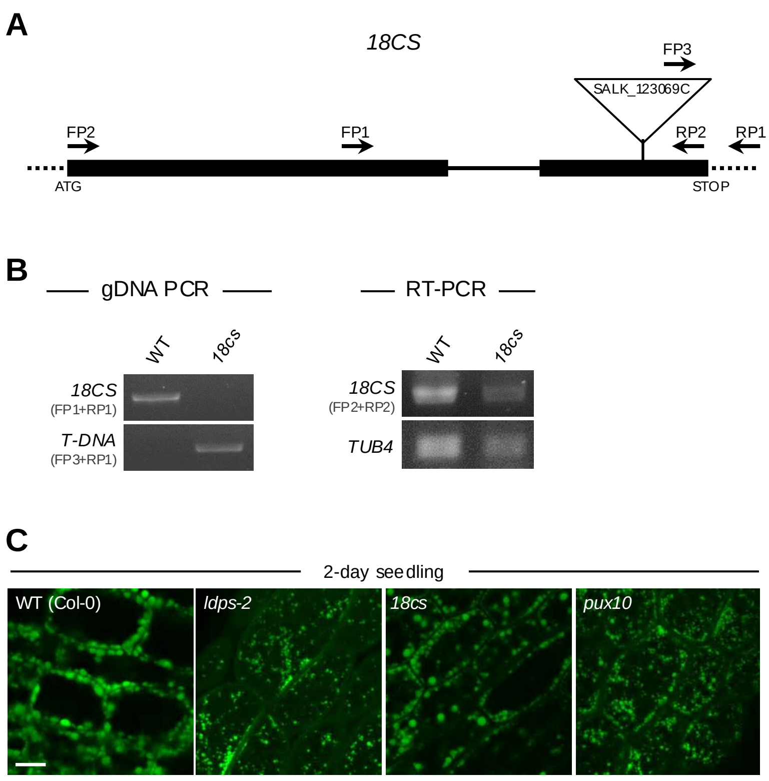


# Supplementary Figure S11

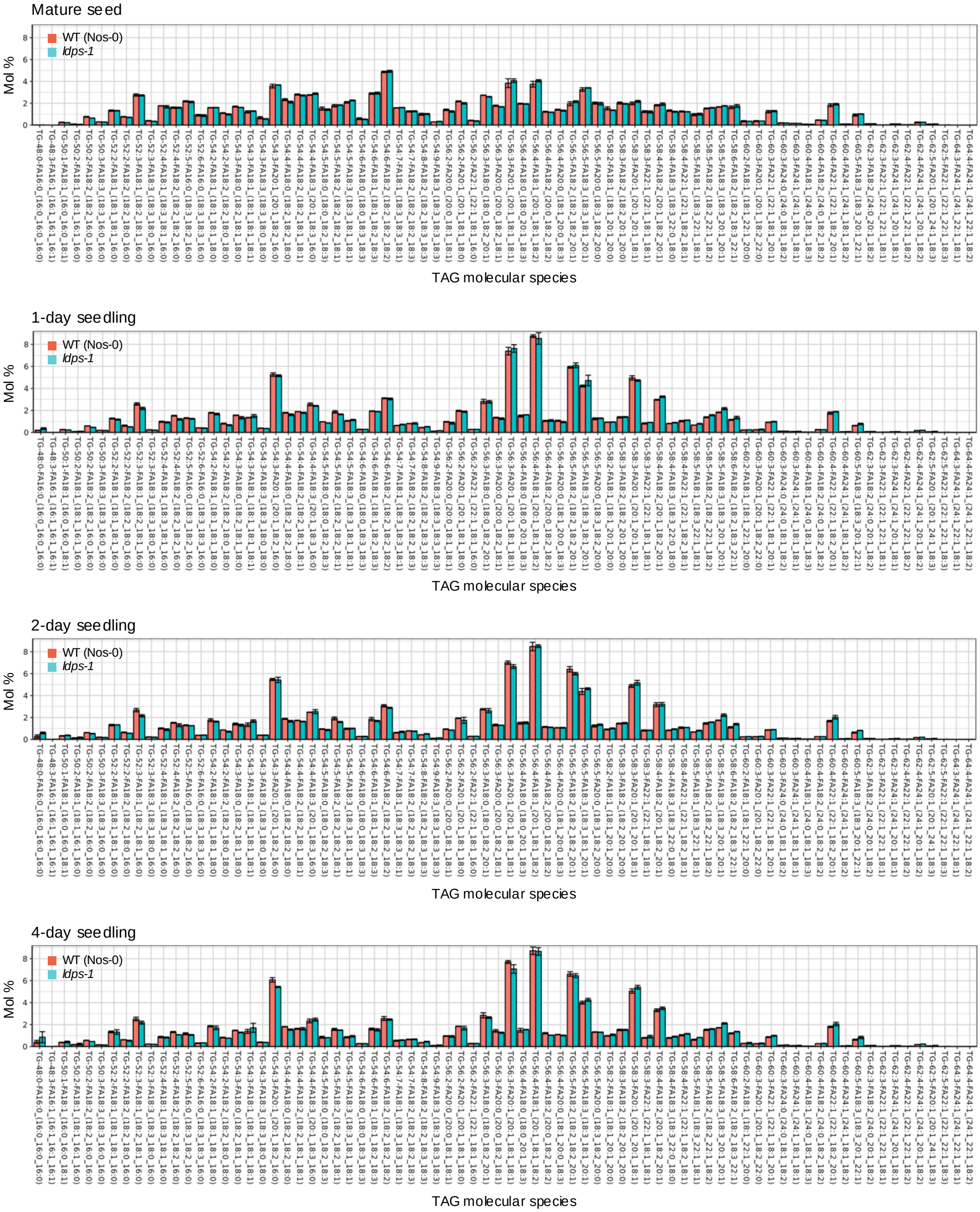


# Supplementary Figure S12

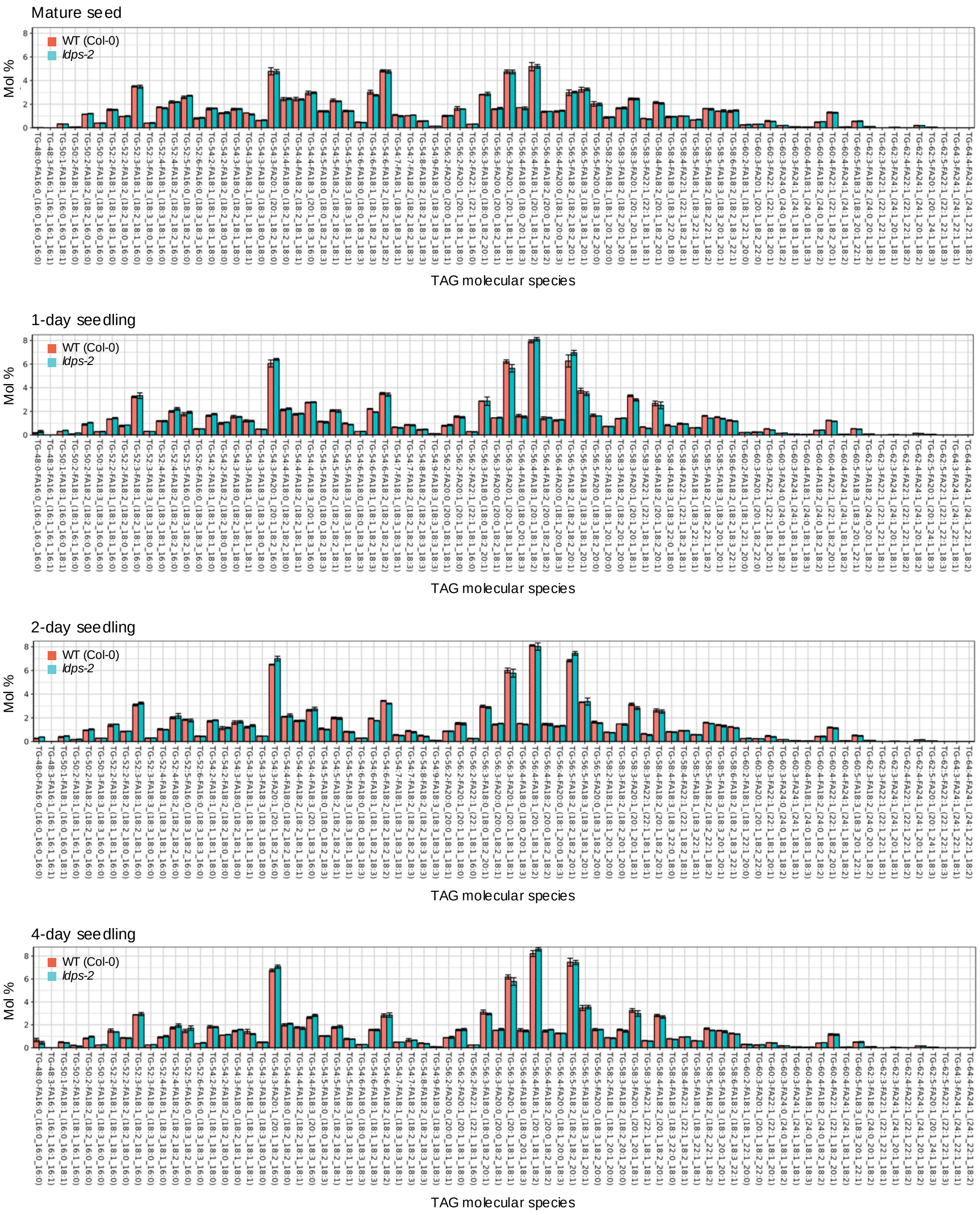


**Supplementary Figure S12 continued**

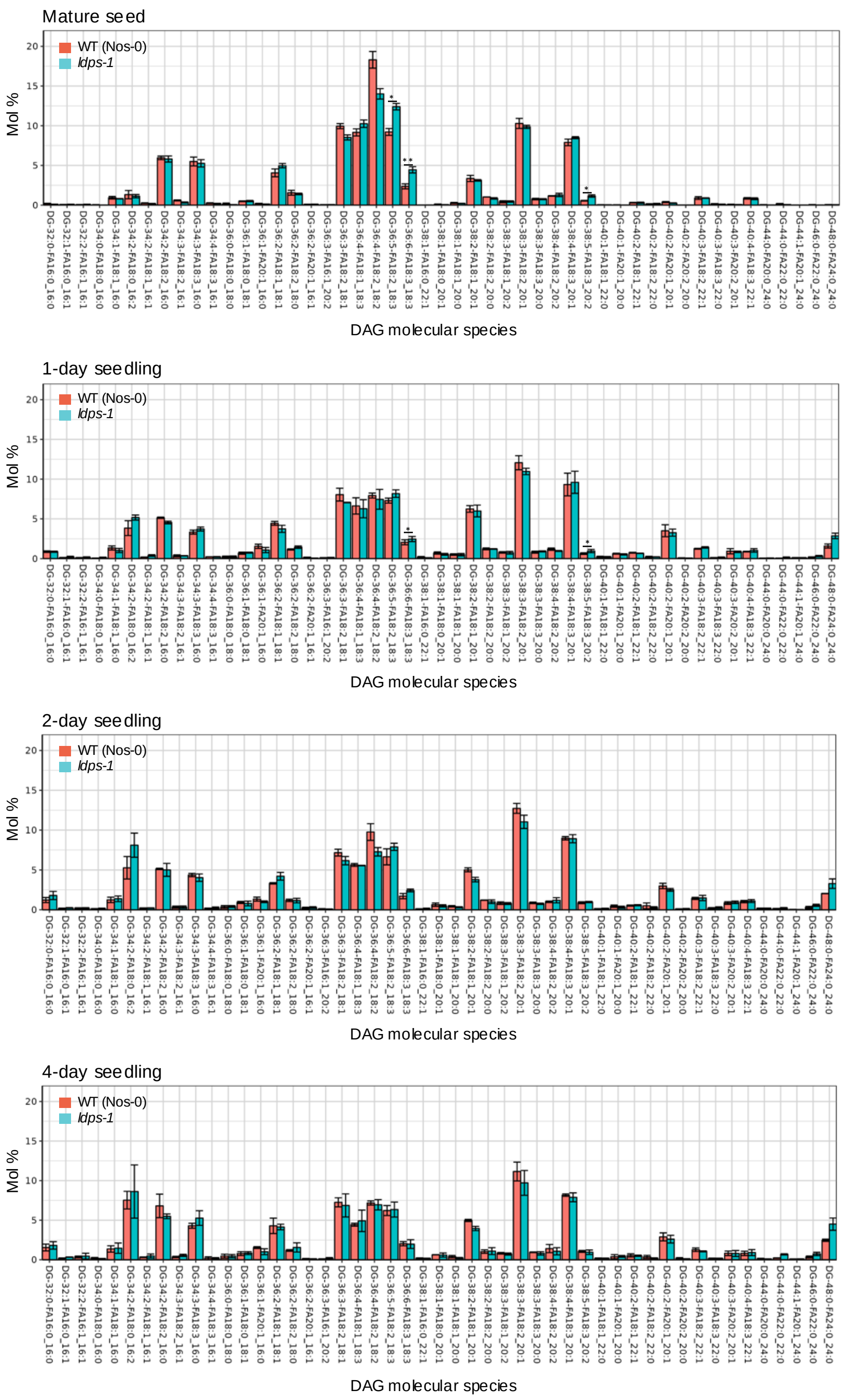


# Supplementary Figure S13

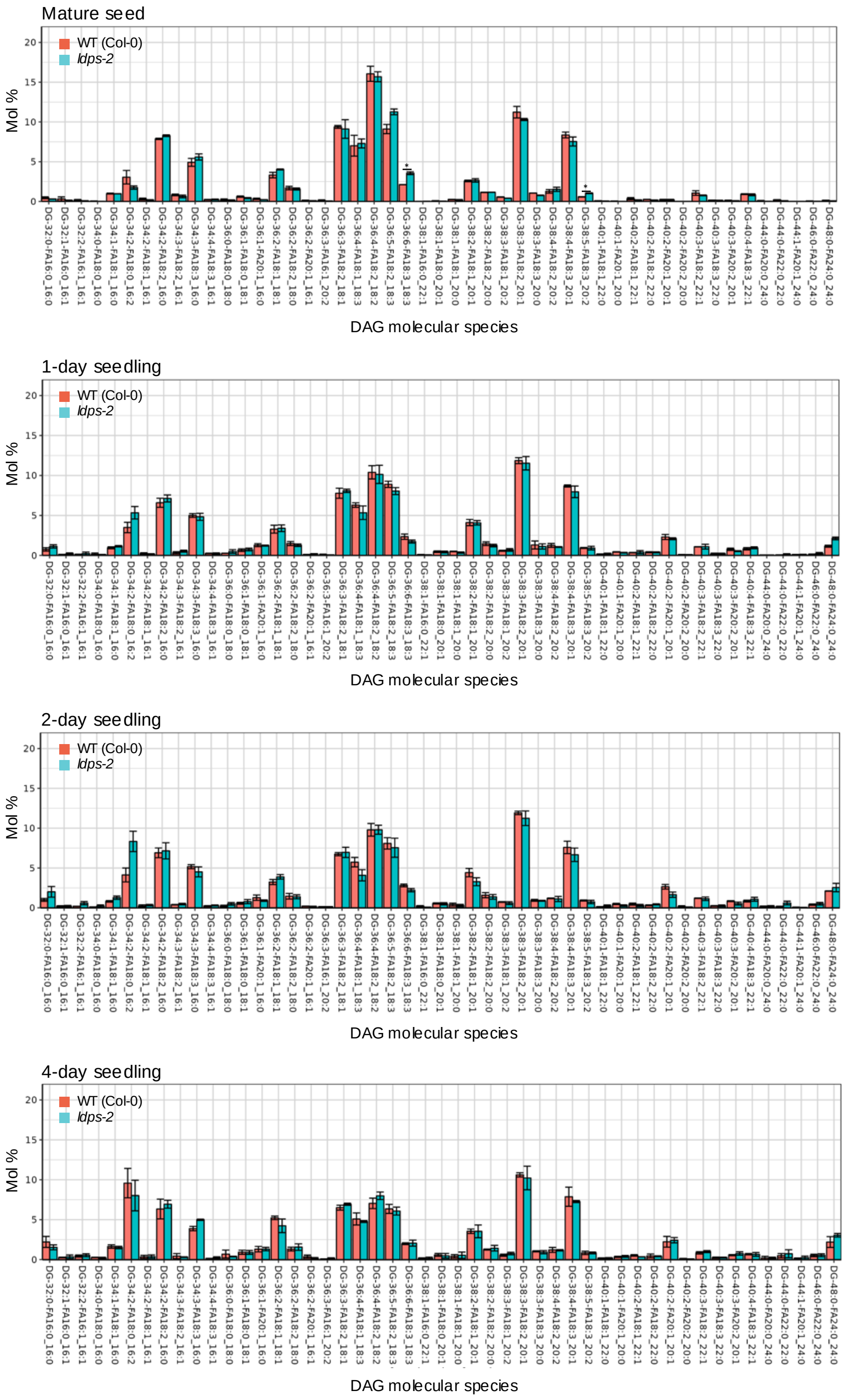


**Supplementary Figure S13 continued**

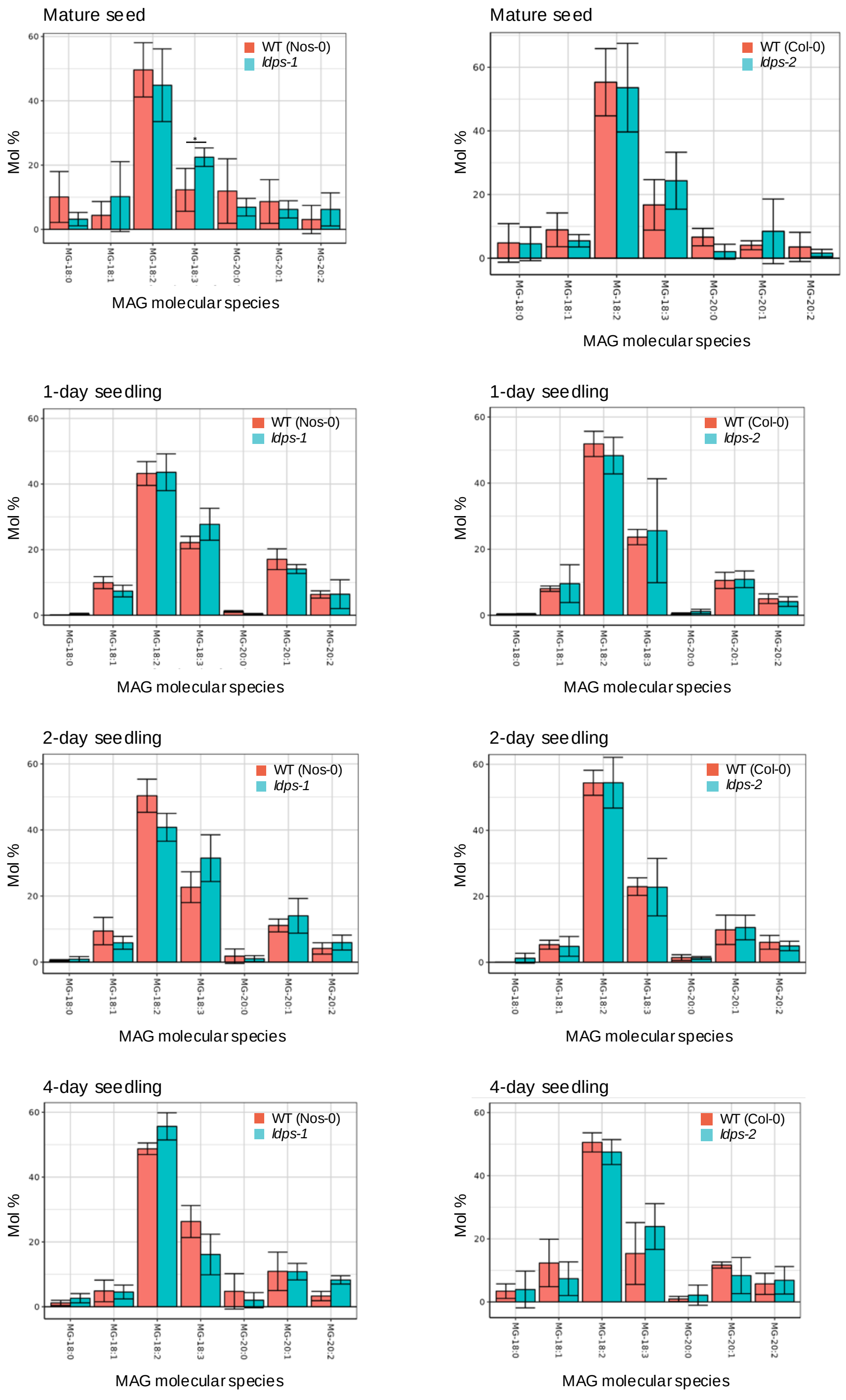


**Supplementary Figure S14**

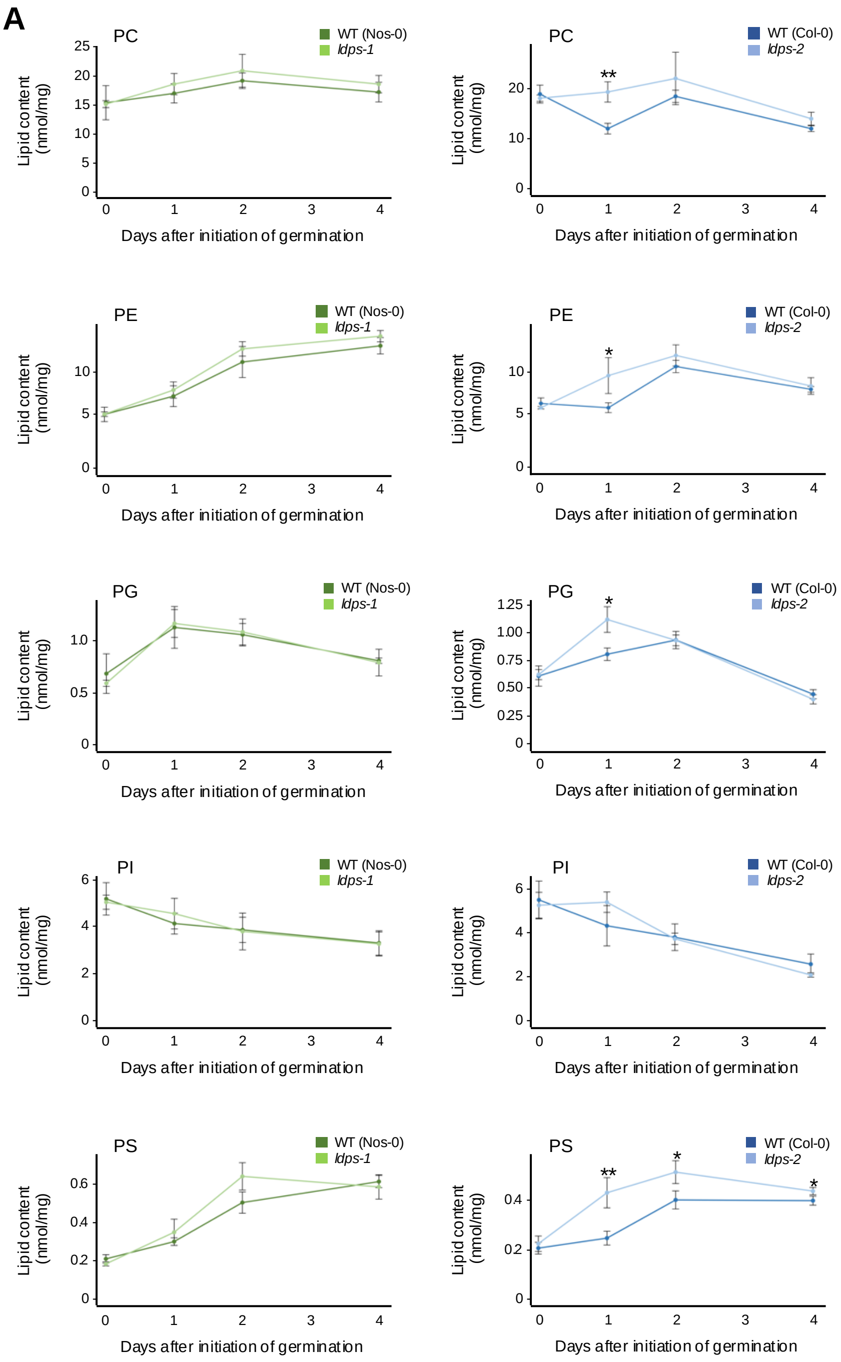


**Supplementary Figure S15**

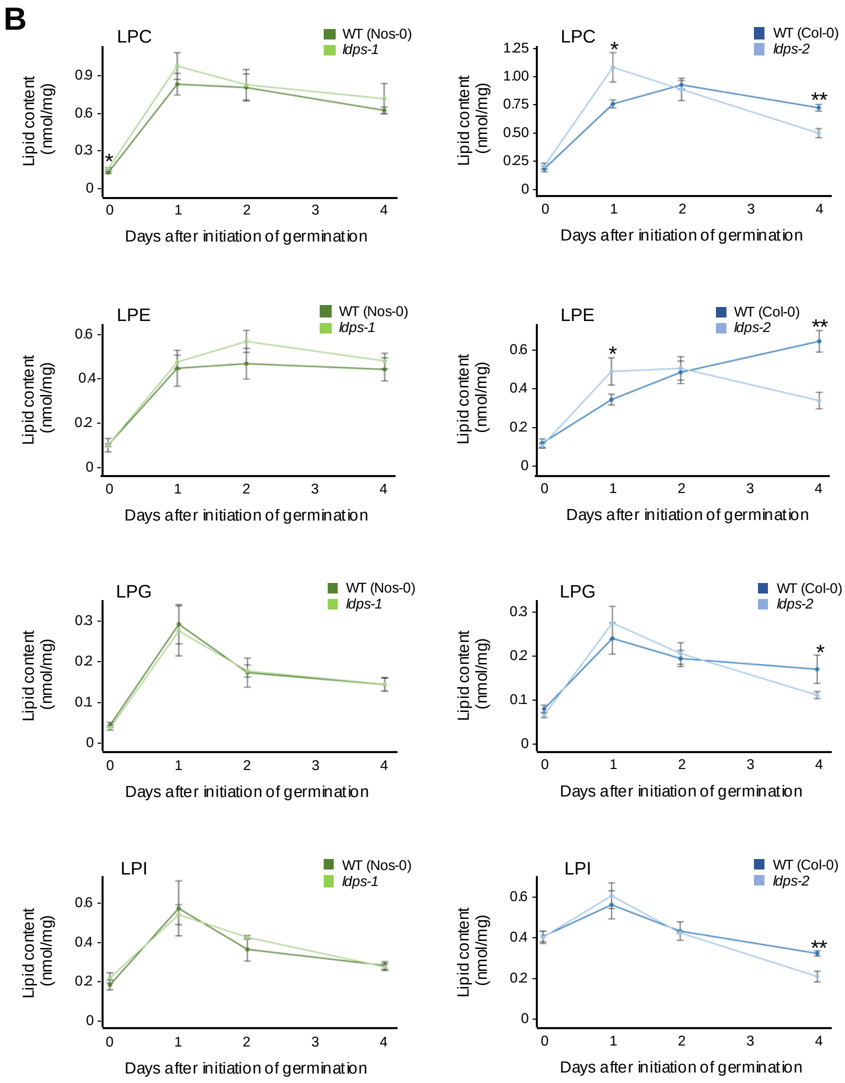


**Supplementary Figure S15 continued**

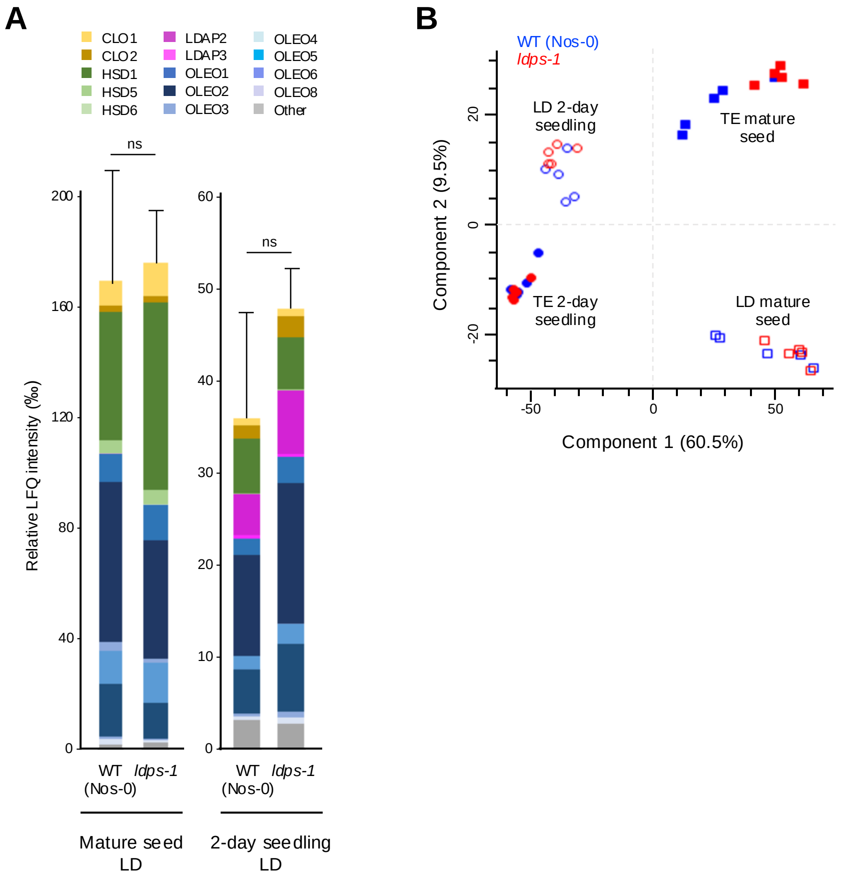


**Supplementary Figure S16**

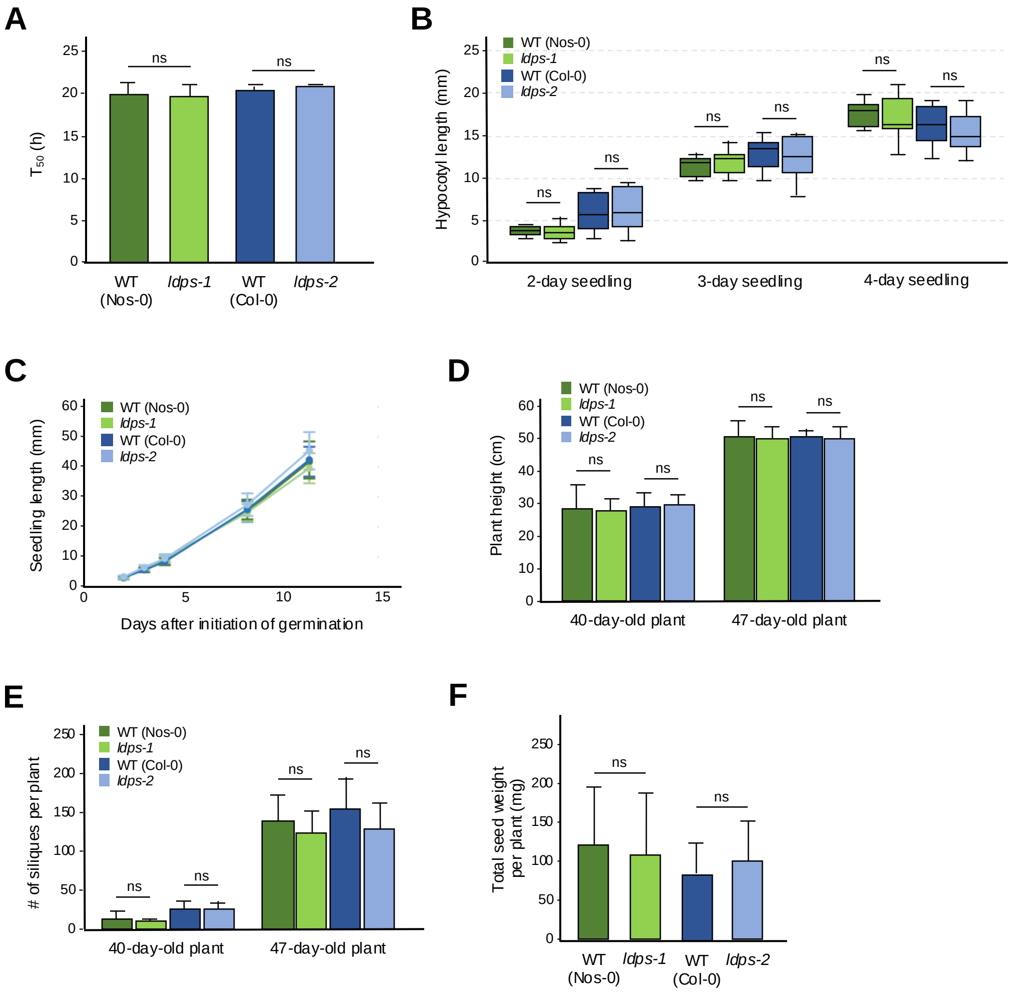


**Supplementary Figure S17**

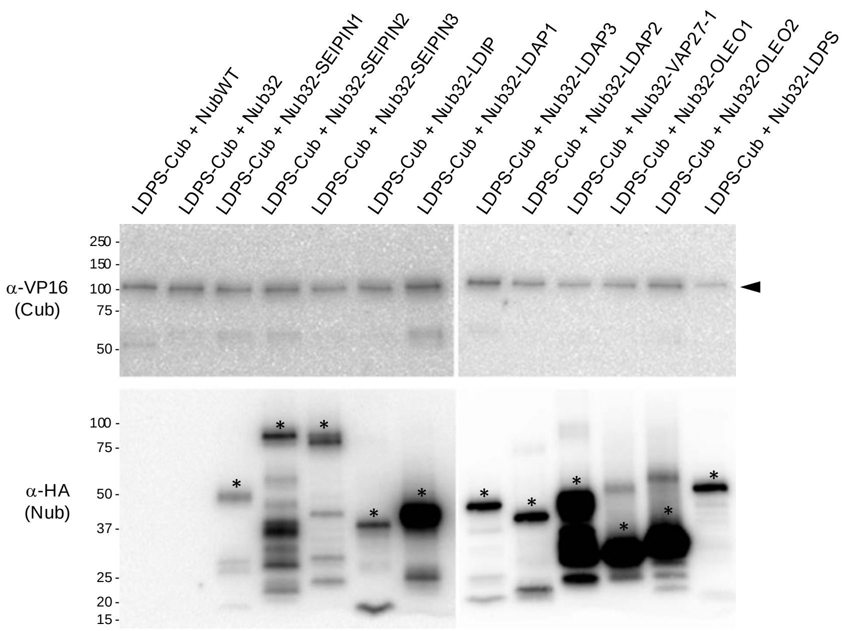


**Supplementary Figure S18**

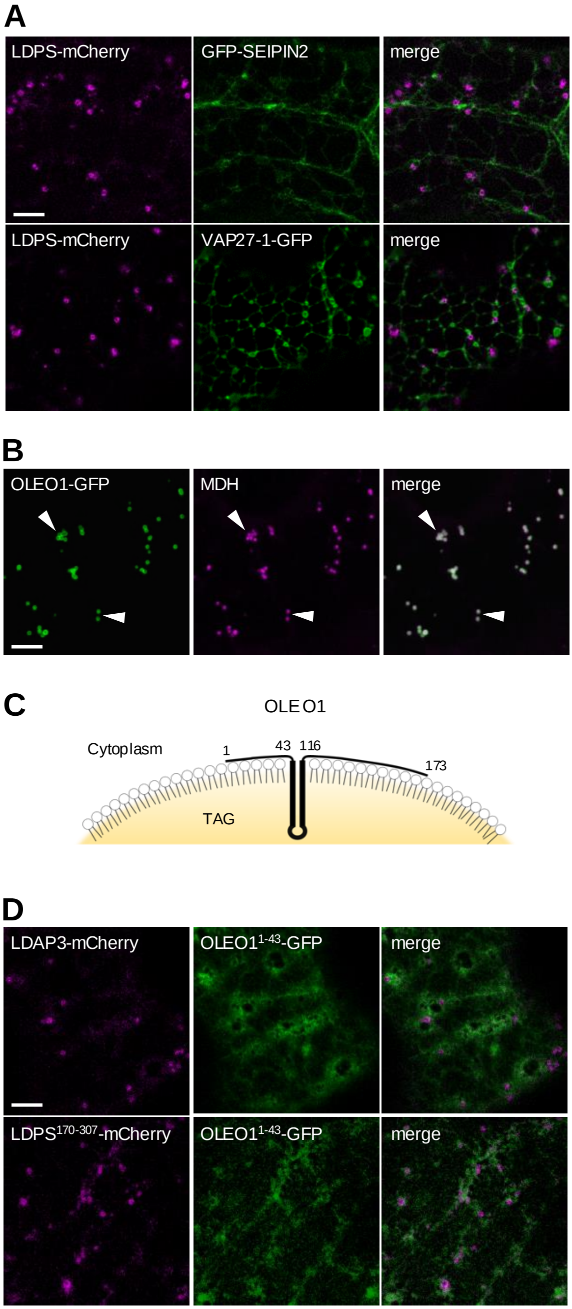


**Supplementary Figure S19**

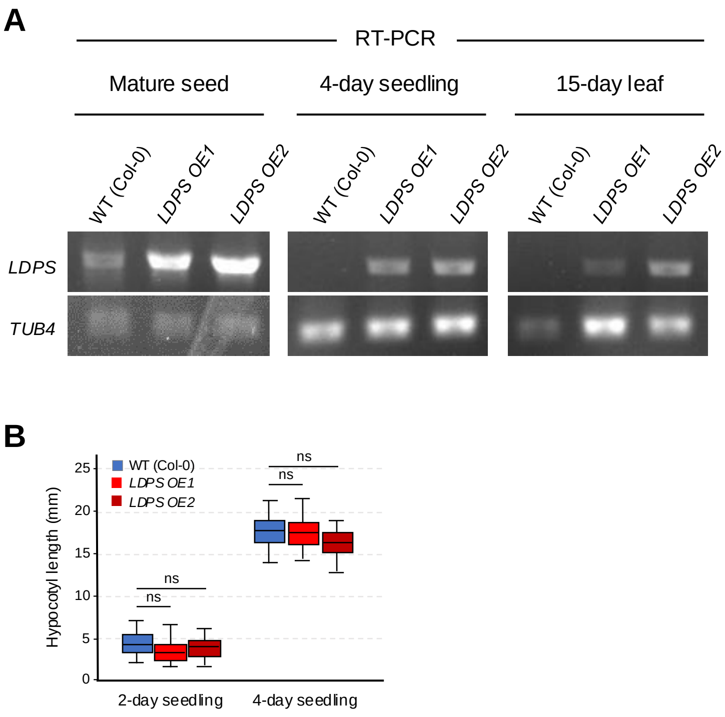


# Supplementary Figure S20

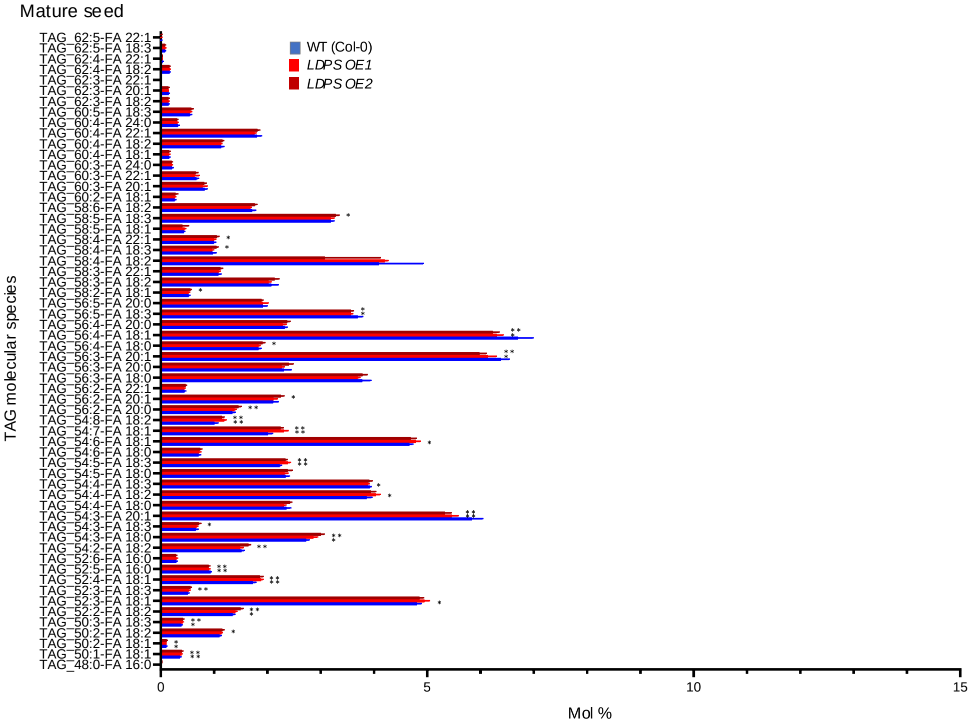


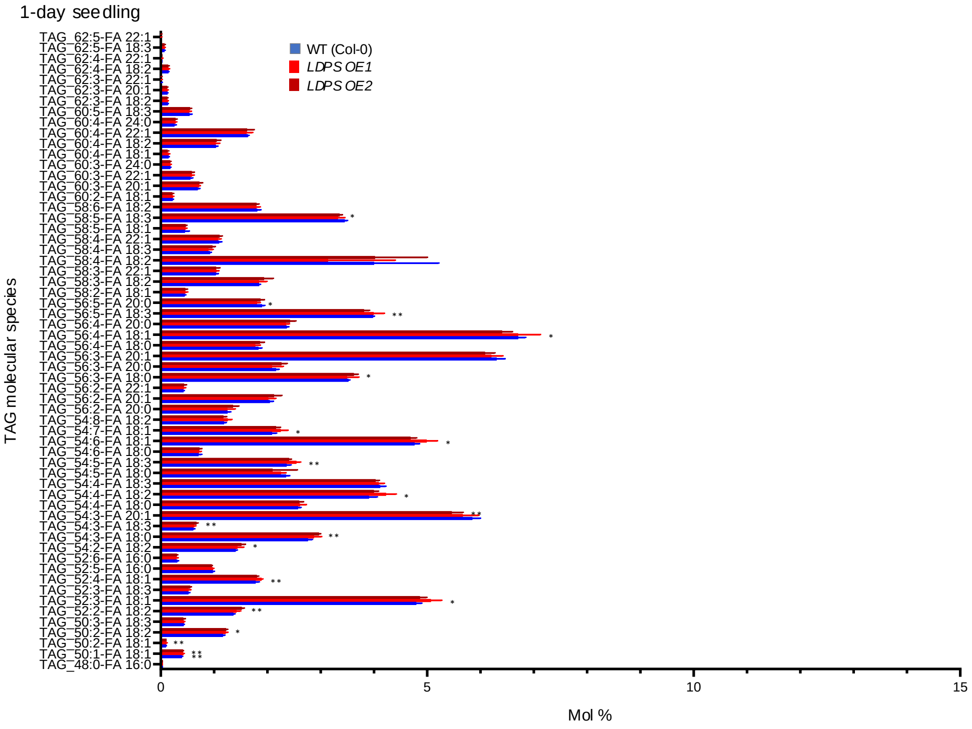


## Supplementary Figure S21

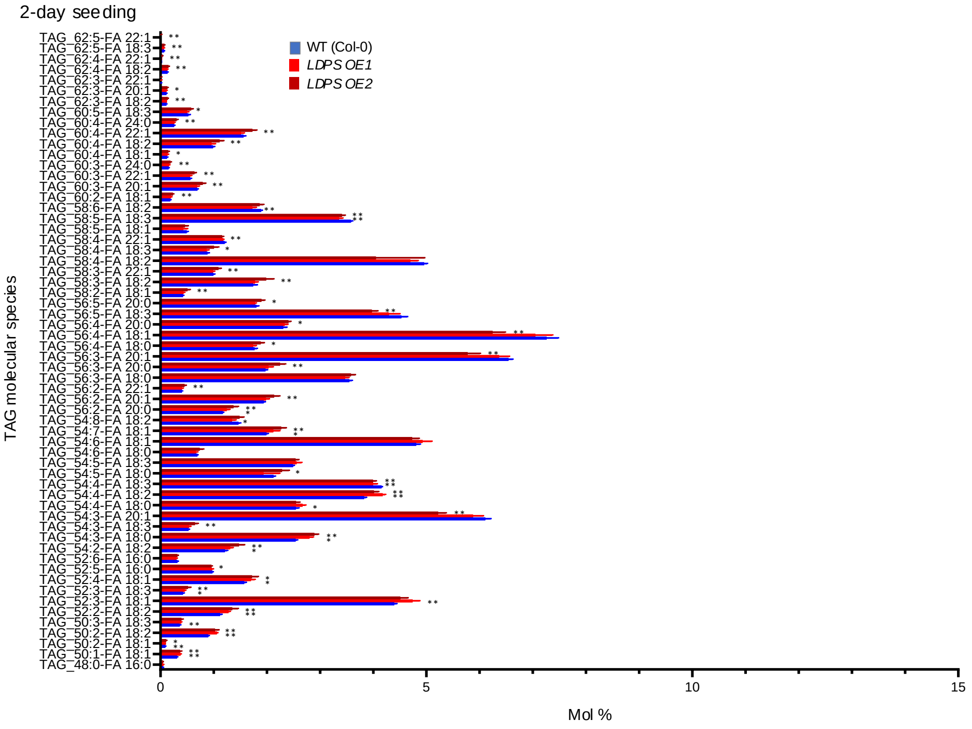


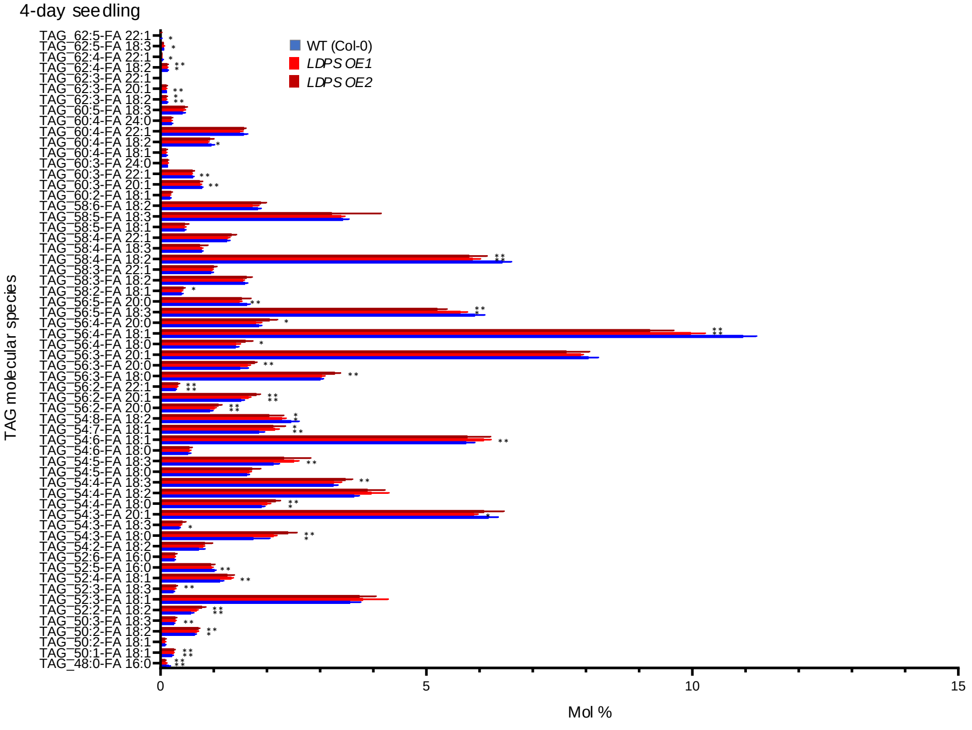


**Supplementary Figure S21 continued**

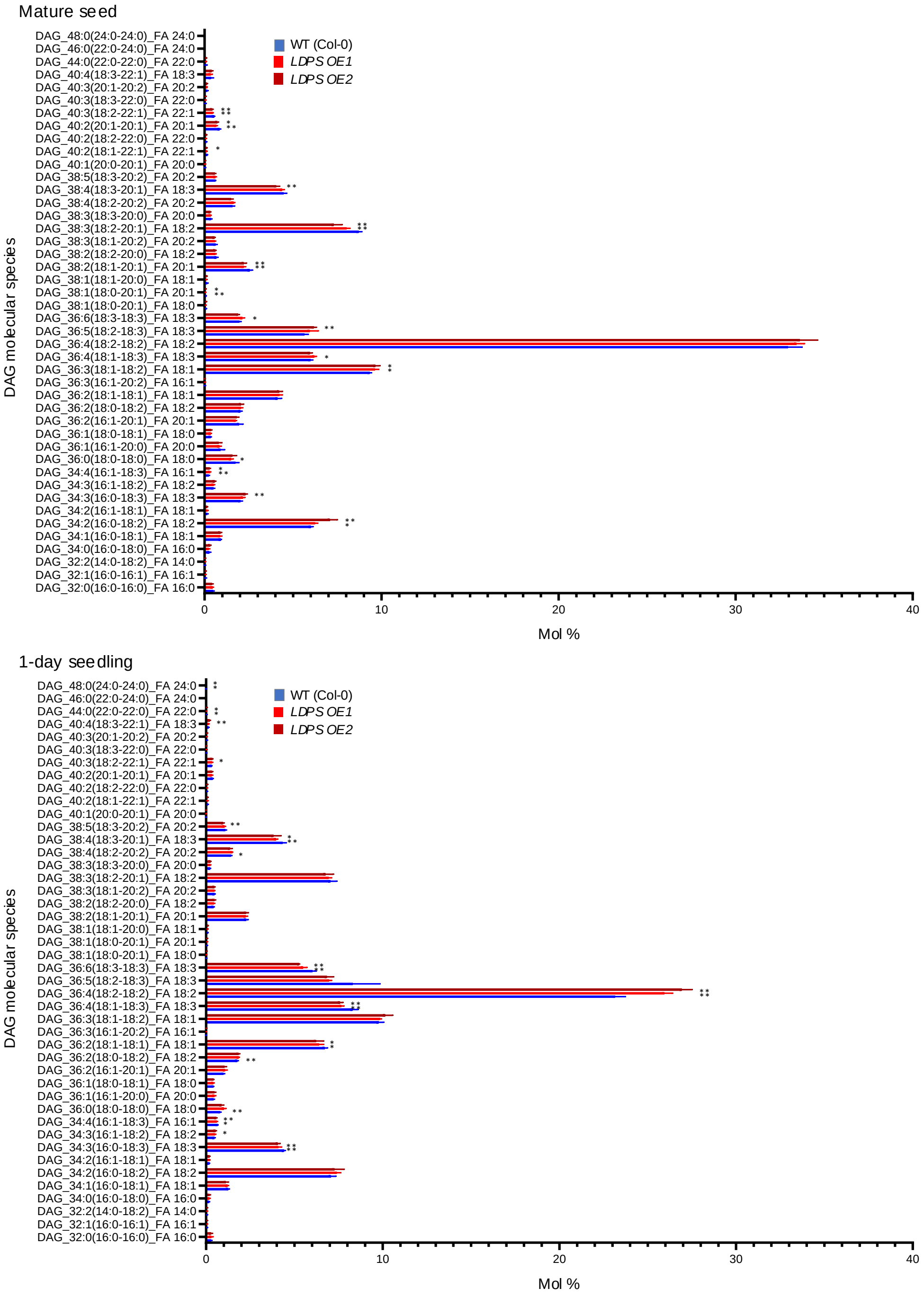


# Supplementary Figure S22

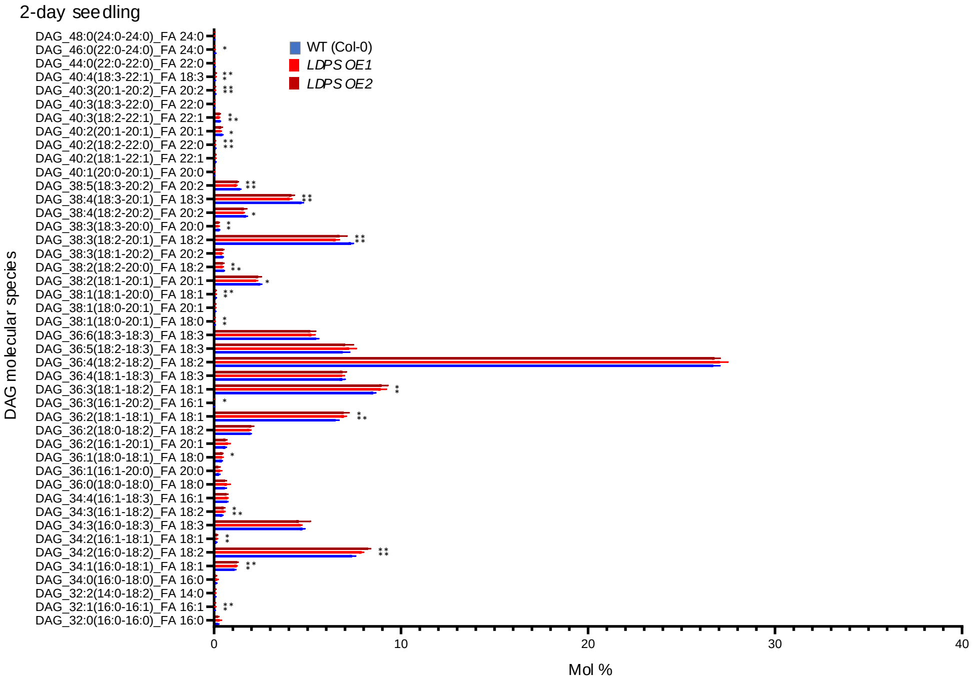


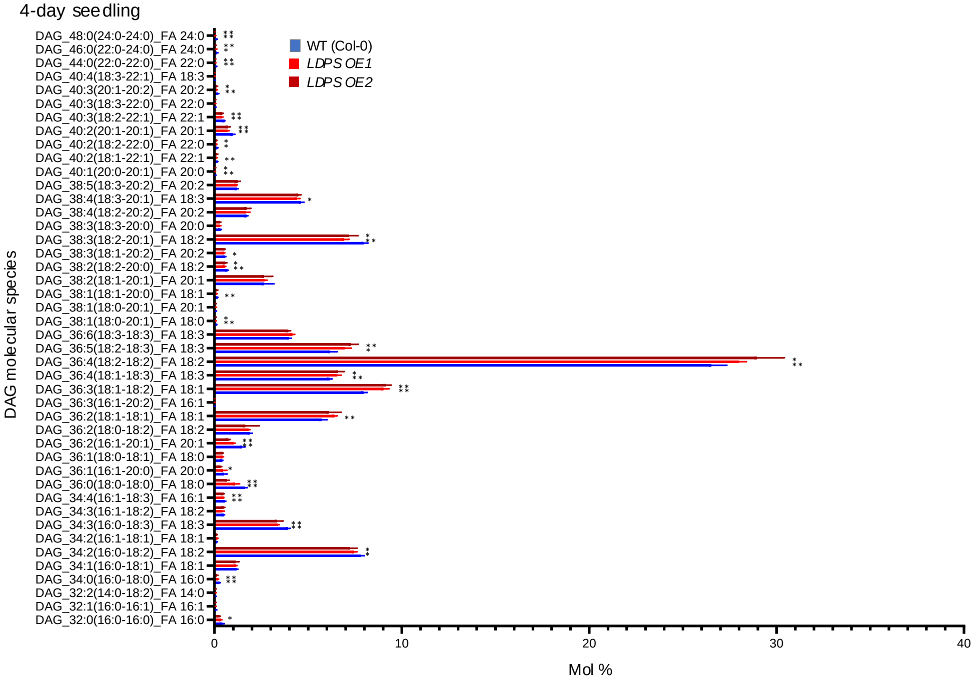


**Supplementary Figure S22 continued**

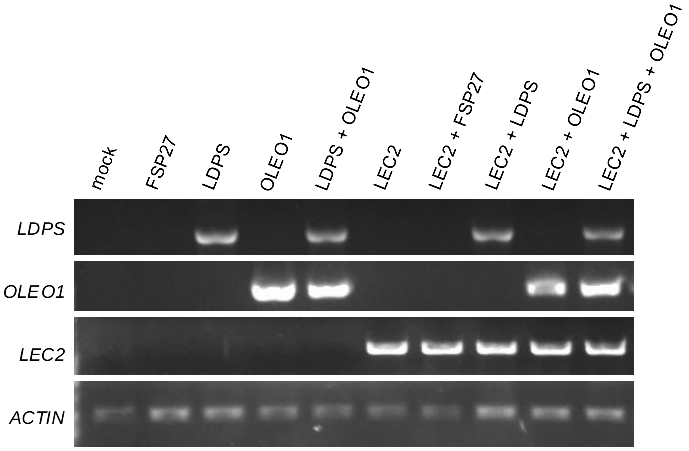


**Supplementary Figure S23**

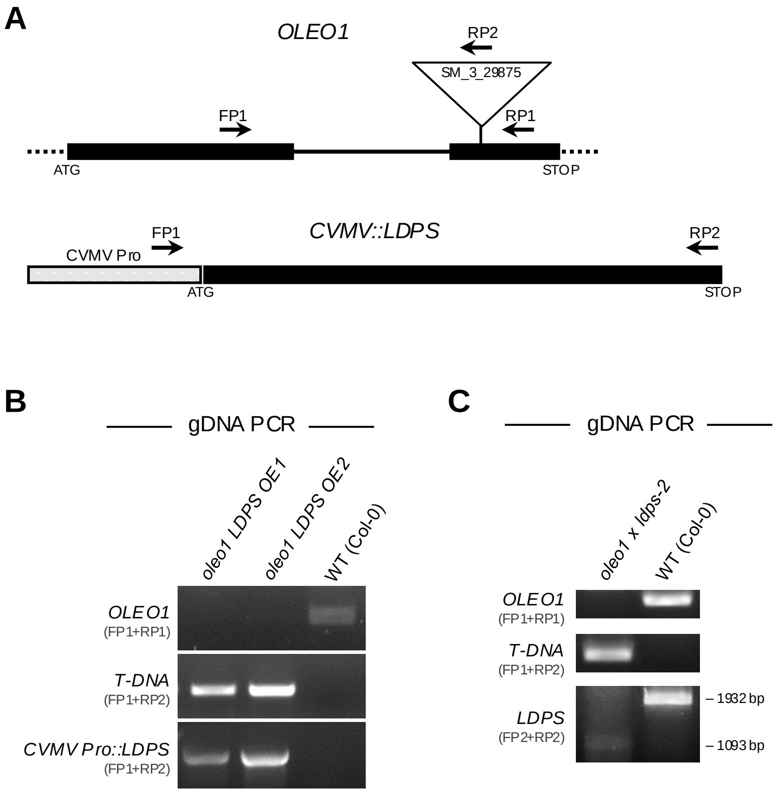


# Supplementary Figure S24

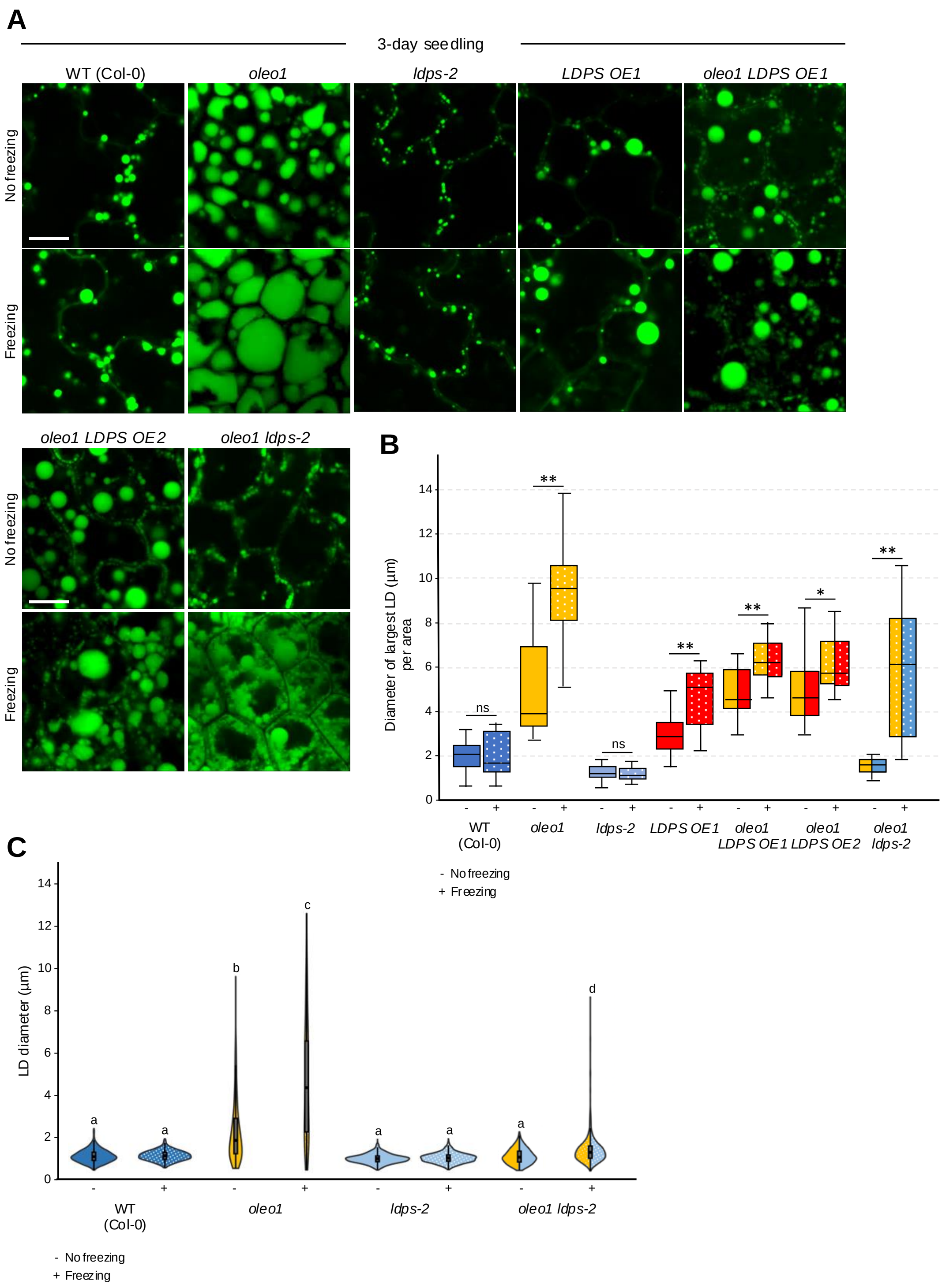


# Supplementary Figure S25

```
LDPS     MSSSRELQYLEYTYRNNRPTTGLLNSIFMTTVNTAARSLVSVASTASTPEIPSRRWSASD  60
FSP27    ----------MDYA----------------------------------------------  4
         ::*:

LDPS     HLSFASGLLTTAAENALVPAKASSSSSTSSTALVKYSGSSDLGMMICDGVDEPSVNSLGR  120
FSP27    ------------------------------------------------------------  4


LDPS     ALCHALALMNEIPVTSRKYQFAMGMAEKIMEDNAQSGHVDLLD-VNRAALASSFARTTAR  179
FSP27    ----------------------MKSLSLLY-PRSLSRHVAVSTAVVTQQLVSKPSRETPR  41
                               *   . :   .: * ** :   *.   *.*. :* * *

LDPS     LQDCLKRSRTADEPFGGLPLRVVSALPLGGYVASYVRGLSACINTVRSLADMTGNLLSQT  239
FSP27    ARPCRVSTADRKV--------------RKGIMAH---SLEDLLNKVQDILKLKDK-----  79
          : *.  :   .                 * :*.   .*.  :*.*:.: .:..:

LDPS     RRRESAVVR-AGGIQENEAELAVEKLAEELLWMTEKLRRYGAVAEGIKRWSYASGL----  294
FSP27    --PFSLVLEEDGTIVETEE--YFQALAKDTMFMVL--------L-KGQKWKPPSEQRKKR  126
            * *:.  * * *.*.   .: **:: ::*.             ::*.  *

LDPS     ASLSLTAAPRVQGLMVKISALLIGELARDSTQVPGQVTFRLLANWLPLFSHARNGLAFPV  354
FSP27    AQLALSQKPTKKID-------------------VARVTFDLY------------------  149
         *.*:*:  *. :                      .:*** *

LDPS     LTGYERVEVERAIDKAISTLPALDQEILLTNWLQDFSVSASEWPNLQPAYDRWCHSTRQL  414
FSP27    -------------------------KLNPQDFIGCLNVKATLYDTYSLSYDLHCYKAKRI  184
                                  ::   :::  :.*.*: : . . :**  *:.::::

LDPS     FM-----------------------------------------------------  416
FSP27    VKEMLRWTLFSMQATGHMLLGTSSYMQQFLDATEEEQPAKAKPSSLLPACLKMLQ  239
         .
```

# Supplementary Figure S26

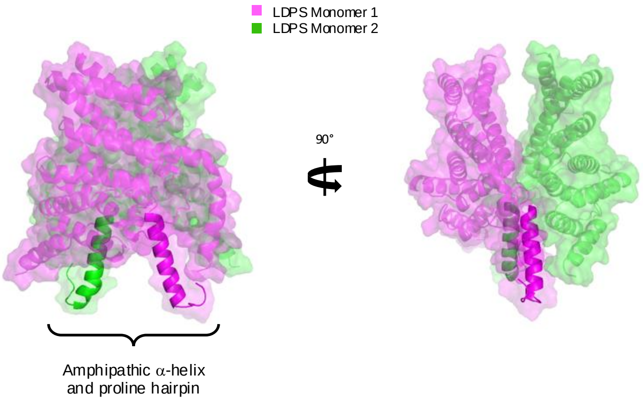


**Supplementary Figure S27**
